# A fragment merging approach towards the development of small molecule inhibitors of *Mycobacterium tuberculosis* EthR for use as ethionamide boosters†
†Electronic supplementary information (ESI) available: Experimental procedures, spectral data of new compounds, see DOI: 10.1039/c5ob02630j. Additional data related to this publication is available at the University of Cambridge data repository (https://www.repository.cam.ac.uk/handle/1810/253375).



**DOI:** 10.1039/c5ob02630j

**Published:** 2016-01-25

**Authors:** Petar O. Nikiforov, Sachin Surade, Michal Blaszczyk, Vincent Delorme, Priscille Brodin, Alain R. Baulard, Tom L. Blundell, Chris Abell

**Affiliations:** a Department of Chemistry , University of Cambridge , Lensfield Road , Cambridge , CB2 1EW , UK . Email: ca26@cam.ac.uk; b Department of Biochemistry , University of Cambridge , 80 Tennis Court Road , Cambridge , CB2 1GA , UK; c Inserm U1019 – CNRS UMR 8204 , Institut Pasteur de Lille , Université de Lille , 1 rue du Professeur Calmette , 59019 , Lille , France

## Abstract

With the ever-increasing instances of resistance to frontline TB drugs there is the need to develop novel strategies to fight the worldwide TB epidemic. Boosting the effect of the existing second-line antibiotic ethionamide by inhibiting the mycobacterial transcriptional repressor protein EthR is an attractive therapeutic strategy. Herein we report the use of a fragment based drug discovery approach for the structure-guided systematic merging of two fragment molecules, each binding twice to the hydrophobic cavity of EthR from *M. tuberculosis*. These together fill the entire binding pocket of EthR. We elaborated these fragment hits and developed small molecule inhibitors which have a 100-fold improvement of potency *in vitro* over the initial fragments.

## Abstract 2

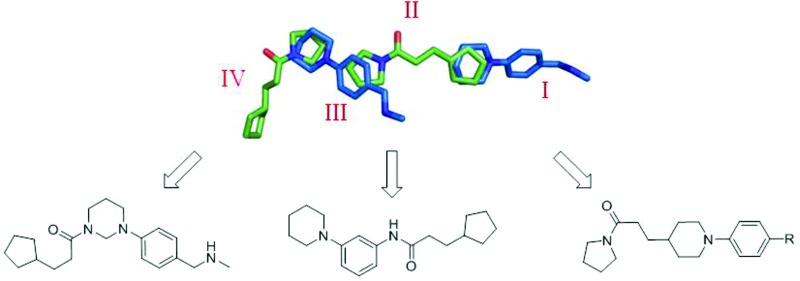
With the ever-increasing instances of resistance to frontline TB drugs there is the need to develop novel strategies to fight the worldwide TB epidemic.

## Introduction

Tuberculosis (TB) has been estimated to claim one and a half million lives worldwide each year, an epidemic that has been declared a global health emergency by the World Health Organisation (WHO).^1,2^ Despite the gravity of the situation, the treatment of active drug-susceptible (DS)-TB infection still relies on the first line antibiotics isoniazid, pyrazinamide, ethambutol and rifampicin, which were introduced over 50 years ago.^3^ There has been a concerted effort to discover new drugs to target TB that is being met with very limited success.^4,5^ An alternative therapeutic strategy is to boost the effect of existing second line TB drugs such as ethionamide.^6^

**Fig. 1:**
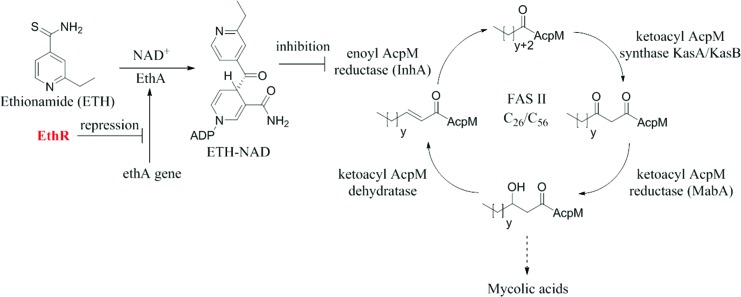
The mechanism of activation of ethionamide (ETH) and the inhibition of InhA from FASII by the ETH-NAD adduct.

Ethionamide works by targeting the 2-*trans*-enoyl reductase enzyme InhA that belongs to the type II fatty acid synthase system (FAS II) of *Mycobacterium tuberculosis* (Fig. 1).^7,8^ It is a prodrug, requiring the flavin-dependent monooxygenase enzyme EthA for its activation (Fig. 1).^9,10^ The large effective therapeutic dose and related toxicity issues of ethionamide in patients are determined in part by the mycobacterial intracellular levels of EthA, whose expression is controlled by the transcriptional repressor EthR.^11^ Small molecules, which bind to EthR, have been shown to allosterically inhibit the DNA-binding ability of the EthR dimer, thus abolishing its function as a transcriptional repressor of EthA.^6^ Previous work by Baulard *et al.* has shown that EthR binders can be used as ethionamide boosters in whole cell *M. tuberculosis* assays.^6,12–14^

In search for novel ethionamide booster scaffolds, a fragment-merging approach has been used to identify a new class of potent inhibitors of the transcriptional repressor EthR. This identified a set of fragments that occupy the entire 20 Å long hydrophobic cavity of EthR located in the drug-binding domain, playing a regulatory role in the DNA-binding.^6^ We show how these fragments can be systematically merged to afford potent EthR ligands. Surface plasmon resonance (SPR) is used as a functional assay^6,11^ to demonstrate the ability of the merged compounds to disrupt the interaction between the transcriptional repressor, EthR, and its DNA operator. A range of other biophysical techniques, including fluorescence-based thermal shift,^15^ ITC,^16^ and X-ray crystallography^17^ are used to further validate the binding of the merged compounds to EthR.

**Fig. 2:**
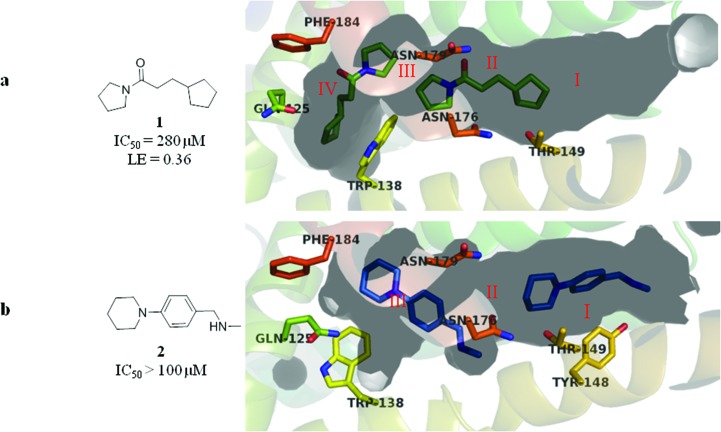
(a) X-ray crystal structure of fragment **1** bound to sub-pockets II and IV of the EthR binding cavity. LE values throughout this paper are calculated using IC_50_ values determined by SPR. (b) X-ray crystal structure of fragment **2** bound to sub-pockets I and III of the EthR binding cavity. The numbers I, II, III and IV denote the four sub-pockets, into which the binding cavity of EthR can be divided according to the four distinct binding positions of fragments **1** and **2**. (PDB codes ; 5F1J and ; 5F27 respectively).

Previously, we reported screening of a 1250-member fragment library against EthR where 86 fragment molecules were identified using fluorescence-based thermal shift, SPR and ligand-based NMR.^18^ A fragment was considered a hit if it raised the melting temperature (*T*_m_) in the thermal shift assay of EthR by more than 1 °C when used at a concentration of 5 mM.^18^ Two of the fragment hits, **1** and **2**, were shown by X-ray crystallography to bind twice to the EthR monomer (Fig. 2a and b).‡
‡Protein X-ray crystallography structures of compounds **1–5**, **14**, **15**, **21**, **22** and **28** bound to *M. tuberculosis* EthR are available *via* the RCSB Protein Data Bank *via* PDB codes: 5F1J, 5F27, 5F04, 5F0C, 5EYR, 5F08, 5F0F, 5EZH, 5EZG, 5F0H.


**Fig. 3:**
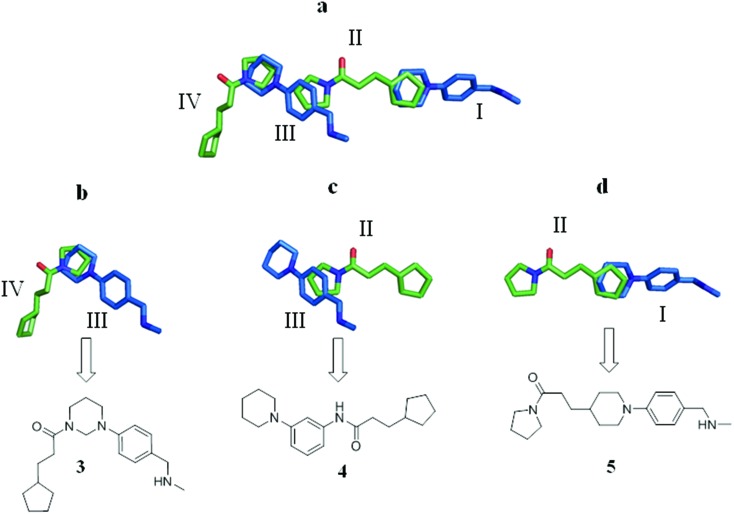
(a) Overlay of the X-ray crystal structures of fragments **1** (green) and **2** (blue), which together span the entire length of the EthR binding cavity and bind in four distinct sub-pockets denoted I, II, III and IV; (b–d) molecular structures **3**, **4** and **5** arising from the merging of two adjacent overlapping fragment units of **1** and **2** bound to EthR.

One molecule of **1** bound within a polar surface area hotspot (sub-pocket II), where the side chains of residues Asn179, Asn176 and Thr149 are located. The second molecule of **1** bound in a cryptic sub-pocket (IV) situated in the innermost region of the EthR binding cavity. This is in contrast to the fragment hit **2**, where the two molecules interact with Asn176 (sub-pocket III) and the hydroxyl group of Tyr148 (sub-pocket I) respectively. Together fragments **1** and **2** span the entire length of the EthR hydrophobic cavity and represent attractive starting points (Fig. 3a) for fragment merging towards the synthesis of more potent EthR ligands.

### Fragment merging strategy I

Based on the overlay of the crystal structures of fragments **1** and **2** bound to EthR (Fig. 3a), Fig. 3b–d summarise possible structures of compounds arising from the direct merging of two adjacent fragments in the EthR binding cavity. Examination of the X-ray crystal structures shows that the cyclopentyl ring of fragment **1** and the piperidine ring of fragment **2** bound to EthR are aligned sufficiently to allow them to be used as the site of merging of the two units to obtain molecule **5** (Fig. 3d). The left hand side of ligand **1** (the pyrrolidine amide) could also be modified by merging the structures of fragment **2** bound to sub-pocket III of EthR with the molecule of **1** residing in sub-pocket II of the protein (Fig. 3c). The final two-unit fragment-merging strategy involves combining the structures of fragment **1** (sub-pocket IV) of EthR with the unit of **2** residing in sub-pocket III of the protein as shown in Fig. 3b. The pyrrolidine ring of **1** and the piperidine ring of **2** in this configuration (Fig. 3b) are well aligned and this region of overlap is the site of merging of the two units to obtain a hybrid molecule such as **3**.

**Fig. 4:**
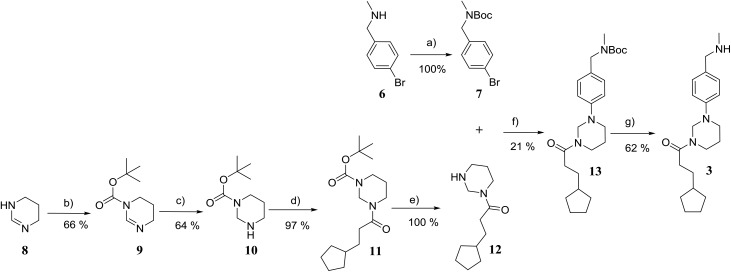
Synthetic scheme for the preparation of 3-cyclopentyl-1-(3-(4-((methylamino) methyl)phenyl) tetrahydropyrimidin-1(2*H*)-yl)propan-1-one (**3**). (a) di-*tert*-butyl dicarbonate, NEt_3_, THF; 0 → 22 °C; overnight; (b) di-*tert*-butyl dicarbonate, NEt_3_, THF; 0 → 22 °C; overnight; (c) NaBH_4_, MeOH, 0 °C; 2 h; (d) 3-cyclopentylpropionic acid, DCM, diisopropylethylamine, COMU, 22 °C; 18 h; (e) TFA, DCM, 22 °C; 2 h; (f) Pd(OAc)_2_, KO^*t*^Bu, 2-(di-*tert*-butylphosphino) biphenyl, toluene; 100 °C, 4 h; (g) TFA, DCM, 22 °C; 2 h.

Initially, compounds **3**, **4** and **5** were synthesised. The synthesis of compound **3** is shown in Fig. 4. Further synthetic schemes and experimental procedures for the synthesis of molecules **4** and **5**, as well as for all other compounds discussed are described in the ESI.†

The synthesis of compound **3** (Fig. 4) started with the Boc protection of 1,4,5,6-tetrahydropyrimidine **8** to give intermediate **9**. Sodium borohydride reduction of **9** afforded *tert*-butyl tetrahydropyrimidine-1(2*H*)-carboxylate **10**,^19^ which was coupled with 3-cyclopenane propionic acid using COMU to give intermediate **11**.^20^ The deprotection of **11** with trifluoroacetic acid in dichloromethane afforded 3-cyclopentyl-1-(tetrahydropyrimidin-1(2*H*)-yl)propan-1-one **12** in near quantitative yield. The coupling of **12** with *tert*-butyl (4-bromobenzyl)(methyl)carbamate under Buchwald–Hartwig conditions^21^ and subsequent Boc deprotection of the resulting intermediate **13** gave the target compound **3** in 5% yield over six steps.

**Fig. 5:**
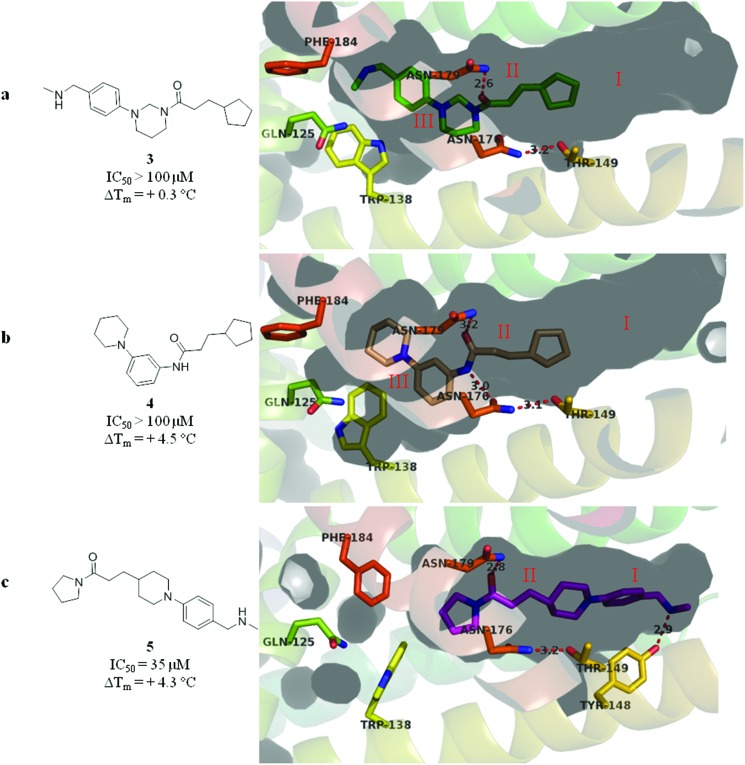
(a–c) X-ray crystal structures of merged compounds **3**, **4** and **5** bound to EthR. IC_50_ (SPR) and Δ*T*_m_ (DSF) values for the three ligands are also shown. (PDB codes ; 5F04, ; 5F0C and ; 5EYR).

The merged compounds **4** and **5** increased the melting temperature of EthR by +4.5 °C and +4.3 °C respectively when screened at a concentration of 100 μM. The merged ligand **5** also showed an eight-fold increase in the disruption of the interaction between EthR and its DNA operator as measured by SPR when compared to the starting fragment **1**. Most significantly the structures of **3**, **4**, and **5** bound to EthR were determined by X-ray crystallography (Fig. 5).

The good overlap between the X-ray crystal structures of ligands **4** and **5** (Fig. 5b and c respectively) and their parent fragments **1** and **2** fully justifies the merging operations used to construct these two ligands. In contrast, X-ray crystallography showed that compound **3** soaks into sub-pockets II and III of EthR (Fig. 5a) and does not span sub-pockets IV and III as intended by design (Fig. 3b). This could be attributed to the more favourable polar interactions available to the amide functionality of compound **3** in the vicinity of the polar uncharged amino acid Asn179 located in sub-pocket II. Thus the carbonyl oxygen atom of **3** is capable of interacting with the side chain of residue Asn179 through a well defined hydrogen bond. The analogous polar interaction between the carbonyl oxygen atom of the starting fragment **1** and Asn179 (2.8 Å, Fig. 2a) is also observed in the X-ray crystal structures of the EthR-bound complexes of ligands **4** (Fig. 5b) and **5** (Fig. 5c).

Subsequent exploration of SAR around compound **5** (summarised in Table 1) yielded ligands with significantly higher affinity towards EthR than the parent fragments **1** and **2**. The original merged ligand **5** (IC_50_ = 35 μM) showed an eight-fold improvement in binding affinity towards EthR compared to its parent fragment **1** (IC_50_ = 280 μM). Interestingly, compound **14**, the Boc protected synthetic precursor of **5**, gave a further five-fold improvement in affinity by SPR (*K*_D_ = 3 μM (ITC) and IC_50_ = 7 μM) compared to the originally-designed merged compound **5** (IC_50_ = 35 μM).

**Table 1:** Exploration of SAR around compound **5**. Fluorescent-based thermal shift (Δ*T*_m_) values against the EthR target (at 100 μM concentration of compound), IC_50_ values measured by SPR and binding affinities (*K*_D_) determined by ITC are given where available (n. d. = no heats of binding detected)

Compound number	Compound structure	Δ*T*_m_/°C	IC_50_/ μM (SPR)	*K*_D_/ μM (ITC)
**1**	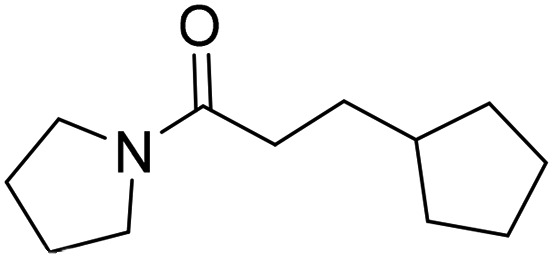	+3.5	280	12
**2**	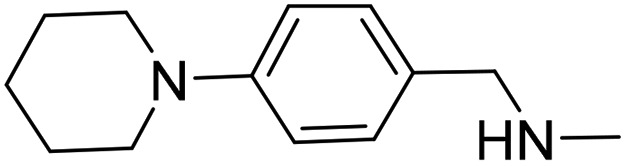	+3.8	>100	n.d.
**5**	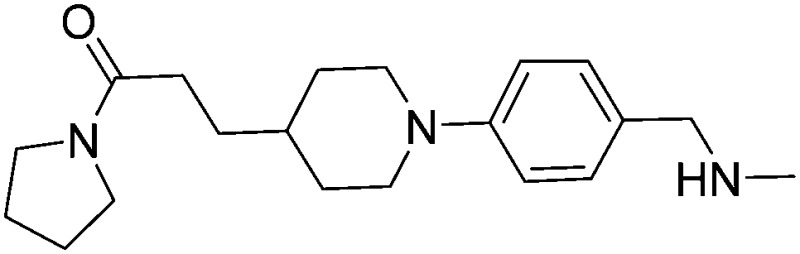	+4.3	35	5
**14**	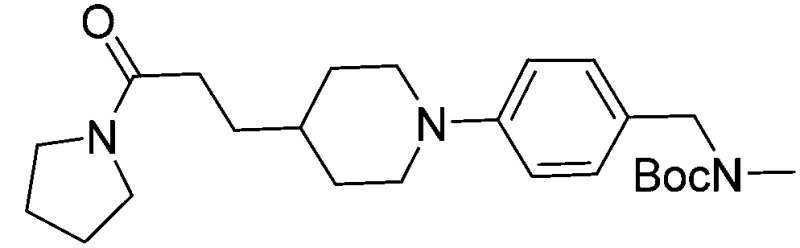	+6.3	7	3
**15**	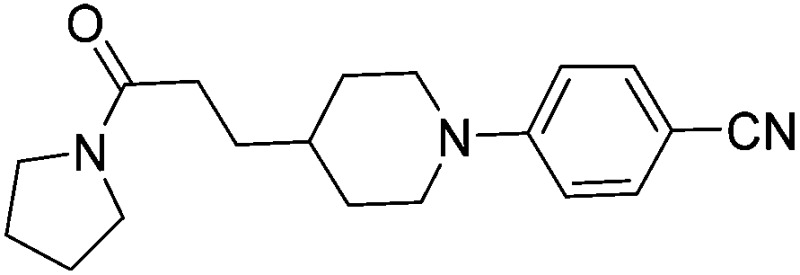	+7.5	3	1
**16**	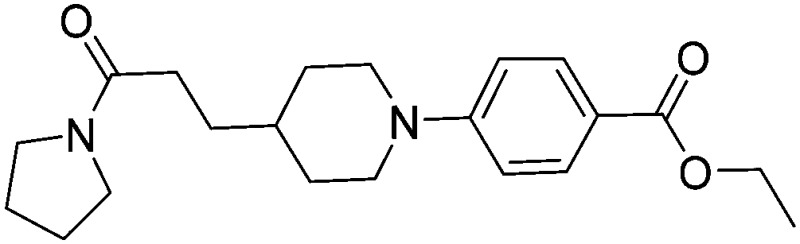	+9.3	3	n.d.
**17**	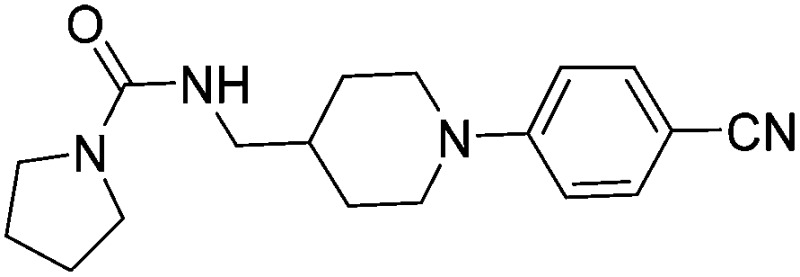	+7.2	4	n.d.
**18**	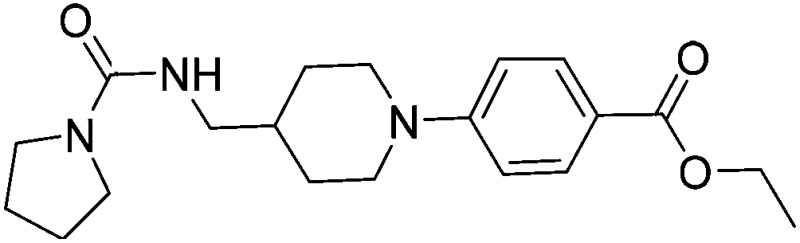	+8.8	2	n.d.
**19**	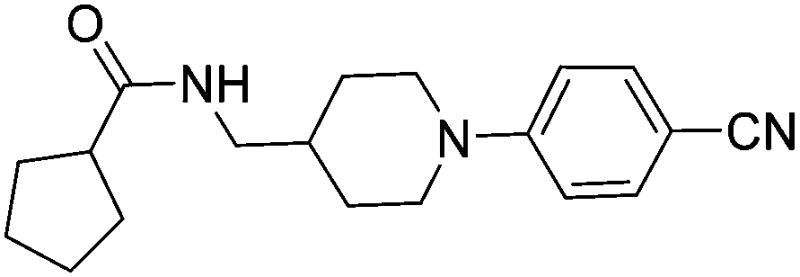	+5.9	25	n.d.
**20**	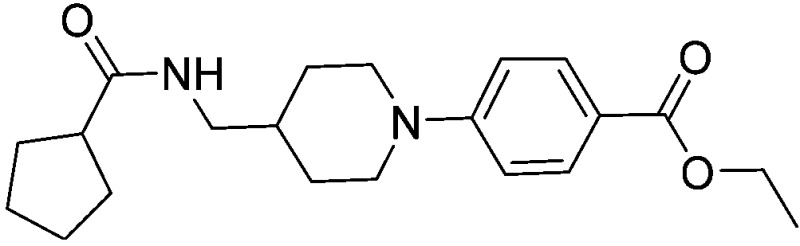	+5.5	>100	n.d.
**21**	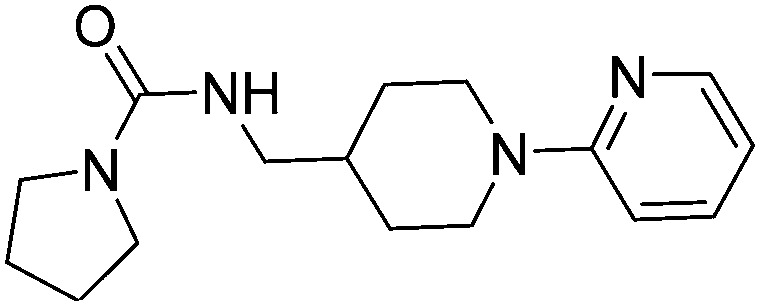	+3.8	22	24
**22**	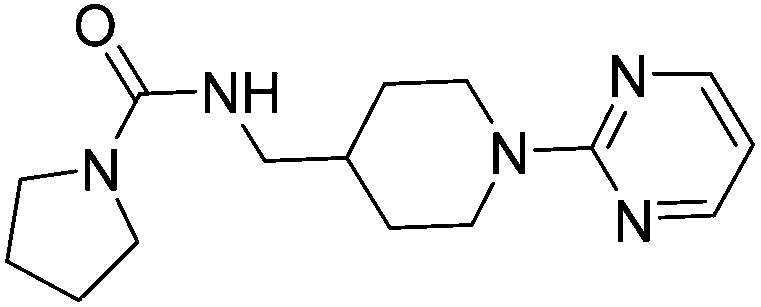	+3.7	20	12

Substituting the 4-(methylamino)methyl functionality on the aromatic ring of compound **5** for a nitrile group to give molecule **15**, resulted in a five-fold decrease in *K*_D_ by ITC and a significant 10-fold improvement using the SPR functional assay (*K*_D_ = 1 μM by ITC and IC_50_ = 3 μM by SPR). Compound **16**, which contains an ethyl ester functionality instead of the nitrile group of **15**, displayed comparable affinity (IC_50_ = 3 μM) by SPR, however binding could not be measured by ITC. Functional activity as measured by SPR was maintained when the amide groups of compounds **15** and **16** were changed to urea in derivatives **17** (IC_50_ = 4 μM) and **18** (IC_50_ = 2 μM) respectively. The substitution of the pyrrolidine ring of ureas **17** and **18** with a cyclopentane ring was however detrimental to the affinity of the resulting compounds **19** (IC_50_ = 25 μM) and **20** (IC_50_ > 100 μM) respectively.

**Fig. 6:**
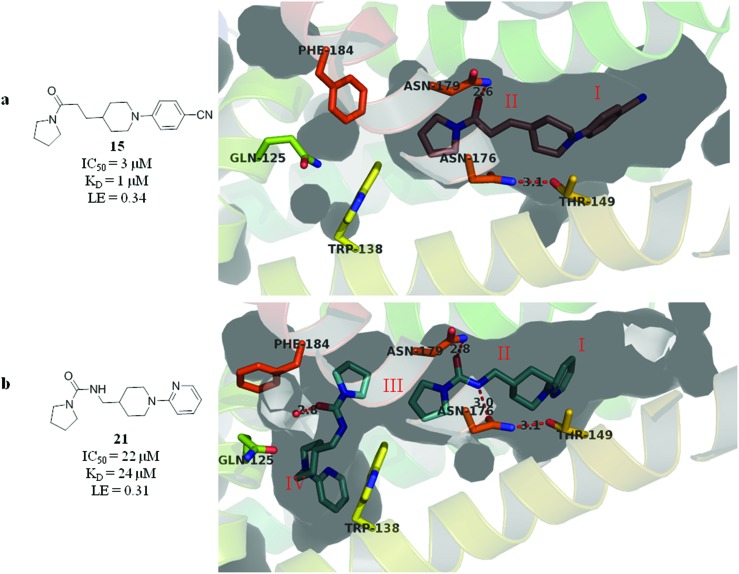
X-ray crystal structures of ligands **15** and **21** respectively bound to EthR. IC_50_ (SPR) and Δ*T*_m_ (DSF) and LE values (based on IC_50_ by SPR) for the two compounds are also shown. (PDB codes ; 5F0F and ; 5EZH).

The compounds **15**, **21** and **22** were successfully soaked into crystals of EthR. The X-ray crystal structures of nitrile **15** and urea **21** are shown in Fig. 6a and b respectively. The structure of compound **22** bound to EthR is given in the ESI (Fig. S2 and S3†).

Compound **5** and its derivatives **15**, **21** and **22** all span sub-pockets I and II of the EthR binding cavity adopting analogous binding positions. In contrast to ligands **5** and **15**, which bind to EthR in a 1 : 1 stoichiometry, two molecules of **21** or **22** soak into the binding cavity of a single EthR protomer (see Fig. 6b and ESI, Fig. S3,† respectively). Ligands **21** and **22** were shown by X-ray crystallography to bind to EthR in an analogous way to each other and are directly superimposable. The second units of both **21** and **22** bind in the region of sub-pockets III and IV, which are fully exposed in the conformations adopted by residues Phe184, Gln125 and Trp138. The carbonyl oxygen atoms of the ligands in this binding mode are stabilised by a hydrogen-bonding interaction with an interstitial water molecule. The pyridyl ring of **21** and the pyrimidyl ring of **22** bound to sub-pocket IV of EthR probe deeper into the binding cavity than the parent fragment **1**.

### Fragment merging strategy II

**Fig. 7:**
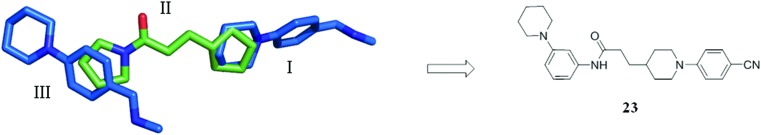
The merging of a molecule of **1** from sub-pocket II (green) surrounded by two molecules of **2** (blue) gives rise to compound **23**.

Finally, to explore whether molecules spanning an even larger volume of the EthR binding pocket might display further improvement in their binding affinity towards EthR, fragment **1** occupying sub-pocket II of EthR was merged with two molecules of **2** from sub-pockets I and III, as shown in Fig. 7. In view of the potency of nitrile **15**, the 4-(methylamino)methyl functionality of fragment **2** from sub-pocket I was simplified to a nitrile group for the design and synthesis of compound **23**.

**Fig. 8:**
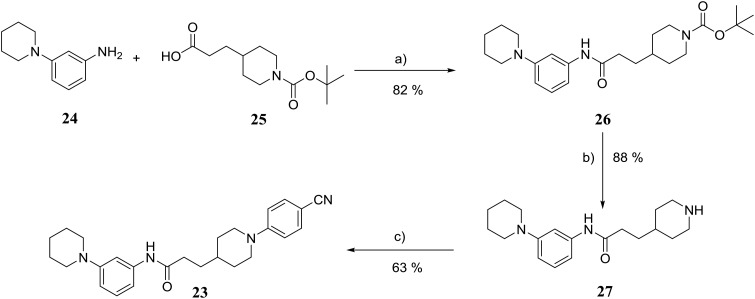
Synthetic scheme for the preparation of 3-(1-(4-cyanophenyl)piperidin-4-yl)-*N*-(3-(piperidin-1-yl)phenyl)propanamide (**23**). (a) DCM, DIPEA, COMU, 22 °C; (b) TFA, DCM, 22 °C; 2 h; (c) 4-fluorobenzonitrile, K_2_CO_3_, anhydrous DMSO, 100 °C; 3 h.

The synthesis of compound **23** is shown in Fig. 8. Coupling of 3-(piperidin-1-yl)aniline **24** with carboxylic acid **25** using COMU gave amide **26** in 82% yield.^20^ Removal of the Boc protection of **26** followed by nucleophilic aromatic substitution^22^ with 4-fluorobenzonitrile afforded the target molecule **23** in 45% yield over three steps.

Two additional derivatives of compound **23**, compounds **28** and **29** (Table 2), were also made (see ESI and Fig. S14 and S15† respectively). The three compounds, **23**, **28** and **29**, were screened against EthR using the fluorescent-based thermal shift assay and the SPR functional assay (Table 2). Compounds **23**, **28** and **29** all gave high positive thermal shift values with EthR (+8.3 °C, +8.7 °C and +9.2 °C respectively) when screened at a concentration of 100 μM. These values provide compelling evidence for the stabilisation imparted by these molecules to EthR under the elevated temperature conditions of the thermal shift assay. Nevertheless, the binding of compounds **23**, **28** and **29** to EthR could not be observed by ITC. These three ligands are less soluble and show significantly decreased ability to disrupt the interaction between EthR and its DNA operator as shown by their IC_50_ values determined by SPR compared to compounds **15**, **16**, **17** and **18**.

**Table 2:** A summary of fluorescent-based thermal shift values against EthR (at 100 μM ligand concentration) and IC_50_ values determined by SPR for compounds **23**, **28** and **29**

Compound number	Compound structure	Δ*T*_m_/°C (100 μM)	IC_50_/μM (SPR)
**23**	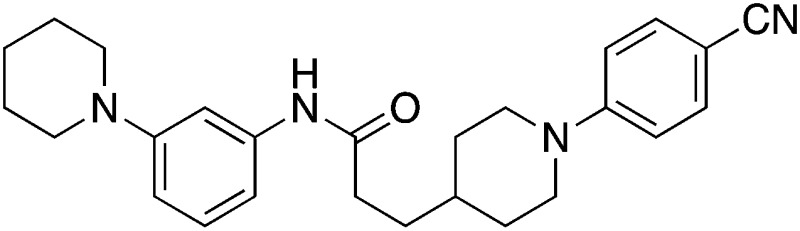	+8.7	>100
**28**	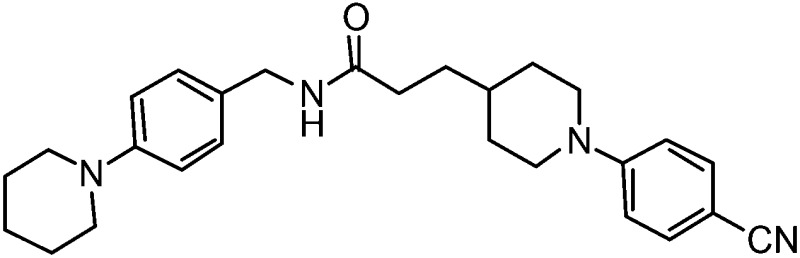	+8.3	33
**29**	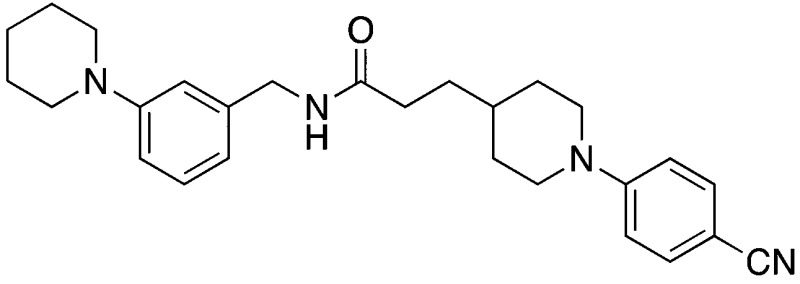	+9.2	52

**Fig. 9:**
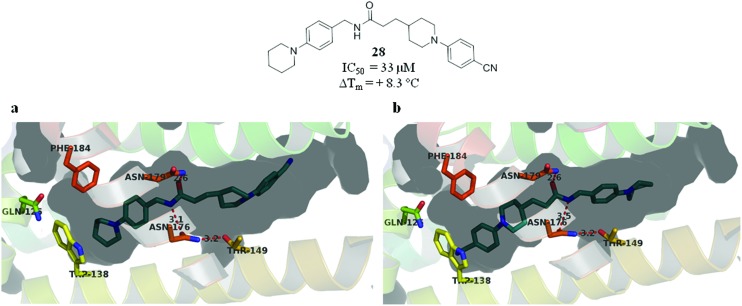
(a and b) X-ray crystal structure of **28** bound to EthR in two different orientations. (PDB code ; 5F0H).

An X-ray crystal structure of ligand **28** bound to EthR was solved to 2.0 Å resolution (see Fig. 9a and b). This structure is of particular interest since electron density was observed corresponding to **28** bound in two different orientations. Furthermore, in both binding modes compound **28** does not fill the EthR binding cavity in the way observed for other ligands previously studied. The usual shape of the EthR binding pocket (see ESI, Fig. S4†) is compromised as a result of the binding of ligand **28**. Despite the conformations of the side chains of residues Phe184 and Trp138 precluding the formation of sub-pockets III and IV in the sense of Fig. S3,† compound **28** still bound to EthR by altering the shape of the hydrophobic cavity and moulding it around its own scaffold (see ESI, Fig. S5†).

In order to see whether the SPR results translate into effective levels of ethionamide boosting, all the merged compounds were tested for their ability to boost ethionamide activity in *M. tuberculosis* infected macrophages as described previously.^13,14^ None of compounds **3–5**, **14–23**, **28** and **29** showed any ethionamide boosting in macrophages. The lack of efficacy is probably compounded by poor permeability across the mycobacterial envelope and/ or host cell membrane. The ability of **1** to boost ethionamide, which we have reported previously,^18^ possibly arises due to its small size and ability to penetrate the *M. tuberculosis* bacillus.

## Conclusions

We have previously identified two fragment molecules, **1** and **2**, each binding twice to EthR, which together fill the entire hydrophobic cavity. Examination of the X-ray crystal structures of these fragments gave three possible combinations of merging two adjacent fragment units. These merged compounds **3**, **4** and **5** were synthesised and soaked into preformed crystals of EthR. X-ray crystallography showed that compounds **4** and **5** recapitulated the binding mode of the original fragment hits **1** and **2**.

Compounds **14–22**, synthesised to explore the SAR around merged compound **5**, resulted in compounds capable of inhibiting the interaction between EthR and its DNA operator with IC_50_ values in the range 2–4 μM, representing valuable new molecular probes for the EthR system. Subsequent further strategies to merge fragment **1** with two molecules of fragment **2** within the EthR binding cavity were also explored. Although this approach resulted in compounds exhibiting high positive thermal shifts with EthR, these ligands were not as effective at disrupting the interaction between the transcriptional repressor and its DNA operator as the most potent compounds **15**, **16**, **17** and **18**.

Our fragment merging strategy and the subsequent SAR work around compound **5** proved fruitful in providing inhibitors capable of disrupting the interaction between EthR and its DNA operator with IC_50_ values in the single-digit micromolar range as shown by SPR. However, none of the merged compounds were capable of boosting ethionamide activity in *M. tuberculosis* infected macrophages, presumably due to inability to permeate the mycobacterial cell envelope.
