# Supplementary material for: A fragment merging approach towards the development of small molecule inhibitors of Mycobacterium tuberculosis EthR for use as ethionamide boosters
Source: Org Biomol Chem. 2016 Jan 25;14(7):2318–26. doi: 10.1039/c5ob02630j (PMC4759522; doi:10.1039/c5ob02630j)
Supplement: Supplementary file 1 [file OB-014-C5OB02630J-s001.pdf]

Electronic Supplementary Material (ESI) for Organic & Biomolecular Chemistry.
This journal is © The Royal Society of Chemistry 2016

A fragment merging approach towards the development of small molecule inhibitors of *Mycobacterium tuberculosis* EthR for use as ethionamide boosters

Supplementary Information

Petar O. Nikiforov,[a] Sachin Surade,[b] Michal Blaszczyk,[b] Vincent Delorme,[c] Priscille Brodin,[c] Alain R. Baulard,[c] Tom L. Blundell,[b] Chris Abell[a*]

[a] Department of Chemistry, University of Cambridge, Lensfield Road, Cambridge, CB2 1EW, UK

[b] Department of Biochemistry, University of Cambridge, 80 Tennis Court Road, Cambridge, CB2 1GA, UK

[c] Inserm U1019 – CNRS UMR 8204, Institut Pasteur de Lille, Université de Lille, 1 rue du Professeur Calmette, 59019, Lille, France

Table of Contents

Molecular Biology 3

X- ray crystallography 3

Figure S1 X-ray crystallography data collection and final refinement statistics 4

Figure S2 X-ray crystal structure showing a full view of an EthR monomer with two molecules of ligand **22** bound to it 5

Figure S3 X-ray crystal structure of ligand **22** bound to EthR 5

Figure S4 The usual shape of the EthR pocket consisting of sub-pockets I, II, III and IV as defined by the binding of fragments **1** and **2** 6

Figure S5 X-ray crystal structure of **28** bound to EthR 7

Biophysical assays 7

Differential scanning fluorimetry (DSF) 7

Isothermal titration calorimetry (ITC) 7

Figure S6 ITC trace for compound **15** 8

Surface plasmon resonance (SPR) 9

Figure S7 IC_{50} curve (SPR) for compound **15** 9

<u>**Chemistry**</u> 10

General Information 10

Synthetic Schemes 11

General procedure A 14

General procedure B 15

Synthesis 15

Selected Spectra 41

^{1}H NMR spectra 41

^{13}C NMR spectra 55

Example LCMS spectrum 68

<u>**References**</u> 69

Molecular Biology

The EthR gene was cloned into a pHAT5 vector[1] using BamHI and EcoRI restriction sites. *Escherichia coli* BL21 (DE3) (Novagen) strain was used for EthR expression. Fresh overnight starting culture (25 mL) grown in LB media overnight at 37 °C, 220 rpm was added to LB media (1L) and incubated at 37°C until the colony reached OD_{600} ~0.8. The culture was the induced with IPTG (1mM) and incubated at 37 °C, 220 rpm, for an additional 3 h. The cells were harvested by centrifugation (4200 *g* for 20 min at 4 °C) and re-suspended in lysis buffer [50 mM Hepes (pH 7.5) and 150 mM NaCl; 25 mL] supplemented with EDTA-free complete protease inhibitor cocktail (Roche). The cells were lysed by sonication (10 pulses of 30 s each) and debris was removed by centrifugation (35000 *g* for 1 h at 4 °C). The supernatant was loaded onto a 5 mL HiTrap IMAC Fast Flow column (GE Healthcare) charged with Ni^{2+} at 5ml/min rate and washed with buffer (50 mM Hepes pH 7.5, 150 mM NaCl and 20 mM imidazole; 50 mL). After elution with elution buffer (50 mM Hepes pH 7.5, 150 mM NaCl and 250 mM imidazole), the protein was further purified by size exclusion chromatography (Superdex 200) and concentrated (4200 *g* at 4 °C) using 10 kDa Amicon® Ultra concentrators.

X-ray crystallography

Crystallisation of EthR was performed using the sitting-drop vapour diffusion method at 25 °C. A drop consisted of 1.0µL of reservoir solution (1.7–2.1M ammonium sulphate, 0.1M MES-Na (pH 6–7), 5-15% (v/v) glycerol and 7–12% (v/v) 1,4-dioxane) and 0.5–1.0µL of protein solution (20mg/ml EthR, 0.5M NaCl, 15mM Tris/HCl pH 8.0 and 10% (v/v) glycerol).[2] Compounds (100mM in DMSO) were mixed with mother liquor (1.9M ammonium sulphate, 0.1M MES-Na pH 6.5 and 12.5% (v/v) glycerol) to a final concentration of 1–10mM. EthR crystals were washed free from 1,4-dioxane by placing them in 1,4-dioxane free mother liquor for a few hours. The washed EthR crystals were then transferred to the fragment-containing solutions and incubated for 1–16 h. Crystals were cryoprotected by passing them briefly through mother liquor containing 20% (v/v) of ethylene glycol and then flash-frozen in liquid nitrogen. X-ray crystallographic datasets were collected at the European Synchrotron Radiation Facility (Grenoble, France) and at the Diamond Light Source (Harwell, UK). X-ray datasets were indexed and integrated using autoPROC[3], XDS[4] and Mosfilm[5]. The scaling of datasets was carried out using SCALA/AIMLESS software.[6] Structures were solved using the molecular replacement method with PHASER[7] (PDB ID 1T56 was used as search probe). Structures were further refined with Refmac5[8] (part of CCP4[9] suite), PHENIX[10] or BUSTER[11] to satisfactory level of R/Rfree using maximum-likelihood restrained refinement. Ligand restrain files were prepared by Dundee PRODRG2 server,[12] libcheck[13] or PHENIX elbow software. Every structure was modelled manually in Coot[14] (including ligand and essential water molecules). Images of X-ray crystal structures in figures were prepared using PyMOL (http://www.pymol.org).

CMPD#	**01**	**02**	**03**	**04**	**05**	**14**	**15**	**21**	**22**	**28**
PDB ID	5F1J	5F27	5F04	5F0C	5EYR	5F08	5F0F	5EZH	5EZG	5F0H
**Data collection***										
Space group	$P4_12_12$	$P4_12_12$	$P4_12_12$	$P4_12_12$	$P4_12_12$	$P4_12_12$	$P4_12_12$	$P4_12_12$	$P4_12_12$	$P4_12_12$
Cell parameters:										
a [Å]	121.81	120.17	121.80	121.70	120.89	120.70	121.30	121.40	121.50	120.30
b [Å]	121.81	120.17	121.80	121.70	120.89	120.70	121.30	121.40	121.50	120.30
c [Å]	33.73	33.63	33.84	33.78	33.83	33.74	33.83	33.89	33.84	33.7
$\alpha=\beta=\gamma=90°$										
Resolution range [Å]	43.07 – 1.63	42.49 – 1.68	86.12 – 1.84	54.44 – 1.87	85.48 – 1.57	53.97 – 1.92	54.24 – 1.76	85.85 – 1.70	85.91 – 1.84	53.82 – 1.99
	(1.67 – 1.63)	(1.78 – 1.68)	(1.89 – 1.84)	(1.92 – 1.87)	(1.72 – 1.57)	(1.97 – 1.92)	(1.81 – 1.76)	(1.74 – 1.70)	(1.89 – 1.84)	(2.04 – 1.99)
No. of observations										
total	424257	238221	286725	273524	463192	247645	325220	359919	289734	223127
unique	(20912)	(32598)	(22201)	(20632)	(109019)	(18789)	(24227)	(26001)	(22022)	(15859)
	32327	27668	22766	21522	34875	19677	25717	28584	22588	17617
	(2352)	(3938)	(1665)	(1530)	(7899)	(1421)	(1860)	(2056)	(1632)	(1249)
R_{merge}	0.065(0.700)	0.076 (0.286)	0.053 (0.880)	0.069 (0.845)	0.050 (0.963)	0.066 (0759)	0.054 (0.884)	0.049 (0.788)	0.089 (0.787)	0.063 (0.688)
$I/\sigma(I)$	23.0 (2.9)	18.3 (7.0)	27.9 (4.1)	25.4 (3.8)	29.6 (2.8)	23.2 (3.9)	29.5 (4.1)	28.4 (3.9)	21.1 (4.6)	25.7 (4.3)
Completeness [%]	99.8 (99.8)	97.3 (97.2)	99.8 (99.2)	99.4 (98.9)	97.8 (94.5)	99.8 (99.8)	99.8 (99.3)	100.0 (99.9)	99.7 (98.5)	99.9 (99.6)
Multiplicity	13.1 (8.9)	8.6 (8.3)	12.6 (13.3)	12.7 (13.5)	13.3 (13.8)	12.6 (13.2)	12.6 (13.0)	12.6 (12.6)	12.8 (13.5)	12.7 (12.7)
**Refinement**										
Refinement program	REFMAC	PHENIX	REFMAC	REFMAC	BUSTER	REFMAC	REFMAC	REFMAC	REFMAC	REFMAC
Resolution [Å]	33.78 – 1.63	41.49 – 1.68	54.47 – 1.84	54.44 – 1.87	21.44 – 1.57	38.16 – 1.92	54.24 – 1.76	60.71 – 1.70	85.91 – 1.84	53.82 – 1.99
No. reflections	32230	27637	22724	21485	34835	19632	25670	28536	22554	17575
R_{work}/R_{free} [%]	19.7/22.8	19.9/22.8	19.2/22.0	19.6/23.4	18.6/20.3	19.8/23.3	20.0/23.5	19.4/23.2	18.3/20.8	19.8/23.0
RMS deviations										
Bonds [Å]	0.030	0.006	0.021	0.025	0.010	0.022	0.025	00.24	0.025	0.022
Angles [°]	2.27	0.84	2.11	2.11	0.86	2.01	2.31	2.35	2.29	2.08
Ramachandran										
Favoured [%]	98	99	99	98	99	97	98	99	98	97
Outliers [%]	1	0	0	1	0	0	0	0	0	1

* Parameters shown in brackets are for the highest resolution shell

Figure S1 X-ray crystallography data collection and final refinement statistics

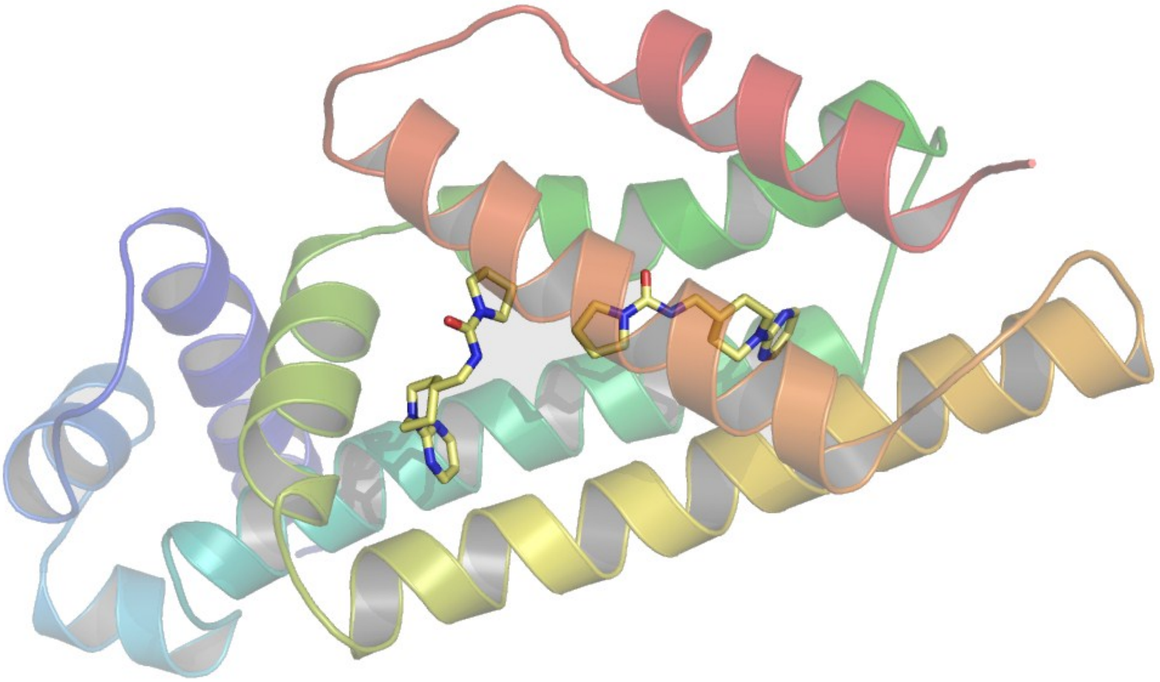

Figure S2 X-ray crystal structure showing a full view of an EthR monomer with two molecules of ligand **22** bound to it. (PDB code 5EZG)

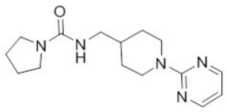

22
IC_{50} = 20 μM
LE = 0.31

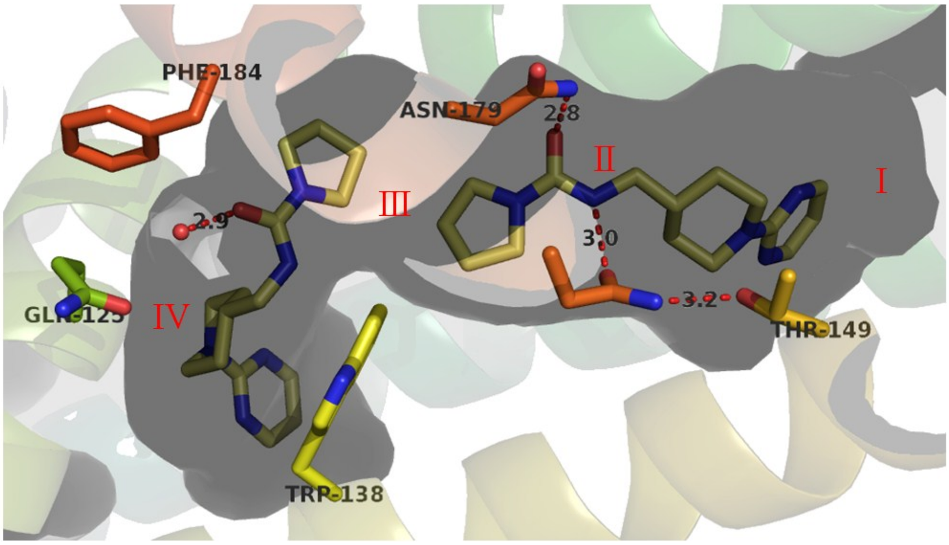

Figure S3 X-ray crystal structure of ligand **22** bound to EthR. One molecule of **22** spans sub-pockets I and II, while a second molecule of **22** occupies sub-pockets III and IV of the EthR binding cavity. (PDB code 5EZG)

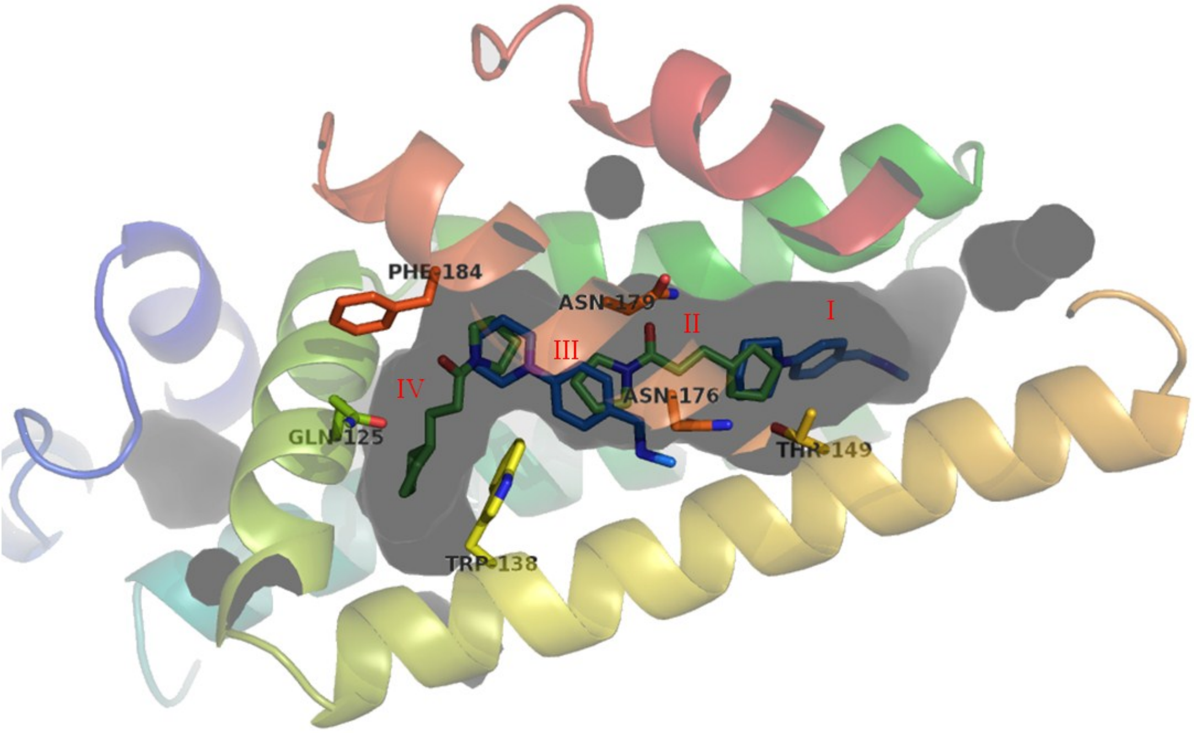

Figure S4 Usual shape of the EthR pocket consisting of sub-pockets I, II, III and IV as defined by the binding of fragments **1** and **2** (crystal structures of the two fragments are overlaid on top of each other). (PDB codes 5F1J and 5F27)

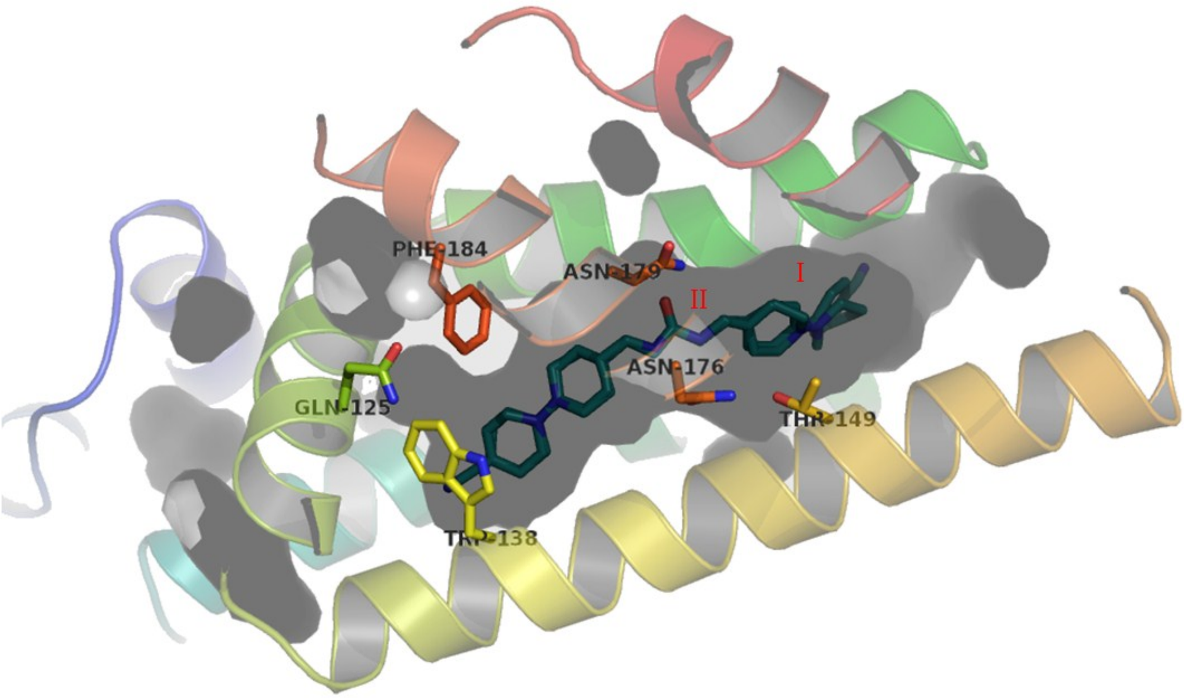

Figure S5 Crystal structure of **28** bound to EthR (the two different binding modes for this ligand are super-imposed). Although the conformations of the side chains of residues Phe184 and Trp138 preclude the formation of sub-pockets III and IV in the sense of Figure S3, compound **28** is still capable of binding to the protein by altering the shape of the EthR hydrophobic cavity and moulding it around its own scaffold. (PDB code 5F0H)

Biophysical assays

Differential scanning fluorimetry (DSF)

DSF measurements were carried out using a Bio-Rad CFX Connect machine with a 96-well reaction module. Samples (50 µl each) containing EthR (20 µM), NaCl (150 mM), Tris.HCl (20 mM, pH = 8.5), SYPRO orange (2.5x) and test compound (100 µM) in water were prepared in 96-well plates. The 96-well plates were heated linearly from 25 °C to 95 °C using a temperature increment of 0.5 °C every 30 seconds. Melting curves represent plots of the fluorescence emission intensity at λ_{max} 490/575 nm of each sample against temperature. Melting temperatures (T_m) were determined as the temperatures at which the minima on the negative first derivatives of the melting curves occurred.

Isothermal titration calorimetry (ITC)

An aqueous solution of the fragment to be tested (1 mM) was prepared containing NaCl (300 mM), Tris.HCl (20 mM, pH = 8.0), d_6-DMSO (10% v/v) and glycerol (to match the 10% v/v glycerol content in the EthR stock solution). A separate aqueous solution of EthR (50 µM)

containing NaCl (300 mM), Tris.HCl (20 mM, pH = 8.0) and DMSO-d_6 (10% v/v) was prepared and placed in the sample cell of a MicroCal iTC$_{200}$ microcalorimeter (GE Healthcare). The fragment solution was then titrated to the EthR solution over 39 injections (first injection of 0.4 μl and subsequent injections of 1.0 μl). Data was fitted to a one site binding model using Origin software.

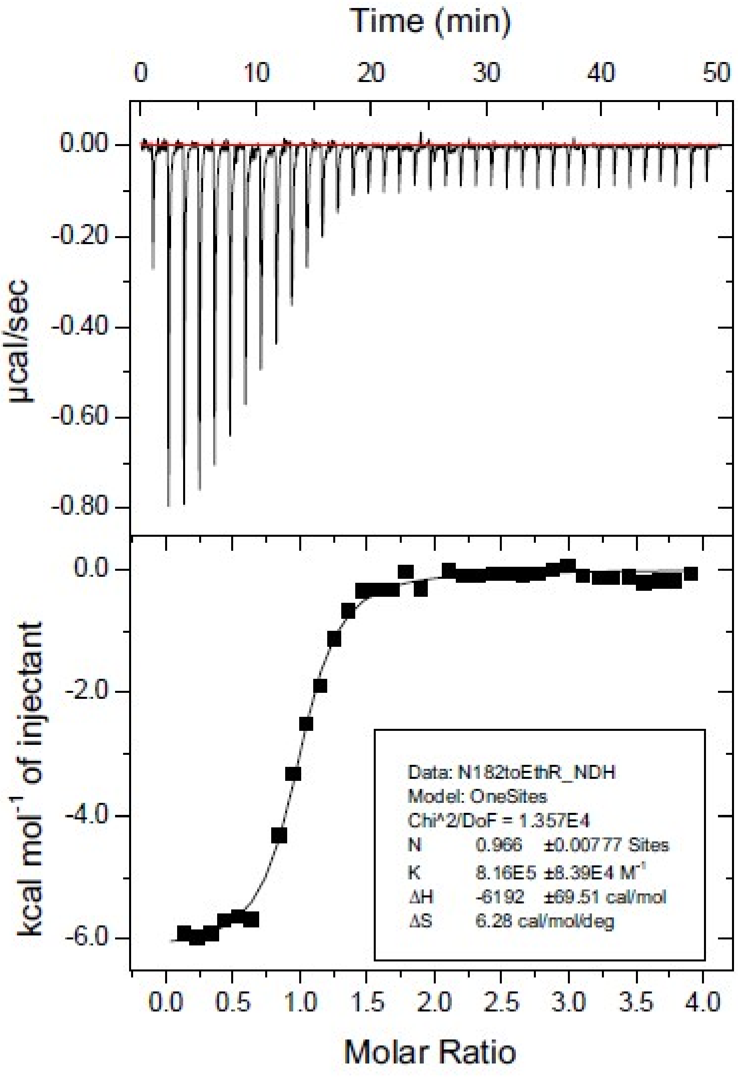

Figure S6 ITC trace for 4-(4-(3-Oxo-3-(pyrrolidin-1-yl)propyl)piperidin-1-yl)benzonitrile **15**

Surface plasmon resonance (SPR)

The SPR assay was carried out using a BIAcore T100 instrument as described previously.[2] The assay was designed to measure the interaction of EthR with the *ethA* promoter DNA sequence (106 bp – experimental DNA), immobilised via biotin-streptavidin linkage onto an

SA Series S Sensor Chip (BIAcore). DNA from pUC19 (113 bp) was used as the control against non-specific binding. The experimental (106 bp) and control (113 bp) DNA fragments were produced as described previously.[15,16] Biotinylated control and experimental DNA were immobilized to the chip surface to achieve stable fixation levels of 247 and 252 resonance units (RU) respectively.

For screening, EthR/ fragment solution (1-2 μM EthR and required concentration of fragment made up in running buffer (2 mM $MgCl_2$, 10 mM Tris-Cl pH 7.5, 0.1 mM EDTA, 200 mM NaCl, 2% (v/v) DMSO) was flowed over the chip at 20 μL/min for 180 s. The dissociation time was 180 s. To determine binding levels, the response of the control channel at steady state was subtracted from that of the experiment channel. The chip was regenerated between samples using 0.03% w/v SDS in running buffer (passed at a flow rate of 20 μL/ min for 60 s).

For IC_{50} calculations, the SPR response of EthR binding to the immobilized DNA was measured at various concentrations of compound. The resulting RUs were used to fit the data using nonlinear regression with variable slope dose-response inhibition constrains on GrapfPad PRISM 5.00. IC_{50} values were calculated as the compound concentrations necessary to inhibit 50% of the maximal interaction between EthR and its DNA operator sequence.

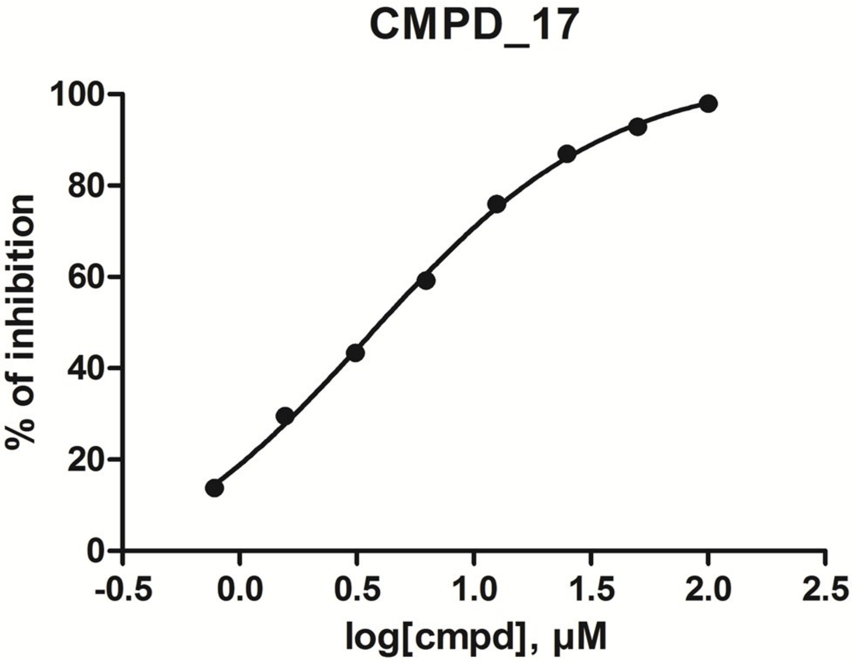

Figure S7 IC_{50} curve (SPR) for *N*-((1-(4-Cyanophenyl)piperidin-4-yl)methyl)pyrrolidine-1-carboxamide **17**

Chemistry

General Information

^{1}H NMR and ^{13}C NMR spectra were recorded using Bruker DPX-400 or Bruker DPX-500 NMR spectrometers. Chemical shifts are given in parts per million (ppm). All ^{13}C NMR spectra are proton decoupled. For molecules, in which restricted amide rotation gives rise to multiple signals per nucleus, all signals observed are reported in the ^{1}H NMR and ^{13}C NMR spectra and the appropriate ratios of peaks in the ^{1}H NMR spectra reflect the ratios of different rotamers, Compounds **3**, **11**, **12** and **13** were shown to be rotamers in the 1H NMR. Coupling constants are reported in Hz where interpretable and the conventional abbreviations for assigning peak multiplicity are used as follows: s = singlet, d = doublet, t = triplet, m = multiplet, br = broad.

High resolution mass spectrometry (HRMS) was performed using a Waters LCT Premier high-resolution spectrometer in electrospray ionisation (ESI) mode.

LCMS spectra were recorded using a Waters HClass UPLC system coupled to a Waters single quad detector eluting at a constant flow rate of 0.8 ml/ min using a constant gradient of 5 – 100% acetonitrile in 0.1% v/v aqueous formic acid.

Infrared spectrometry was performed using a Perkin-Elmer One FTIR Spectrometer with attenuated transmittance reflectance (ATR). The abbreviations (w) and (br) have been used to describe weak and broad IR absorbances respectively.

All commercially available reagents were used as purchased without further purification. All organic solvents used were either freshly distilled or purchased as anhydrous. Purification of intermediates and final compounds was carried out by automated flash column chromatography using Biotage SNAP Kp-Sil pre-packed columns run on either Biotage Isolera One or Biotage Isolera Four instruments.

Microwave reactions were performed using a Biotage Initiator system under reaction conditions as indicated for each individual reaction.

Following aqueous work-up, organic solutions of intermediates and final compounds were dried using Isolute ® phase separators from Biotage (referred to as hydrophobic frits).

The purity of the compounds was measured by LC-MS with UV-Vis detection and all compounds were of a purity of > 95% unless otherwise stated.

Synthetic Schemes

Figure S8 Synthesis of 3-(1-(4-((methylamino)methyl)phenyl)piperidin-4-yl)-1-(pyrrolidin-1-yl)propan-1-one (**5**)

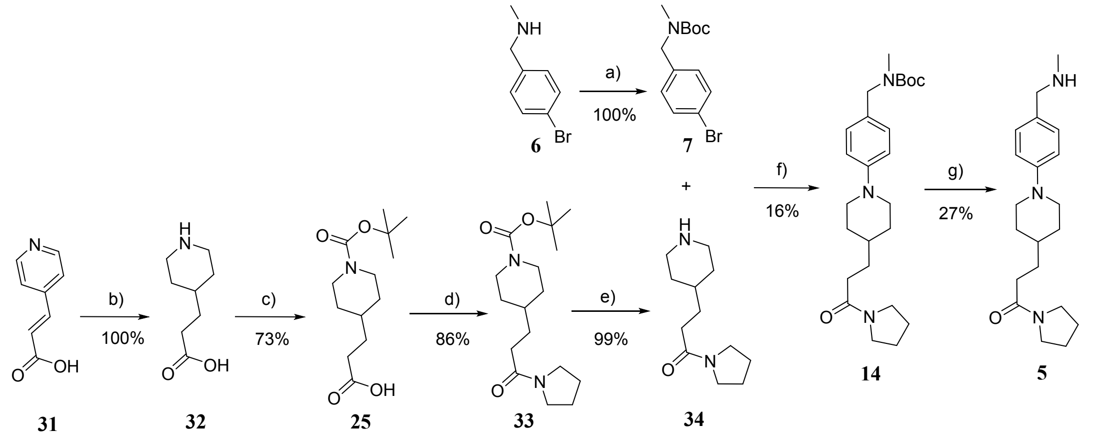

a) di-*tert*-butyl dicarbonate, THF; 22 °C; b) water, 35 % wt aqueous ammonia (pH = 6 – 7), Rh on activated alumina, H_2 (8 bar); 95 °C; 48 h; c) K_2CO_3, H_2O, di-*tert*-butyl dicarbonate, NEt_3 THF; 0 → 22 °C; 4 h; d) pyrrolidine, COMU, diisopropylethylamine, DCM; 22 °C; e) DCM, TFA, 2 h; then DCM, K_2CO_3, 60 h; f) $Pd(OAc)_2$, KO^{t}Bu, 2-(di-*tert*-butylphosphino) biphenyl, toluene; 100 °C, 4 h; g) 4.0 M HCl in dioxane; 15 min.

Figure S9: Synthesis of 4-(4-(3-oxo-3-(pyrrolidin-1-yl)propyl)piperidin-1-yl)benzonitrile (**15**) and Ethyl 4-(4-(3-oxo-3-(pyrrolidin-1-yl)propyl)piperidin-1-yl)benzoate (**16**)

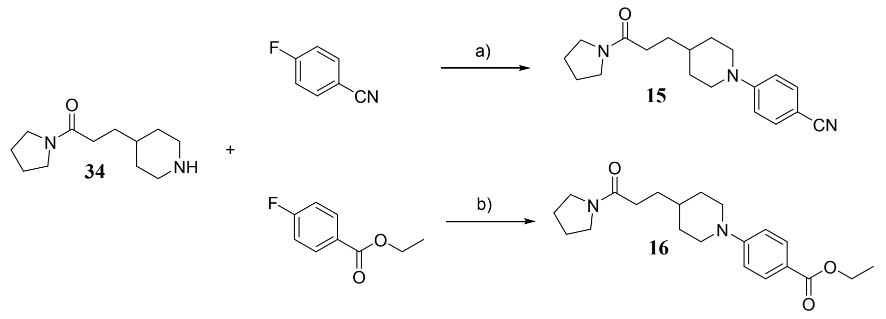

a) K_2CO_3, DMSO, 90 °C, 16 h; b) K_2CO_3, DMSO, 90 °C, 3 h.

Figure S10: Synthesis of 3-methyl-1-(pyrrolidine-1-carbonyl)-1*H*-imidazol-3-ium iodide (**36**)

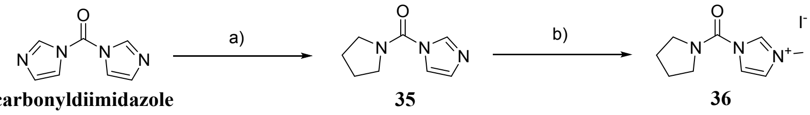

a) pyrrolidine, carbonyldiimidazole, THF, 22 °C; b) MeI, DCM, 22 °C.

Figure S11: Synthesis of compounds **17, 18, 19** and **20**

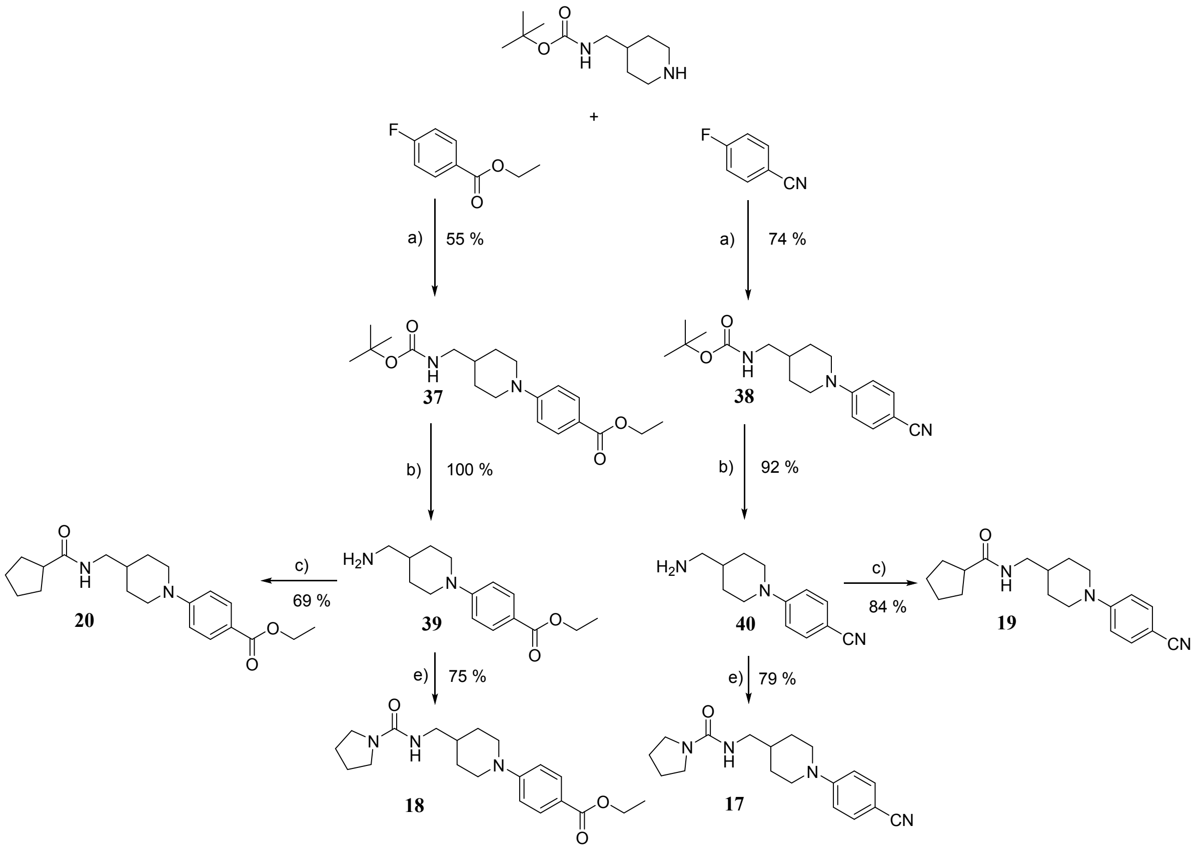

a) K_2CO_3, DMSO, 90 °C; b) TFA, DCM, 22 °C; c) cyclopentaneacetic acid, DCM, diisopropylethylamine, COMU, 22 °C; d) THF, H_2O, LiOH, 75 °C; e) 3-methyl-1-(pyrrolidine-1-carbonyl)-1H-imidazol-3-ium iodide, NEt_3, DCM, 22 °C; 18 h.

Figure S12: Synthesis of compounds **21** and **22**

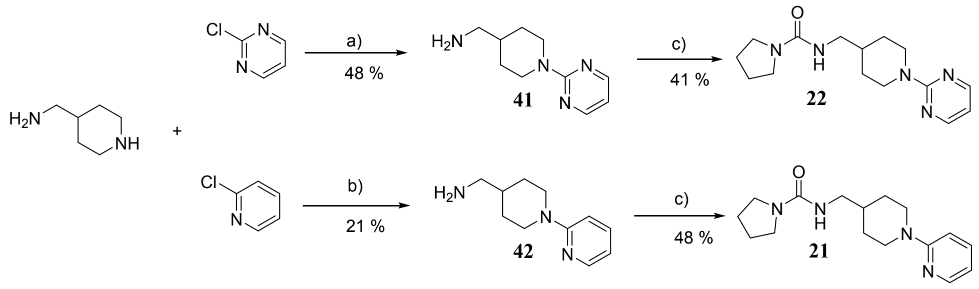

a) i) 4-(aminomethyl) piperidine, toluene, acetophenone, *p*-toluenesulfonic acid; Dean-Stark; 5 h; ii) 2-chloropyrimidine, NEt_3, 95 °C; 18 h; iii) 5 M HCl; b) 4-(aminomethyl) piperidine, 1-pentanol, Na_2CO_3, 2-chloropyridine, 130 °C; 24 h; c) 3-methyl-1-(pyrrolidine-1-carbonyl)-1H-imidazol-3-ium iodide, NEt_3, DCM, 22 °C; 3 h.

Figure S13: Synthesis of 3-(1-(4-cyanophenyl)piperidin-4-yl)-N-(3-(piperidin-1-yl)phenyl)propanamide (**23**)

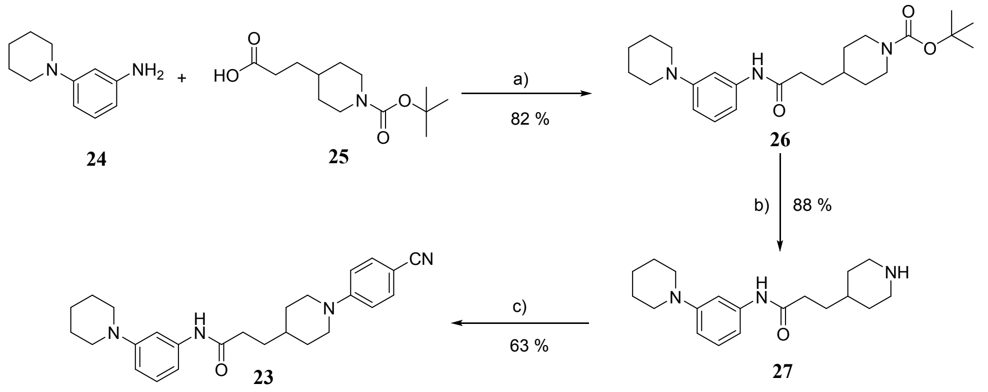

a) DCM, diisopropylethylamine, COMU, 22 °C; b) TFA, DCM, 22 °C; 2 h; c) 4-fluorobenzonitrile, K_2CO_3, anhydrous DMSO, 100 °C; 3 h.

Figure S14: Synthesis of 3-(1-(4-cyanophenyl)piperidin-4-yl)-N-(3-(piperidin-1-yl)benzyl)propanamide (**28**)

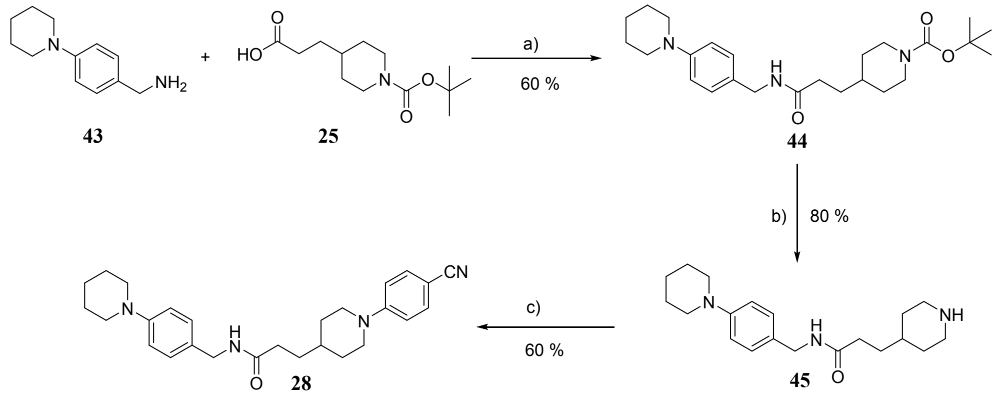

a) DCM, diisopropylethylamine, COMU, 22 °C; b) TFA, DCM, 22 °C; 2 h; c) 4-fluorobenzonitrile, K_2CO_3, anhydrous DMSO, 100 °C; 3 h.

Figure S15: Synthesis of 3-(1-(4-Cyanophenyl)piperidin-4-yl)-N-(4-(piperidin-1-yl)benzyl)propanamide (**29**)

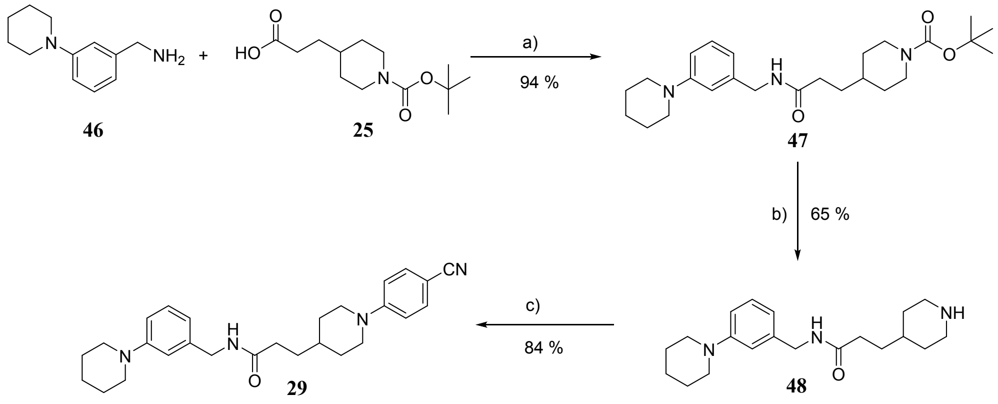

a) DCM, diisopropylethylamine, COMU, 22 °C; b) TFA, DCM, 22 °C; 2 h; c) 4-fluorobenzonitrile, K_2CO_3, anhydrous DMSO, 100 °C; 3 h.

General procedure A [17]

Amine (1 eq.), carboxylic acid (1 eq.) and diisopropylethylamine (5 eq.) were dissolved in anhydrous DCM (2 mL). COMU (1.1 eq.) was added and the reaction mixture was stirred at room temperature for 16 – 24 h. The solvent was evaporated *in vacuo*. The residue was

dissolved in EtOAc (10 mL) and washed with water (2 x 10 mL). The organic layer was concentrated *in vacuo* and the crude material was purified by flash column chromatography.

General procedure B (TFA deprotection) [18]

The Boc-protected amine was dissolved in DCM (6 mL). TFA (3 mL) was added and the reaction mixture was stirred at room temperature for 1 - 5 h. The solvent was evaporated *in vacuo*. The crude material was dissolved in DCM (10 mL) and washed with saturated aqueous sodium bicarbonate. The organic extracts were dried using a hydrophobic frit and evaporated *in vacuo*.

Synthesis

3-Cyclopentyl-1-(3-(4-((methylamino)methyl)phenyl)tetrahydropyrimidin-1(2*H*)-yl)propan-1-one (3)

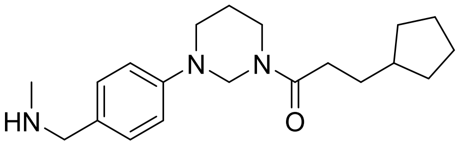

tert-butyl-(4-(3-(3-cyclopentylpropanoyl)tetrahydropyrimidin-1(2*H*)-yl)benzyl)(methyl) carbamate (**13**) (11.2 mg, 26 µmol) was dissolved in anhydrous DCM (0.67 mL) and TFA (0.33 mL) was added. The reaction mixture was stirred at room temperature for 2 h, after which the solvents were removed *in vacuo*. The residue was re-dissolved in DCM (5 mL) and washed with saturated aqueous $NaHCO_3$ (2 mL). The DCM layer was separated and dried by passing through a hydrophobic frit. The solvent was evaporated *in vacuo* and the residue was purified by automated flash chromatography (0 – 10% 2M methanolic ammonia in DCM). *Amine* **3** was isolated as colourless gum (5.3 mg, 62%); ^{1}H NMR (400 MHz, $CDCl_3$) δ 7.27 – 7.23 (m, 2H), 7.02 – 6.97 (m, 1.2H), 6.95 – 6.91 (m, 0.6H), 5.00 (s, 1.2H), 4.77 (s, 0.6H), 3.75 (s, 2H), 3.73 – 3.70 (m, 0.6H), 3.67 – 3.59 (m, 1.2H), 3.59 – 3.50 (m, 1.2H), 3.44 – 3.32 (m, 0.6H), 2.48 (s, 3H), 2.45 – 2.40 (m, 6H), 2.37 – 2.30 (m, 1.2H), 2.01 – 1.70 (m, 7H), 1.70 – 1.47 (m, 5H), 1.11 (m, 2H); ^{13}C NMR (125 MHz, $CDCl_3$) δ 171.8, 148.1, 147.7, 130.6, 129.8, 128.8, 117.3, 116.7, 65.4, 59.6, 54.8, 54.6, 49.2, 49.2, 45.5, 41.7, 39.8, 35.2, 34.8, 32.5, 31.5, 31.3, 25.1, 23.9; IR, ν_{max} (ATR): 3251 (br), 2945, 2866, 1635 cm^{-1}; HRMS (*m/z*):

[M + H]$^+$ calcd for $C_{20}H_{32}N_3O$, 330.2540; found, 330.2525; LCMS (ESI) [M + Na]$^+$ *m/z*: 352.3, retention time = 1.50 min.

3-Cyclopentyl-N-(3-(piperidin-1-yl)phenyl)propanamide (4)

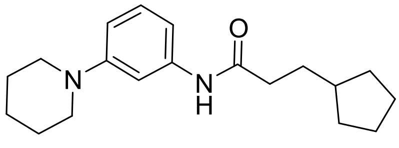

Prepared according to **general method A** using 3-(piperidin-1-yl)aniline **24** (50 mg, 0.28 mmol), 3-cyclopentylpropionic acid (40.5 µL, 0.28 mmol), diisopropylethylamine (247 µL, 1.42 mmol), DCM (2.5 mL) and COMU (134 mg, 0.31 mmol). Purification by flash column chromatography (0 – 33 % EtOAc in pet. ether 40/ 60) afforded the *amide* **4** as a pale pink oil (53 mg, 62 %). TLC (EtOAc/ pet. ether 40/ 60, 1: 2, v/ v), R_f = 0.38; ^{1}H NMR (400 MHz, $CDCl_3$) δ 7.35 (dd, *J* = 2.4, 2.0 Hz, 1H), 7.17 (dd, *J* = 8.3, 7.7 Hz, 1H), 7.11 (s, 1H), 6.81 (dd, *J* = 7.7, 2.0 Hz, 1H), 6.69 (dd, *J* = 8.3, 2.4 Hz, 1H), 3.24 – 3.14 (m, 4H), 2.41 – 2.31 (m, 2H), 2.01 – 1.41 (m, 15H), 1.23 – 1.06 (m, 2H); ^{13}C NMR (125 MHz, $CDCl_3$) δ 171.4, 152.8, 138.8, 129.3, 112.2, 110.1, 107.8, 50.4, 39.7, 37.2, 32.5, 31.8, 25.8, 25.2, 24.3; HRMS (*m/z*): [M + H]$^+$ calcd for $C_{19}H_{29}N_2O$, 301.2280; found, 301.2277; LCMS (ESI) [M + H]$^+$ *m/z*: 301.3, retention time = 1.79 min.

3-(1-(4-((Methylamino)methyl)phenyl)piperidin-4-yl)-1-(pyrrolidin-1-yl)propan-1-one (5)

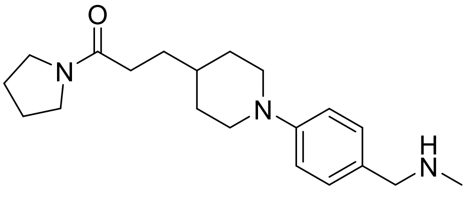

Compound **14** (16 mg, 49 µmol) was dissolved in 4.0 M HCl in 1, 4-dioxane (0.5 mL) and stirred at room temperature for 2 h, after which the solvent were removed *in vacuo*. The residue was re-dissolved in DCM (5.0 mL) and washed with saturated aqueous $NaHCO_3$ (1.0 mL). The organic layer was dried by passing through a hydrophobic frit and the solvent was removed *in vacuo* to give a*mine* **5** as a colourless gum (7.8 mg, 62%); ^{1}H NMR (400 MHz, $CDCl_3$) δ 7.11 (d, *J* = 8.4 Hz, 2H), 6.83 (d, *J* = 8.4 Hz, 2H), 3.58 (s, 4H), 3.45 – 3.28 (m, 6H), 2.64 – 2.55 (m, 2H), 2.37 (s, 2H), 2.30 – 2.16 (m, 2H), 1.95 – 1.83 (m, 2H), 1.84 – 1.68 (m,

4H), 1.65 – 1.55 (m, 3H), 1.39 – 1.28 (m, 2H); IR, ν_{max} (ATR): 3410 (br), 2930, 1675, 1611 cm^{-1}; HRMS (*m/z*): $[M + H]^+$ calcd for $C_{20}H_{32}N_3O$, 330.2540; found, 330.2527; LCMS (ESI) $[M – CH_3NH]^+$ *m/z*: 299.5, retention time = 2.87 min.

***tert*-Butyl 5,6-dihydropyrimidine-1(4*H*)-carboxylate (9)**

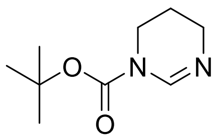

1, 4, 5, 6-tetrahydropyrimidine (200 µl, 2.4 mmol) was dissolved in anhydrous THF (4.0 mL) and triethylamine (0.68 mL, 4.8 mmol) was added. The reaction mixture was stirred and cooled in an ice/ water bath. Di-*tert*-butyl dicarbonate (0.58 g, 2.7 mmol) was added, the ice/ water bath was removed and the reaction mixture was stirred at room temperature for 18 h. The solvent was removed *in vacuo* and the residue was purified by automated flash chromatography (0 – 10% MeOH in DCM) to give *tert*-butyl 5,6-dihydropyrimidine-1(4*H*)-carboxylate (**9**) as colourless liquid (295 mg, 66%); ^1H NMR (400 MHz, $CDCl_3$) δ 8.12 – 7.96 (m, 1H), 3.62 – 3.55 (m, 2H), 3.46 – 3.36 (m, 2H), 1.91 – 1.80 (m, 2H), 1.52 (s, 9H); ^{13}C NMR (125 MHz, $CDCl_3$) δ 151.8, 143.7, 82.8, 43.8, 40.7, 28.0, 20.5; IR, ν_{max} (ATR): 2977, 1714, 1640 cm^{-1}; HRMS (*m/z*): $[M + H]^+$ calcd for $C_9H_{17}N_2O_2$, 185.1290; found, 185.1289.

***tert*-Butyl tetrahydropyrimidine-1(2*H*)-carboxylate (10)** [19]

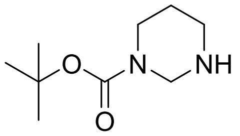

To a solution of *tert*-Butyl 5,6-dihydropyrimidine-1(4*H*)-carboxylate (**9**) (100 mg, 0.54 mmol) in MeOH (3.0 mL) was added potassium borohydride (41 mg, 1.08 mmol) at 0 °C. The reaction mixture was then allowed to warm up and was stirred at room temperature for 2 h. Ice (3.00 g) was then added and the quenched reaction mixture was stirred for 10 minutes. The solvent was evaporated *in vacuo* and the residual aqueous was extracted with dichloromethane (2 x 15 mL). The organic layer was separated and dried by passing through a hydrophobic frit. The solvent was removed *in vacuo* and the product was purified by automated flash chromatography (0–10% MeOH in DCM) to give *tert*-butyl tetrahydropyrimidine-1(2*H*)-carboxylate (**10**) as colourless oil (65 mg, 65%). TLC (MeOH/

DCM, 1: 9, v/ v), R_f = 0.18; ^{1}H NMR (400 MHz, Chloroform-*d*) δ 4.38 (s, 2H), 3.61 – 3.51 (m, 2H), 3.04 – 2.94 (m, 2H), 2.68 (s, 1H), 1.64 – 1.53 (m, 2H), 1.48 (s, 9H); IR, ν_{max} (ATR): 3312 (br), 2974, 2936, 2859, 1684 cm^{-1}; HRMS (*m/z*): [M + H]$^{+}$ calcd for $C_9H_{19}N_2O_2$, 187.1447; found, 187.1443.

tert-butyl 3-(3-cyclopentylpropanoyl)tetrahydropyrimidine-1(2*H*)-carboxylate (11)

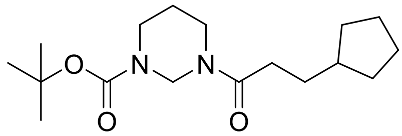

Prepared according to **general method A** (above) using *tert*-butyl tetrahydropyrimidine-1(2*H*)-carboxylate (**10**) (62.3 mg, 0.34 mmol), 3-cyclopentylpropionic acid (47.8 µl, 0.34 mmol), diisopropylethylamine (291 µL, 1.7 mmol), COMU (158 mg, 0.37 mmol) and DCM (2.5 mL). Purification by automated flash chromatography (20 – 100% EtOAc in pet. ether 40 – 60) afforded *amide* **11** as pale yellow oil (101 mg, 97%); ^{1}H NMR (400 MHz, $CDCl_3$) δ 5.05 (s, 0.4H), 4.95 (s, 1.6H), 3.69 (t, *J* = 5.6 Hz, 2H), 3.56 (t, *J* = 5.6 Hz, 2H), 2.49 (s, 1.6H), 2.33 (br s, 0.4H), 1.85 – 1.72 (m, 3H), 1.71 – 1.55 (m, 7H), 1.48 (s, 10H), 1.22 – 1.03 (m, 2H); ^{13}C NMR (125 MHz, $CDCl_3$) δ 172.4, 171.7, 153.9, 153.1, 80.6, 58.9, 58.1, 53.4, 44.3, 43.2, 41.6, 39.8, 32.5, 32.3, 31.6, 28.3, 25.1; HRMS (*m/z*): [M + H]$^{+}$ calcd for $C_{17}H_{31}N_2O_3$, 311.2335; found, 311.2341; IR, ν_{max} (ATR): 2944, 2865, 1696, 1651 cm^{-1}.

3-Cyclopentyl-1-(tetrahydropyrimidin-1(2*H*)-yl)propan-1-one (12)

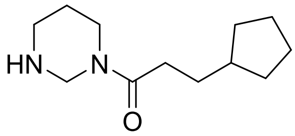

Prepared according to **general method B** (above) using compound **11** (100 mg, 0.32 mmol), DCM (3.3 mL) and TFA (1.6 mL). The solvent was evaporated *in vacuo* and the residual oil was dissolved in saturated aqueous $NaHCO_3$ (5 mL). The pH of the solution was adjusted to pH 10 using 10% aqueous NaOH and the aqueous was extracted with EtOAc (2 x 20 mL). The combined organic layers were dried by passing through a hydrophobic frit and the solvent was evaporated *in vacuo*. *Amine* **12** was isolated as a colourless gum (70 mg, 100%); ^{1}H NMR (400 MHz, $CDCl_3$) δ 4.53 (s, 1H), 4.41 (s, 1H), 3.77 – 3.67 (m, 1H), 3.67 – 3.56 (m, 1H), 3.09 – 2.97 (m, 2H), 2.70 (s, 1H), 2.40 – 2.27 (m, 2H), 1.84 – 1.71 (m, 3H), 1.71 – 1.46 (m, 8H), 1.19 – 1.02 (m, 2H); ^{13}C NMR (126 MHz, $CDCl_3$) δ 171.6, 171.0, 61.8, 57.7, 45.7,

45.5, 45.1, 41.5, 39.9, 32.6, 32.5, 31.78, 31.5, 28.3, 27.4, 25.1; IR, ν_{max} (ATR): 3300 (br), 2946, 2865, 1683, 1630 cm^{-1}; HRMS (*m/z*): [M + H]$^+$ calcd for $C_{12}H_{23}N_2O$, 211.1810; found, 211.1814.

tert-Butyl (4-(3-(3-cyclopentylpropanoyl)tetrahydropyrimidin-1(2*H*)-yl)benzyl)(methyl)carbamate (13)

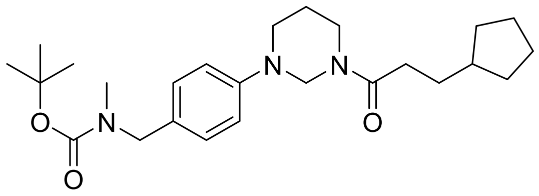

Compound **12** (37.5 mg, 0.18 mmol), *tert*-butyl-(4-bromobenzyl)(methyl)carbamate (42.9 mg, 0.8 eq), 2-(di-*tert*-butylphosphino)biphenyl (10.6 mg, 20 mol%), potassium *tert*-butoxide (28.0 mg, 1.4 eq) and palladium (II) acetate (4.0 mg, 10 mol%) were suspended in anhydrous toluene (1.5 mL) in a 2 mL microwave vial and nitrogen was bubbled through the reaction mixture for 10 min. The vial was sealed and the reaction mixture was stirred and heated to 100 °C in a microwave for 4 h. The reaction mixture was filtered through Celite™ and the Celite pad was washed with EtOAc (20 mL). The filtrate was collected and the solvent was removed *in vacuo*. The crude oil was purified by automated flash chromatography (0 – 100% EtOAc in pet. ether 40 – 60) to afford *amide* **13** as colourless gum (12.9 mg, 21%); ^{1}H NMR (400 MHz, $CDCl_3$) δ 7.25 – 7.08 (m, 2H), 7.08 – 6.87 (m, 2H), 5.00 (s, 1.3H), 4.78 (s, 0.6H), 4.42 – 4.26 (m, 2H), 3.80 – 3.69 (m, 0.7H), 3.70 – 3.59 (m, 1.3H), 3.58 – 3.48 (m, 1.3H), 3.47 – 3.36 (m, 0.7H), 2.80 (s, 3H), 2.50 – 2.39 (m, 0.7H), 2.39 – 2.29 (m, 1.3H), 1.91 – 1.70 (m, 5H), 1.71 – 1.55 (m, 3H), 1.56 – 1.44 (m, 12H), 1.18 – 1.03 (m, 2H); ^{13}C NMR (100 MHz, $CDCl_3$) δ 171.9, 156.1, 155.7, 147.0, 129.7, 128.8, 128.7, 117.6, 116.9, 79.6, 65.7, 59.8, 52.0, 51.2, 49.6, 45.4, 41.5, 39.8, 33.7, 33.7, 32.5, 32.5, 31.4, 31.3, 28.5, 25.1, 23.9, 23.8; IR, ν_{max} (ATR): 3317 (br, w), 2946, 2866, 1690, 1646, 1613 cm^{-1}; HRMS (*m/z*): [M + H]$^+$ calcd for $C_{25}H_{39}N_3O_3$, 430.3070; found, 430.3087.

***tert*-butyl methyl(4-(4-(3-oxo-3-(pyrrolidin-1-yl)propyl)piperidin-1-yl)benzyl)carbamate (14)**

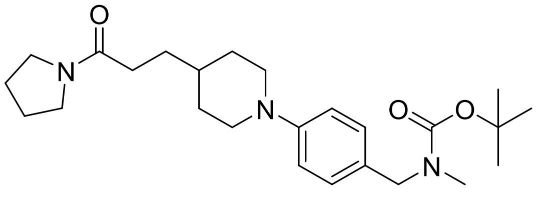

3-(Piperidin-4-yl)-1-(pyrrolidin-1-yl)propan-1-one **34** (71 mg, 0.34 mmol), *tert*-butyl (4-bromobenzyl)(methyl)carbamate (81 mg, 0.8 eq), 2-(di-*tert*-butylphosphino)biphenyl (20.1 mg, 20 mol%), potassium *tert*-butoxide (53.0 mg, 1.4 eq) and palladium (II) acetate (7.6 mg, 10 mol%) were suspended in anhydrous toluene (3.0 mL) in a 5 mL microwave vial and nitrogen was bubbled through the reaction mixture for 10 min. The vial was sealed and the reaction mixture was stirred and heated to 100 °C in a microwave for 4 h. The reaction mixture was filtered through celite and the celite pad was washed with EtOAc (20 mL). The filtrate was collected and the solvent was removed *in vacuo*. The crude oil was purified by automated flash chromatography (0 – 100% EtOAc in pet. ether 40 – 60) to afford *amide* **14** as pale yellow gum (31 mg, 21%). TLC (EtOAc, 100%), R_f = 0.14; ^{1}H NMR (400 MHz, $CDCl_3$) δ 7.15 (s, 2H), 6.90 (s, 2H), 4.35 (s, 2H), 3.68 -3.65 (m, 2H), 3.53 – 3.38 (m, 4H), 2.83 – 2.74 (m, 6H), 2.38 – 2.27 (m, 2H), 2.07 – 1.61 (m, 9H), 1.49 (s, 10H); ^{13}C NMR (125 MHz, $CDCl_3$) δ 171.4, 164.7, 156.0, 155.6, 128.8, 128.4, 116.5, 79.6, 66.7, 52.1, 51.3, 47.3, 46.6, 45.7, 38.4, 33.7, 32.4, 32.0, 31.7, 31.0, 28.5, 26.2, 24.4; IR, ν_{max} (ATR): 2925, 2858, 1689, 1635 cm^{-1}; HRMS (*m/z*): [M + H]$^+$ calcd for $C_{25}H_{39}N_3O_3$, 430.3070; found, 430.3049.

4-(4-(3-Oxo-3-(pyrrolidin-1-yl)propyl)piperidin-1-yl)benzonitrile (15)

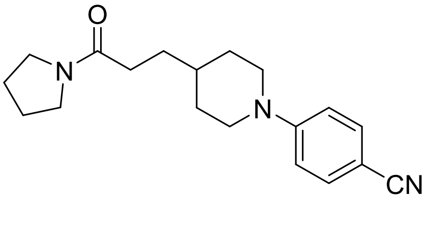

Compound **34** (20 mg, 95 μmol), 4-fluorobenzonitrile (10.4 mg, 86 μmol) and potassium carbonate (17.8 mg, 129 μmol) in DMSO (0.1 mL) were heated to 90 °C for 16 h. The crude reaction mixture was purified by automated flash chromatography (0 - 100% gradient of EtOAc in petroleum ether 40 - 60) to afford *nitrile* **15** as a colourless gum (12.1 mg, 41%). TLC (100% EtOAc), R_f = 0.17; ^{1}H NMR (400 MHz, $CDCl_3$) δ 7.50 – 7.45 (m, 2H), 6.89 –

6.82 (m, 2H), 3.91 – 3.80 (m, 2H), 3.51 – 3.46 (m, 2H), 3.46 – 3.40 (m, 2H), 2.92 – 2.81 (m, 2H), 2.37 – 2.27 (m, 2H), 2.04 – 1.93 (m, 2H), 1.93 – 1.78 (m, 4H), 1.72 – 1.51 (m, 3H), 1.39 – 1.23 (m, 2H); ^{13}C NMR (125 MHz, $CDCl_3$) δ 171.3, 153.4, 133.5, 120.3, 114.2, 99.2, 47.7, 46.6, 45.7, 35.4, 31.7, 31.5, 31.1, 26.1, 24.4; IR, ν_{max} (ATR): 2937, 2870, 2214 (CN), 1638, 1602 cm^{-1}; HRMS (*m/z*): [M + H]$^+$ calcd for $C_{19}H_{26}N_3O$, 312.2076; found, 312.2076; LCMS (ESI) [M + H]$^+$ *m/z*: 312.3, retention time = 2.11 min.

Ethyl 4-(4-(3-oxo-3-(pyrrolidin-1-yl)propyl)piperidin-1-yl)benzoate (16)

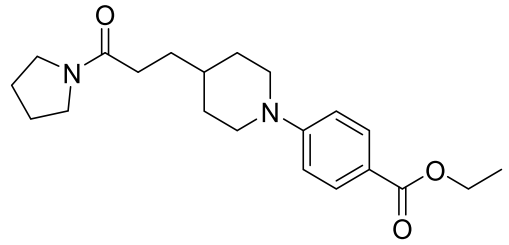

Compound **34** (26.5 mg, 126 μmol), ethyl 4-fluorobenzoate (16.6 μL, 113 μmol) and potassium carbonate (26.1 mg, 189 mmol) in anhydrous DMSO (0.1 mL) were heated to 100 °C for 3 h. After cooling to room temperature, the crude reaction mixture was purified by flash column chromatography (0 - 100% gradient of EtOAc in petroleum ether 40 - 60) to afford *ester* **16** as white amorphous solid (5.2 mg, 12%). TLC (100% EtOAc), R_f = 0.14; ^{1}H NMR (400 MHz, $CDCl_3$) δ 7.97 – 7.89 (m, 2H), 6.90 (br s, 2H), 4.35 (q, *J* = 7.1 Hz, 2H), 3.87 (d, *J* = 12.4 Hz, 2H), 3.55 – 3.38 (m, 4H), 2.86 (dd, *J* = 12.4 Hz, 2H), 2.38 – 2.28 (m, 2H), 2.03 – 1.94 (m, 2H), 1.93 – 1.79 (m, 6H), 1.73 – 1.52 (m, 3H), 1.39 (t, *J* = 7.1 Hz, 3H); ^{13}C NMR (125 MHz, $CDCl_3$) δ 171.4, 166.7, 154.2, 131.2, 119.3, 113.7, 68.0, 60.3, 48.2, 46.6, 45.7, 35.4, 31.8, 31.5, 31.2, 26.1, 25.6, 24.4, 14.5; IR, ν_{max} (ATR): 2933, 2871, 1697, 1635, 1609 cm^{-1}; HRMS (*m/z*): [M + H]$^+$ calcd for $C_{21}H_{31}N_2O_3$, 359.2335; found, 359.2328; LCMS (ESI) [M + H]$^+$ *m/z*: 359.3, retention time = 2.34 min.

***N*-((1-(4-Cyanophenyl)piperidin-4-yl)methyl)pyrrolidine-1-carboxamide (17)**

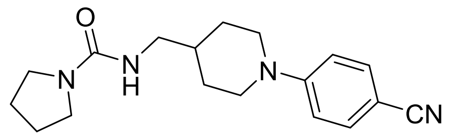

Compound **40** (20 mg, 93 μmol) and compound **36** (28.5 mg, 93 μmol) were dissolved in DCM (0.75 mL) and triethylamine (12.9 μL, 93 μmol) was added. The reaction mixture was

stirred at room temperature for 18 h, after which it was concentrated *in vacuo*. Purification by flash column chromatography (0 – 10 % MeOH in DCM) afforded the nitrile **17** as colourless oil (22.9 mg, 79 %). TLC (DCM/ MeOH, 19: 1, v/v): $R_f = 0.29$; ^{1}H NMR (400 MHz, $CDCl_3$) δ 7.48 (dd, *J* = 8.8, 2.4 Hz, 2H), 6.86 (dd, *J* = 8.8, 2.4 Hz, 2H), 4.43 – 4.28 (m, 1H), 3.96 – 3.77 (m, 2H), 3.45 – 3.27 (m, 4H), 3.25 – 3.11 (m, 2H), 2.96 – 2.75 (m, 2H), 2.07 – 1.63 (m, 7H), 1.42 – 1.19 (m, 2H); ^{13}C NMR (100 MHz, $CDCl_3$) δ 156.8, 153.3, 133.5, 120.3, 114.2, 99.2, 47.5, 45.9, 45.6, 36.7, 29.3, 25.6; IR, ν_{max} (ATR): 3386 (br, w), 2924, 2869, 2212, 1710, 1601 cm^{-1}; HRMS (*m/z*): $[M + H]^+$ calcd for $C_{18}H_{25}ON_4$, 313.2028; found, 313.2028; LCMS (ESI) $[M + H]^+$ *m/z*: 313.3, retention time = 1.87 min.

Ethyl 4-(4-((pyrrolidine-1-carboxamido)methyl)piperidin-1-yl)benzoate (18)

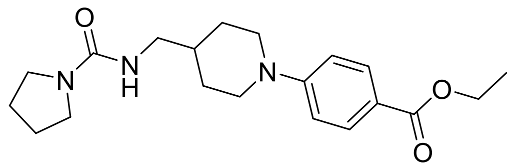

Compound **39** (24.4 mg, 93 μmol) and compound **36** (28.5 mg, 93 μmol) were dissolved in DCM (0.75 mL) and triethylamine (12.9 μL, 93 μmol) was added. The reaction mixture was stirred at room temperature for 18 h, after which it was concentrated *in vacuo*. The crude residue was purified by flash column chromatography (0 – 10 % MeOH in DCM) to afford the ester **18** as white amorphous solid (25 mg, 75 %). TLC (DCM/ MeOH, 19: 1, v/v): R_f = 0.28; ^{1}H NMR (400 MHz, $CDCl_3$) δ 7.92 (d, *J* = 8.7 Hz, 2H), 6.87 (d, *J* = 8.7 Hz, 2H), 4.43 – 4.26 (m, 3H), 3.96 – 3.78 (m, 2H), 3.43 – 3.26 (m, 4H), 3.26 – 3.11 (m, 2H), 2.92 – 2.75 (m, 2H), 1.96 – 1.88 (m, 4H), 1.88 – 1.70 (m, 3H), 1.42 – 1.26 (m, 5H); ^{13}C NMR (100 MHz, $CDCl_3$) δ 166.7, 156.9, 154.1, 131.2, 119.4, 113.8, 60.3, 48.0, 46.0, 45.5, 36.7, 29.4, 25.6, 14.4; IR, ν_{max} (ATR): 3310, 2923, 2870, 1699, 1629, 1604 cm^{-1}; HRMS (*m/z*): $[M + H]^+$ calcd for $C_{20}H_{30}O_3N_3$, 360.2287; found, 360.2285; LCMS (ESI) $[M + H]^+$ *m/z*: 360.3, retention time = 2.00 min.

***N*-((1-(4-Cyanophenyl)piperidin-4-yl)methyl)cyclopentanecarboxamide (19)**

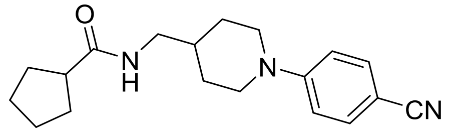

Prepared according to **general method A** using compound **40** (20 mg, 93 µmol), cyclopentaneacetic acid (15.1 µL, 139 µmol), DCM (1.0 mL), DIPEA (81 µL, 465 µmol) and COMU (43.8 mg, 102 µmol). Purification by flash column chromatography (0 – 100 % EtOAc in pet. ether 40/ 60) afforded the *nitrile* **19** as pale yellow gum (24.4 mg, 84 %). TLC (EtOAc), R_f = 0.39; ^{1}H NMR (400 MHz, $CDCl_3$) δ 7.58 – 7.45 (m, 2H), 6.95 – 6.78 (m, 2H), 5.66 – 5.52 (m, 1H), 3.96 – 3.80 (m, 2H), 3.30 – 3.14 (m, 2H), 2.94 – 2.78 (m, 2H), 2.64 – 2.44 (m, 1H), 1.97 – 1.68 (m, 8H), 1.68 – 1.53 (m, 3H), 1.44 – 1.22 (m, 2H); ^{13}C NMR (125 MHz, $CDCl_3$) δ 176.4, 153.3, 133.5, 120.2, 114.3, 99.4, 47.4, 45.9, 44.6, 36.3, 30.5, 29.2, 25.9; IR, ν_{max} (ATR): 3427, 3362 (w), 2943, 2869, 2215, 1652, 1602 cm^{-1}; HRMS (*m/z*): [M + H]$^+$ calcd for $C_{19}H_{26}N_3O$, 312.2076; found, 312.2084; LCMS (ESI) [M + H]$^+$ *m/z*: 312.3, retention time = 1.99 min.

Ethyl 4-(4-(cyclopentanecarboxamidomethyl)piperidin-1-yl)benzoate (20)

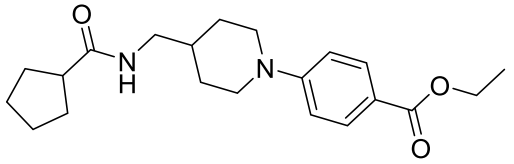

Prepared according to **general method A** (above) using compound **39** (20 mg, 76 µmol), cyclopentaneacetic acid (12.4 µL, 114 µmol), DCM (1.0 mL), DIPEA (66.4 µL, 381 µmol) and COMU (35.9 mg, 84 µmol). Saturated brine (10 mL) was used instead of distilled water during the aqueous work-up in order to achieve separation of the ethyl acetate and aqueous layers. Purification by flash column chromatography (0 – 100% EtOAc in pet. ether 40/ 60) afforded the ester **20** as white crystalline solid (18.8 mg, 69%). TLC (EtOAc/ pet. ether 40/ 60, 1: 1, v/ v), R_f = 0.48; ^{1}H NMR (400 MHz, $CDCl_3$) δ 7.92 (d, *J* = 8.8 Hz, 2H), 6.87 (d, *J* = 8.8 Hz, 2H), 5.70 – 5.56 (m, 1H), 4.41 – 4.26 (m, 2H), 3.94 – 3.83 (m, 2H), 3.26 – 3.14 (m, 2H), 2.93 – 2.76 (m, 2H), 2.60 – 2.45 (m, 1H), 1.94 – 1.70 (m, 9H), 1.65 – 1.55 (m, 2H), 1.43 – 1.29 (m, 5H); ^{13}C NMR (125 MHz, $CDCl_3$) δ 176.6, 166.7, 154.1, 131.2, 119.5, 113.8, 60.3, 47.9, 46.0, 44.8, 36.3, 30.5, 29.3, 25.9, 14.4; IR, ν_{max} (ATR): 3262, 3083 (w), 2929,

2870, 1699, 1637, 1609 cm^{-1}; HRMS (*m/z*): $[M + H]^+$ calcd for $C_{21}H_{31}N_2O_3$, 359.2329; found, 359.2332; LCMS (ESI) $[M + H]^+$ *m/z*: 359.3, retention time = 2.18 min.

***N*-((1-(Pyridin-2-yl)piperidin-4-yl)methyl)pyrrolidine-1-carboxamide (21)**

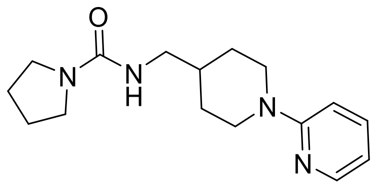

Compound **36** (41.9 mg, 0.14 mmol) was dissolved in DCM (1.0 mL) and a solution of (1-(Pyridin-2-yl)piperidin-4-yl)methanamine (**42**) (26.1 mg, 0.14 mmol) in anhydrous DCM (0.5 mL) was added followed by triethylamine (19 µL, 0.14 mmol). The reaction mixture was stirred at room temperature for 3 h. The solvent was removed *in vacuo* and the crude residue was purified by automated flash chromatography (0 – 10 % MeOH in EtOAc) to afford *urea* **21** as an off-white solid (18.8 mg, 48%). ^{1}H NMR (400 MHz, $CDCl_3$) δ 8.19 (ddd, *J* = 5.0, 2.0, 0.9 Hz, 1H), 7.46 (ddd, *J* = 8.9, 7.1, 2.0 Hz, 1H), 6.67 (dd, *J* = 8.6, 0.9 Hz, 1H), 6.58 (ddd, *J* = 7.1, 5.0, 0.9 Hz, 1H), 4.39 – 4.26 (m, 3H), 3.42 – 3.29 (m, 4H), 3.24 – 3.13 (m, 2H), 2.90 – 2.74 (m, 2H), 1.99 – 1.88 (m, 4H), 1.89 – 1.71 (m, 3H), 1.38 – 1.17 (m, 2H); ^{13}C NMR (100 MHz, $CDCl_3$) δ 159.5, 156.9, 147.9, 137.4, 112.7, 107.3, 46.1, 45.5, 45.4, 37.1, 29.6, 25.6; IR, ν_{max} (ATR): 3300 (NH), 2915, 2872, 1630, 1597 cm^{-1}; HRMS (*m/z*): $[M + H]^+$ calcd for $C_{16}H_{25}N_4O$, 289.2028; found, 289.2026.

***N*-((1-(Pyrimidin-2-yl)piperidin-4-yl)methyl)pyrrolidine-1-carboxamide (22)**

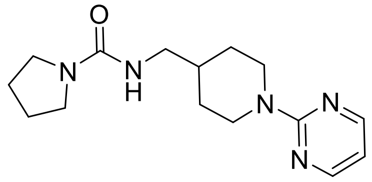

Compound **36** (0.08 g, 0.26 mmol) was dissolved in anhydrous DCM (1.5 mL) and (1-(Pyrimidin-2-yl)piperidin-4-yl)methanamine (**41**) (0.05 g, 0.26 mmol) was added followed by triethylamine (36.2 µL, 0.26 mmol). The reaction mixture was stirred at room temperature for 3 h, after which the solvent was removed *in vacuo*. The crude residue was purified by automated flash chromatography (0 – 10% MeOH in DCM) to afford compound **22** as colourless oil (31.2 mg, 41%). TLC (DCM/ MeOH, 19 : 1, v/ v), R_f = 0.18; ^{1}H NMR (400

MHz, $CDCl_3$) δ 8.30 (d, *J* = 4.7 Hz, 2H), 6.45 (t, *J* = 4.7 Hz, 1H), 4.83 – 4.71 (m, 2H), 4.33 (t, *J* = 6.1 Hz, 1H), 3.42 – 3.28 (m, 4H), 3.17 (t, *J* = 6.1 Hz, 2H), 2.96 – 2.77 (m, 2H), 1.97 – 1.87 (m, 4H), 1.88 – 1.73 (m, 3H), 1.29 – 1.12 (m, 2H); ^{13}C NMR (100 MHz, $CDCl_3$) δ 161.7, 157.7, 156.9, 109.3, 46.2, 45.5, 43.8, 37.2, 29.8, 25.6; IR, ν_{max} (ATR): 3294, 2923, 2872, 1626, 1582 cm^{-1}; HRMS (*m/z*): $[M + H]^+$ calcd for $C_{15}H_{24}N_5O$, 290.1975; found, 290.1967; LCMS (ESI) $[M + H]^+$ *m/z*: 290.3, retention time = 1.50 min.

3-(1-(4-Cyanophenyl)piperidin-4-yl)-N-(3-(piperidin-1-yl)phenyl)propanamide (23)

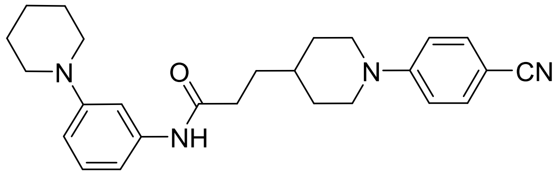

Compound **27** (20 mg, 63 μmol), 4-fluorobenzonitrile (6.6 mg, 54 μmol) and potassium carbonate (12.6 mg, 91 μmol) in anhydrous DMSO (0.1 mL) were heated to 100 °C for 3 h. The reaction mixture was cooled down to room temperature and purified by flash column chromatography to afford nitrile **23** as white solid (16.6 mg, 63 %). ^1H NMR (400 MHz, $CDCl_3$) δ 7.52 – 7.45 (m, 2H), 7.37 – 7.32 (m, 1H), 7.18 (dd, *J* = 8.3, 8.1 Hz, 1H), 7.13 (s, 1H), 6.86 (dd, *J* = 8.1, 2.4 Hz, 2H), 6.83 – 6.79 (m, 1H), 6.70 (dd, *J* = 8.3, 2.4 Hz, 1H), 3.93 – 3.80 (m, 2H), 3.24 – 3.13 (m, 4H), 2.93 – 2.81 (m, 2H), 2.46 – 2.36 (m, 2H), 1.91 – 1.79 (m, 2H), 1.80 – 1.66 (m, 5H), 1.66 – 1.53 (m, 4H), 1.42 – 1.26 (m, 2H); ^{13}C NMR (125 MHz, $CDCl_3$) δ 170.8, 153.3, 152.9, 138.7, 133.5, 129.4, 120.3, 114.2, 112.3, 110.0, 107.7, 99.2, 50.4, 47.7, 35.2, 34.7, 31.7, 31.4, 25.7, 24.3; IR, ν_{max} (ATR): 3332, 2937, 2849, 2209, 1684, 1601 cm^{-1}; HRMS (*m/z*): $[M + H]^+$ calcd for $C_{26}H_{33}N_4O$, 417.2654; found, 417.2665; LCMS (ESI) $[M + H]^+$ *m/z*: 417.3, retention time = 2.12 min.

3-(Piperidin-1-yl)aniline (24) [20]

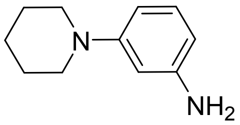

1-(3-nitrophenyl)piperidine **30** (1.0 g, 4.9 mmol) was dissolved in a mixture of methanol (6.7 ml) and EtOAc (13.3 mL) and 10% wt Pd/ C (50 mg, 5% wt) was added. The reaction

mixture was hydrogenated under H_2 (3.5 bar) at room temperature for 16 h. The catalyst was removed by filtration through Celite™ and the filtrate was concentrated *in vacuo* to afford 3-(piperidin-1-yl)aniline **24** as a colourless liquid (0.83 g, 97 %). ^{1}H NMR (400 MHz, $CDCl_3$) δ 7.05 (dd, *J* = 8.2, 7.8 Hz, 1H), 6.40 (ddd, *J* = 8.2, 2.3, 0.9 Hz, 1H), 6.30 (dd, *J* = 2.3, 2.1 Hz, 1H), 6.21 (ddd, *J* = 7.8, 2.1, 0.9 Hz, 1H), 3.59 (s, 2H), 3.18 – 3.11 (m, 4H), 1.76 – 1.66 (m, 4H), 1.63 – 1.53 (m, 2H); ^{13}C NMR (125 MHz, $CDCl_3$) δ 153.4, 147.2, 129.8, 107.4, 106.5, 103.4, 50.6, 25.8, 24.4; HRMS (*m/z*): $[M + H]^+$ calcd for $C_{11}H_{17}N_2$, 177.1392; found, 177.1389.

3-(1-(*tert*-Butoxycarbonyl)piperidin-4-yl)propanoic acid (25) [21]

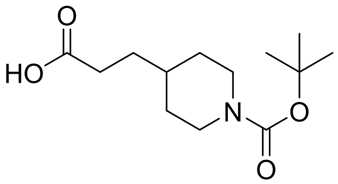

A solution of 3-(Piperidin-4-yl)propanoic acid (**32**) (100 mg, 0.64 mmol) and potassium carbonate (176 mg, 1.27 mmol) in distilled water (1.25 mL) was cooled to 0 °C and a solution of di-*tert*-butyl-dicarbonate (139 mg, 0.64 mmol) in THF (1.25 mL) was added to the reaction. The reaction mixture was stirred at room temperature for 4 h, after which the THF was removed *in vacuo* and the resulting aqueous solution was washed with diethyl ether (1 x 5 mL). The aqueous layer was acidified with 1 M HCl to pH ~ 2 and extracted with ethyl acetate (3 x 10 mL). The combined organic layers were dried using a hydrophobic frit and the solvent was evaporated *in vacuo* to afford the title compound as a colourless oil (120 mg, 73%). ^{1}H NMR (400 MHz, $CDCl_3$) δ 4.17 – 4.04 (m, 2H), 2.76 – 2.63 (m, 2H), 2.48 – 2.35 (m, 2H), 1.71 – 1.66 (m, 2H), 1.66 – 1.58 (m, 2H), 1.54 – 1.38 (m, 10H), 1.20 – 1.05 (m, 2H); ^{13}C NMR (125 MHz, $CDCl_3$) δ 179.2, 154.9, 79.4, 43.7 (br), 35.4, 31.7, 31.2, 31.1, 28.4; IR, ν_{max} (ATR): 3183 (br), 2914, 1733, 1661 cm^{-1}; HRMS (*m/z*): $[M + H]^+$ calcd for $C_{13}H_{22}NO_4$, 256.1554; found, 256.1553.

tert-Butyl-4-(3-oxo-3-((3-(piperidin-1-yl)phenyl)amino)propyl)piperidine-1-carboxylate (26)

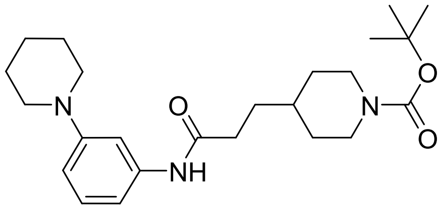

Prepared according to **general method A** using 3-(piperidin-1-yl)aniline **24** (31 mg, 0.18 mmol), 3-(1-(*tert*-Butoxycarbonyl)piperidin-4-yl)propanoic acid (**25**) (30 mg, 0.12 mmol), diisopropylethylamine (100 µL, 0.57 mmol), anhydrous DCM (2.0 mL) and COMU (55 mg, 0.13 mmol). Purification by flash column chromatography (0 – 67 % EtOAc in pet. ether 40/ 60) afforded the *amide* **26** as yellow foam (39.9 mg, 82 %). TLC (EtOAc/ pet. ether 40/ 60, 2: 1, v/ v), R_f = 0.44; ^{1}H NMR (400 MHz, Chloroform-*d*) δ 7.34 (s, 1H), 7.18 (dd, *J* = 8.4, 7.9 Hz, 1H), 7.03 (s, 1H), 6.80 (d, *J* = 7.9 Hz, 1H), 6.70 (d, *J* = 8.4 Hz, 1H), 4.11 (s, 2H), 3.26 – 3.14 (m, 4H), 2.78 – 2.62 (m, 2H), 2.46 – 2.32 (m, 2H), 1.77 – 1.66 (m, 8H), 1.65 – 1.53 (m, 3H), 1.48 (s, 9H), 1.23 – 1.06 (m, 2H); ^{13}C NMR (125 MHz, $CDCl_3$) δ 170.9, 154.8, 152.8, 138.7, 129.4, 112.4, 110.1, 107.7, 78.7, 50.5, 43.9, 43.4, 35.5, 34.8, 31.9, 28.5, 25.7, 24.3; IR, ν_{max} (ATR): 3310 (br), 2929, 2852, 1690, 1659 cm^{-1}; HRMS (*m/z*): [M + H]$^+$ calcd for $C_{24}H_{38}N_3O_3$, 416.2913; found, 416.2922; LCMS (ESI) [M + H]$^+$ *m/z*: 416.4, retention time = 1.93 min.

***N*-(3-(Piperidin-1-yl)phenyl)-3-(piperidin-4-yl)propanamide (27)**

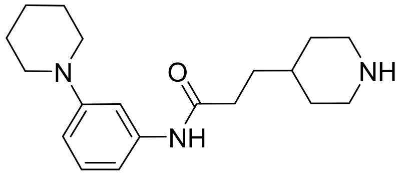

Prepared according to **general method B** using compound **26** (38 mg, 0.09 mmol), DCM (3.3 mL) and TFA (1.7 mL). *Piperidine* **27** was isolated as pale yellow oil (25.4 mg, 88 %). ^{1}H NMR (400 MHz, $CDCl_3$) δ 7.33 (dd, *J* = 2.3 Hz, 1H), 7.16 (dd, *J* = 8.1 Hz, 1H), 6.81 (dd, 1H), 6.68 (dd, *J* = 8.3, 2.3 Hz, 1H), 3.21 – 3.13 (m, 4H), 3.12 – 3.02 (m, 2H), 2.65 – 2.52 (m, 2H), 2.42 – 2.31 (m, 2H), 1.81 – 1.63 (m, 10H), 1.63 – 1.53 (m, 2H), 1.50 – 1.38 (m, 1H), 1.22 – 1.06 (m, 2H); ^{13}C NMR (125 MHz, $CDCl_3$) δ 171.3, 152.8, 138.8, 129.3, 112.2, 110.2,

107.8, 50.4, 46.7, 35.8, 34.8, 33.4, 32.6, 25.7, 24.3; IR, ν_{max} (ATR): 3245 (br), 2930, 2852, 1660, 1607 cm^{-1}; HRMS (*m/z*): $[M + H]^+$ calcd for $C_{19}H_{30}N_3O$, 316.2389; found, 316.2389.

3-(1-(4-Cyanophenyl)piperidin-4-yl)-N-(4-(piperidin-1-yl)benzyl)propanamide (28)

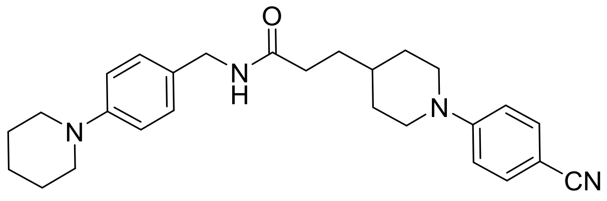

Compound **46** (27.7 mg, 84 μmol), 4-fluorobenzonitrile (9.2 mg, 76 μmol) and potassium carbonate (17.4 mg, 126 μmol) in anhydrous DMSO (0.1 mL) were heated up to 100 °C for 3 h. The reaction mixture was then cooled down to room temperature and purified by flash column chromatography to afford *nitrile* **29** as white solid (21.8 mg, 60 %). TLC (EtOAc), R_f = 0.28; ^1H NMR (400 MHz, $CDCl_3$) δ 7.51 – 7.45 (m, 2H), 7.21 – 7.14 (m, 2H), 6.91 (d, *J* = 8.2 Hz, 2H), 6.88 – 6.82 (m, 2H), 5.61 (s, 1H), 4.36 (d, *J* = 5.4 Hz, 2H), 3.90 – 3.78 (m, 2H), 3.21 – 3.10 (m, 4H), 2.91 – 2.78 (m, 2H), 2.29 – 2.21 (m, 2H), 1.88 – 1.45 (m, 11H), 1.37 – 1.22 (m, 2H); ^{13}C NMR (125 MHz, $CDCl_3$) δ 172.3, 153.3, 151.7, 133.5, 128.9, 128.4, 120.3, 116.5, 114.2, 99.3, 50.6, 47.7, 43.3, 35.3, 33.8, 32.0, 31.4, 25.7, 24.2; HRMS (*m/z*): $[M + H]^+$ calcd for $C_{27}H_{35}N_4O$, 431.2811; found, 431.2830; LCMS (ESI) $[M + H]^+$ *m/z*: 431.4, retention time = 1.64 min.

3-(1-(4-Cyanophenyl)piperidin-4-yl)-N-(3-(piperidin-1-yl)benzyl)propanamide (29)

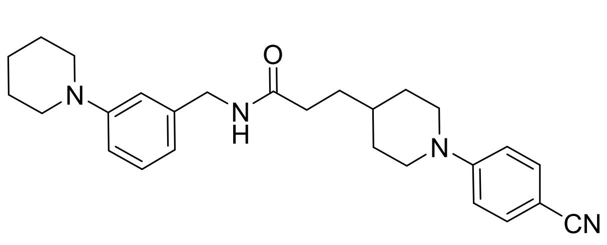

Compound **43** (20 mg, 61 μmol), 4-fluorobenzonitrile (6.6 mg, 54 μmol) and potassium carbonate (12.6 mg, 91 μmol) in anhydrous DMSO (0.1 mL) were heated up to 100 °C for 3 h. The reaction mixture was then cooled down to room temperature and purified by flash column chromatography to afford *nitrile* **28** as colourless gum (19.8 mg, 84%). ^1H NMR (400 MHz, $CDCl_3$) δ 7.51 – 7.45 (m, 2H), 7.26 – 7.19 (m, 1H), 6.90 – 6.82 (m, 4H), 6.76 – 6.71

(m, 1H), 5.67 (t, *J* = 5.7 Hz, 1H), 4.40 (d, *J* = 5.7 Hz, 2H), 3.90 – 3.79 (m, 2H), 3.22 – 3.12 (m, 4H), 2.91 – 2.77 (m, 2H), 2.31 – 2.23 (m, 2H), 1.86 – 1.76 (m, 2H), 1.76 – 1.49 (m, 9H), 1.37 – 1.24 (m, 2H); ^{13}C NMR (125 MHz, $CDCl_3$) δ 172.3, 153.3, 152.6, 139.0, 133.5, 129.4, 120.3, 118.5, 116.0, 115.5, 114.2, 99.3, 50.5, 47.7, 44.2, 35.3, 33.8, 32.0, 31.4, 25.8, 24.2; IR, ν_{max} (ATR): 3279 (br), 2925, 2852, 2214, 1638, 1603 cm^{-1}; HRMS (*m/z*): $[M + H]^+$ calcd for $C_{27}H_{35}N_4O$, 431.2811; found, 431.2795; LCMS (ESI) $[M + H]^+$ *m/z*: 431.3, retention time = 2.01 min.

1-(3-Nitrophenyl)piperidine (30) [20]

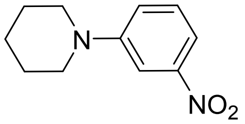

To a solution of 1-fluoro-3-nitrobenzene (0.75 µM, 7.1 mmol) in DMSO (7.0 mL) were added potassium carbonate (1.47 g, 10.6 mmol) and piperidine (1.4 mL, 14.2 mmol). The reaction mixture was stirred at 90 °C for 40 h. After cooling to room temperature, the mixture was diluted with EtOAc (60 mL) and washed with water (2 x 50 mL) and saturated brine (50 mL). The organic layer was dried over anhydrous Na_2SO_4 and concentrated *in vacuo*. Purification by flash column chromatography (0 – 10 % EtOAc in pet. ether 40 - 60) afforded 1-(3-nitrophenyl)piperidine **30** as an orange liquid (1.34 g, 92%). ^{1}H NMR (400 MHz, $CDCl_3$) δ 7.70 (dd, *J* = 2.1, 2.5 Hz, 1H), 7.59 (ddd, *J* = 8.2, 2.1, 0.9 Hz, 1H), 7.33 (dd, *J* = 8.2, 8.2 Hz, 1H), 7.18 (ddd, *J* = 8.2, 2.5, 0.9 Hz, 1H), 3.32 – 3.20 (m, 4H), 1.77 – 1.67 (m, 4H), 1.68 – 1.57 (m, 2H); ^{13}C NMR (125 MHz, $CDCl_3$) δ 152.4, 149.3, 129.5, 121.4, 112.9, 109.8, 49.7, 25.4, 24.1; HRMS (*m/z*): $[M + H]^+$ calcd for $C_{11}H_{15}N_2O_2$, 207.1134; found, 207.1135.

3-(Piperidin-4-yl)propanoic acid, (32) [22]

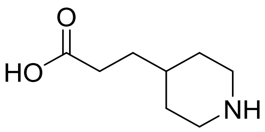

3-(4-Pyridine)acrylic acid (**31**) (1.0 g, 6.7 mmol) was suspended in distilled water (4.0 mL) and the solution was adjusted to pH ~ 6-7 using 35% wt aqueous ammonia (0.3 ml). To the

above suspension was added rhodium on activated alumina (100 mg) in distilled water (1.0 ml) and the resulting reaction mixture was hydrogenated under H_2 (8 bar) at 95 °C for 48 h. After cooling to room temperature, the catalyst was removed by filtration through Celite™ and the solvent was removed *in vacuo* using a freeze-drier to afford 3-(piperidin-4-yl)propanoic acid (**32**) as an off-white solid (1.06g, > 99%). ^{1}H NMR (400 MHz, Methanol-d_4) δ 3.40 – 3.34 (m, 2H), 3.01 – 2.89 (m, 2H), 2.26 – 2.18 (m, 2H), 2.03 – 1.92 (m, 2H), 1.67 – 1.57 (m, 3H), 1.46 – 1.30 (m, 2H); ^{13}C NMR (125 MHz, $CDCl_3$) δ 181.9, 45.3, 35.7, 34.8, 33.6, 29.9; HRMS (*m/z*): $[M + H]^+$ calcd for $C_8H_{16}NO_2$, 158.1176; found, 158.1173.

tert-Butyl 4-(3-oxo-3-(pyrrolidin-1-yl)propyl)piperidine-1-carboxylate (33)

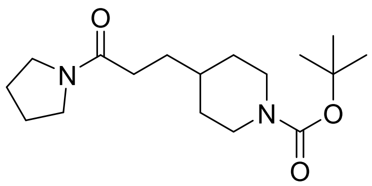

Prepared according to **general method A** (above) using 3-(1-(*tert*-Butoxycarbonyl)piperidin-4-yl)propanoic acid (**25**) (95.4 mg, 0.37 mmol), pyrrolidine (40 µl, 0.37 mmol), diisopropylethylamine (323 µl, 1.85 mmol), DCM (5.0 ml) and COMU (175 mg, 0.41 mmol). Purification by flash column chromatography (0-10% gradient of MeOH in DCM) afforded *amide* (**33**) as a colourless oil (99 mg, 86%). ^{1}H NMR (400 MHz, $CDCl_3$) δ 4.09 (br s, 2H), 3.48 (t, J = 6.8 Hz, 2H), 3.42 (t, J = 6.8 Hz, 2H), 2.79 – 2.62 (m, 2H), 2.36 – 2.25 (m, 2H), 1.98 (dd, J = 6.8 Hz, 6.8 Hz, 2H), 1.87 (dd, J = 6.8 Hz, 6.8 Hz, 2H), 1.75 – 1.57 (m, 4H), 1.54 – 1.41 (m, 10H), 1.22 – 1.05 (m, 2H); ^{13}C NMR (125 MHz, $CDCl_3$) δ 154.9, 79.2, 66.9, 47.3, 46.7, 45.7, 43.9 (br), 38.4, 35.7, 32.0, 31.3, 28.5, 26.2, 24.4; IR, ν_{max} (ATR): 2967, 2943, 2861, 1689, 1629 cm^{-1}; HRMS (*m/z*): $[M + H]^+$ calcd for $C_{17}H_{31}N_2O_3$, 311.2335; found, 311.2327.

3-(Piperidin-4-yl)-1-(pyrrolidin-1-yl)propan-1-one (34)

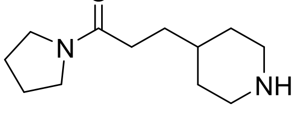

tert-Butyl-4-(3-oxo-3-(pyrrolidin-1-yl)propyl)piperidine-1-carboxylate (**33**) (392 mg, 1.26 mmol) was dissolved in DCM (6.6 mL) and TFA (3.3 mL) was added. The reaction mixture was stirred at room temperature for 2 h, after which time it was concentrated and dried *in*

vacuo. The residue was re-dissolved in DCM (20 mL) and potassium carbonate (1.00 g) was added and the resulting mixture was stirred vigorously for 60 h. The slurry was filtered through a hydrophobic frit and the filtrate was concentrated and dried *in vacuo* to afford *piperidine* **34** as a white amorphous solid (189 mg, 99%). The compound was used without further purification ^{1}H NMR (400 MHz, $CDCl_3$) δ 3.44 – 3.31 (m, 4H), 3.06 – 2.97 (m, 2H), 2.59 (s, 1H), 2.57 – 2.49 (m, 2H), 2.26 – 2.16 (m, 2H), 1.96 – 1.85 (m, 2H), 1.85 – 1.74 (m, 2H), 1.69 – 1.59 (m, 2H), 1.59 – 1.49 (m, 2H), 1.44 – 1.29 (m, 1H), 1.17 – 1.01 (m, 2H); ^{13}C NMR (125 MHz, $CDCl_3$) δ 171.5, 66.5, 47.1, 46.3, 45.4, 38.2, 35.6, 32.9, 31.7, 31.6, 25.9, 24.2; IR, ν_{max} (ATR): 3278 (br), 2931, 2869, 2776, 1626 cm^{-1}; HRMS (*m/z*): $[M + H]^+$ calcd for $C_{12}H_{23}N_2O$, 211.1810; found, 211.1820.

(1*H*-imidazol-1-yl)(pyrrolidin-1-yl)methanone (35) [23]

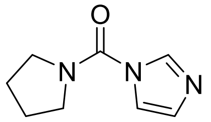

To a suspension of 1,1'-carbonyl diimidazole (1.00 g, 6.2 mmol) in anhydrous THF (10 mL was added pyrrolidine (0.46 mL, 5.5 mmol) and the reaction mixture was heated at reflux for 18 h. After cooling to room temperature, the solvent was evaporated *in vacuo* and the residue was re-dissolved in DCM (10 mL) and washed with water (2 x 10 mL). The organic phase was concentrated *in vacuo* to give (1*H*-imidazol-1-yl)(pyrrolidin-1-yl)methanone (**35**) as an off-white solid which was used without further purification (0.68 g, 67%). ^{1}H NMR (400 MHz, $CDCl_3$) δ 8.03 (dd, J = 1.1, 1.2 Hz, 1H), 7.37 (dd, J = 1.1, 1.4 Hz, 1H), 7.09 (dd, J = 1.2, 1.4 Hz, 1H), 3.70 – 3.60 (m, 4H), 2.07 – 1.95 (m, 4H); ^{13}C NMR (125 MHz, $CDCl_3$) δ 149.7, 136.7, 129.4, 117.6, 48.9 (br), 25.5 (br); HRMS (*m/z*): $[M + H]^+$ calcd for $C_8H_{12}N_3O$, 166.0980; found, 166.0983.

3-Methyl-1-(pyrrolidine-1-carbonyl)-1*H*-imidazol-3-ium iodide (36) [23]

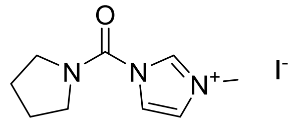

1-(Pyrrolidin-1-ylcarbonyl)-1*H*-imidazole **35** (623 mg, 3.8 mmol) was dissolved in anhydrous acetonitrile (7.5 mL) and iodomethane (1.0 mL, 16 mmol) was added. The reaction mixture was stirred at room temperature for 40 h. The solvent was then evaporated *in vacuo* to give 3-Methyl-1-(pyrrolidine-1-carbonyl)-1*H*-imidazol-3-ium iodide (**36**) as a pale yellow amorphous solid (1.16 g, 92%). ^{1}H NMR (400 MHz, $CDCl_3$) δ 10.44 (dd, *J* = 1.4, 1.4 Hz, 1H), 7.85 (dd, *J* = 1.9, 1.4 Hz, 1H), 7.58 (dd, *J* = 1.9, 1.4 Hz, 1H), 4.33 (s, 3H), 4.02 (s, 2H), 3.69 (s, 2H), 2.12 – 2.04 (m, 4H); ^{13}C NMR (125 MHz, $CDCl_3$) δ 144.7, 136.5, 123.6, 120.9, 50.8, 49.3, 38.1, 26.4, 24.1; HRMS (*m/z*): [M - I]$^+$ calcd for $C_9H_{14}N_3O$, 180.1137; found, 180.1136.

Ethyl 4-(4-(((*tert*-butoxycarbonyl)amino)methyl)piperidin-1-yl)benzoate (37)

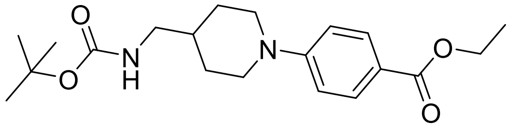

4-(*N*-Boc-aminomethyl)-piperidine (200 mg, 0.93 mmol) was dissolved in anhydrous DMSO (1.0 mL). Ethyl 4-fluorobenzoate (123 µL, 0.84 mmol) and potassium carbonate (193 mg, 1.40 mmol) were added and the reaction mixture was heated to 90 °C for 22 h. The reaction mixture was then cooled to room temperature and purified by flash column chromatography (0 – 100 % EtOAc in pet. ether 40/ 60) to afford *ester* **37** as a white solid (187 mg, 55 %). TLC (EtOAc/ pet. ether 40/ 60, 1: 1, v/ v), R_f = 0.53; ^{1}H NMR (400 MHz, $CDCl_3$) δ 7.99 – 7.86 (m, 2H), 6.93 – 6.78 (m, 2H), 4.65 (s, 1H), 4.44 – 4.27 (m, 2H), 3.97 – 3.81 (m, 2H), 3.14 – 2.99 (m, 2H), 2.93 – 2.75 (m, 2H), 1.86 – 1.77 (m, 2H), 1.71 (s, 1H), 1.47 (s, 9H), 1.43 – 1.27 (m, 5H); ^{13}C NMR (125 MHz, $CDCl_3$) δ 166.7, 156.0, 154.1, 131.2, 119.5, 113.8, 79.3, 60.3, 47.9, 46.0, 36.6, 29.3, 28.4, 14.5; IR, ν_{max} (ATR): 3333, 2980, 2921, 2865, 1699, 1674, 1605 cm^{-1}; HRMS (*m/z*): [M + H]$^+$ calcd for $C_{20}H_{31}N_2O_4$, 363.2284; found, 363.2268; LCMS (ESI) [M + H]$^+$ *m/z*: 363.3, retention time = 2.35 min.

***tert*-Butyl ((1-(4-cyanophenyl)piperidin-4-yl)methyl)carbamate (38)**

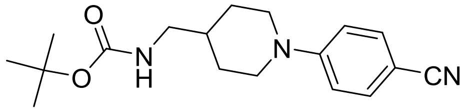

4-(*N*-Boc-aminomethyl)-piperidine (200 mg, 0.93 mmol), 4-fluorobenzonitrile (102 mg, 0.84 mmol) and potassium carbonate (193 mg, 1.40 mmol) in DMSO (1.0 mL) were heated to 90 °C for 2 h. The reaction mixture was then cooled to room temperature and purified by flash column chromatography (0 – 50 % EtOAc in pet. ether 40/ 60) to afford *nitrile* **38** as white solid (217 mg, 74 %). TLC (EtOAc/ pet. ether 40/ 60, 1: 1, v/ v), R_f = 0.45; ^{1}H NMR (400 MHz, $CDCl_3$) δ 7.52 – 7.46 (m, 2H), 6.90 – 6.83 (m, 2H), 4.64 (s, 1H), 3.95 – 3.82 (m, 2H), 3.15 – 3.02 (m, 2H), 2.97 – 2.79 (m, 2H), 1.88 – 1.78 (m, 2H), 1.73 (s, 1H), 1.47 (s, 9H), 1.41 – 1.22 (m, 2H); ^{13}C NMR (100 MHz, $CDCl_3$) δ 156.0, 153.3, 133.5, 120.2, 114.2, 99.4, 79.3, 47.4, 45.9, 36.6, 29.1, 28.4; IR, ν_{max} (ATR): 3364, 2909, 2206, 1686, 1605 cm^{-1}; HRMS (*m/z*): $[M + H]^+$ calcd for $C_{18}H_{26}N_3O_2$, 316.2020; found, 316.2017; LCMS (ESI) $[M + H]^+$ *m/z*: 316.2, retention time = 2.17 min.

Ethyl 4-(4-(aminomethyl)piperidin-1-yl)benzoate (39)

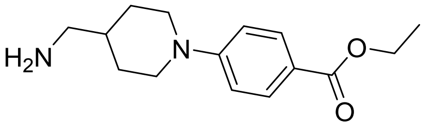

Compound **37** (175 mg, 0.48 mmol) was dissolved in DCM (6.6 mL) and TFA (2.0 mL) was added. The reaction mixture was stirred at room temperature for 2 h, after which it was concentrated *in vacuo*. Water (10 mL) was added to the residue and the pH was adjusted to pH 14 using 10% aqueous NaOH. The aqueous was extracted with EtOAc (2 x 30 mL) and the organic phase was dried by passing through a hydrophobic frit. The solvent was evaporated *in vacuo* to give *ester* **39** as white solid (126 mg, 100 %). ^{1}H NMR (400 MHz, $CDCl_3$) δ 7.99 – 7.86 (m, 2H), 6.94 – 6.81 (m, 2H), 4.42 – 4.25 (m, 2H), 3.98 – 3.83 (m, 2H), 2.95 – 2.78 (m, 2H), 2.73 – 2.57 (m, 2H), 1.93 – 1.78 (m, 2H), 1.69 (s, 2H), 1.63 – 1.51 (m, 1H), 1.42 – 1.26 (m, 5H); ^{13}C NMR (125 MHz, $CDCl_3$) δ 166.7, 154.2, 131.2, 119.4, 113.7, 60.3, 48.0, 47.8, 39.1, 29.4, 14.4; HRMS (*m/z*): $[M + H]^+$ calcd for $C_{15}H_{23}N_2O_2$, 263.1754; found, 263.1753.

4-(4-(Aminomethyl)piperidin-1-yl)benzonitrile (40)

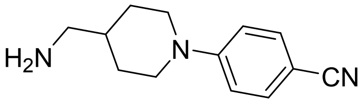

Compound **38** (192 mg, 0.61 mmol) was dissolved in DCM (6.6 mL) and TFA (3.3 mL) was added. The reaction mixture was stirred at room temperature for 45 min, after which it was concentrated *in vacuo*. Water (10 mL) was added to the residue and the pH was adjusted to pH 14 using 10% aqueous NaOH. The aqueous solution was extracted with EtOAc (2 x 30 mL) and the combined organic layers were dried by passing through a hydrophobic frit. The solvent was evaporated *in vacuo* to give *amine* **40** as white solid (120 mg, 92 %). ^{1}H NMR (400 MHz, $CDCl_3$) δ 7.55 – 7.43 (m, 2H), 6.95 – 6.81 (m, 2H), 3.95 – 3.82 (m, 2H), 3.00 – 2.78 (m, 2H), 2.70 – 2.57 (m, 2H), 1.93 – 1.80 (m, 2H), 1.63 – 1.51 (m, 1H), 1.37 – 1.23 (m, 4H); ^{13}C NMR (125 MHz, $CDCl_3$) δ 153.3, 133.5, 120.3, 114.2, 99.2, 47.8, 47.6, 39.3, 29.3; IR, ν_{max} (ATR): 3381, 3317 (w), 2917, 2821, 2209, 1688 (w), 1602 cm^{-1}; HRMS (*m/z*): [M + H]$^+$ calcd for $C_{13}H_{18}N_3$, 216.1501; found, 216.1507.

(1-(Pyrimidin-2-yl)piperidin-4-yl)methanamine (41) [24]

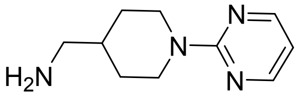

Acetophenone (1.02 mL, 8.8 mmol), *p*-toluenesulfonic acid (17 mg, 1 mol%), 4-(aminomethyl) piperidine (1.00 g, 8.8 mmol) and toluene (9 mL) were heated to reflux under Dean-Stark conditions for 5 h. The reaction mixture was then cooled to room temperature and its volume was measured to be 12.0 ml. An aliquot of the above solution (1.2 mL) was taken, 2-chloropyrimidine (100 mg, 0.87 mmol) and triethylamine (0.61 mL, 4.4 mmol) were added and the reaction mixture was heated at 95 °C for 18 h. After cooling to room temperature, the reaction mixture was extracted with 5 M HCl (2 x 9 mL). The combined aqueous layers were washed with diethyl ether (20 mL) and the basified with 10% wt aqueous NaOH to pH 12-14. The basic aqueous solution was extracted with EtOAc (2 x 30 mL). The combined organic layers were dried with anhydrous $MgSO_4$ and evaporated *in vacuo* to give a brown gum, which was purified by flash column chromatography (0 – 20% 7 M methanolic ammonia in DCM) to give amine **41** as colourless gum (80 mg, 48%). TLC (DCM/ 7M NH_3 in MeOH, 9 : 1, v/ v), R_f = 0.30; ^{1}H NMR (400 MHz, $CDCl_3$) δ 8.29 (d, *J* = 4.7 Hz, 2H), 6.44 (dd, *J* = 4.7,

4.7 Hz, 1H), 4.85 – 4.70 (m, 2H), 2.94 – 2.82 (m, 2H), 2.66 – 2.59 (m, 2H), 1.87 – 1.78 (m, 2H), 1.72 – 1.53 (m, 3H), 1.26 – 1.11 (m, 2H); ^{13}C NMR (100 MHz, $CDCl_3$) δ 161.7, 157.7, 109.3, 48.0, 43.9, 39.8, 29.7; IR, ν_{max} (ATR): 3328, 2911, 2847, 1622 (w), 1583, 1542 cm^{-1}; HRMS (*m/z*): $[M + H]^+$ calcd for $C_{10}H_{17}N_4$, 193.1453; found, 193.1452.

(1-(Pyridin-2-yl)piperidin-4-yl)methanamine (42) [24]

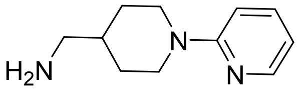

4-(Aminomethyl)piperidine (100 mg, 0.88 mmol) was dissolved in 1-pentanol (1.0 mL) and sodium carbonate followed by 2-chloropyridine (82 µL, 0.88 mmol) were added. The reaction mixture was then stirred at 95 °C for 15 h and then at 130 °C for a further 24 h. After cooling to room temperature, the reaction mixture was concentrated *in vacuo* to give a slurry (approx. 1 mL), which was purified by flash column chromatography (0 – 10% 7N methanolic ammonia in DCM) to give amine **42** as colourless oil (34.6 mg, 21%). TLC (DCM/ 7M NH_3 in MeOH, 9 : 1, v/ v), R_f = 0.15; ^{1}H NMR (400 MHz, $CDCl_3$) δ 8.19 (ddd, *J* = 5.0, 2.0, 0.9 Hz, 1H), 7.47 (ddd, *J* = 8.9, 7.1, 2.0 Hz, 1H), 6.68 (ddd, *J* = 8.9, 0.9, 0.9 Hz, 1H), 6.59 (ddd, *J* = 7.1, 5.0, 0.9 Hz, 1H), 4.40 – 4.26 (m, 2H), 2.92 – 2.78 (m, 2H), 2.69 – 2.59 (m, 2H), 1.91 – 1.79 (m, 2H), 1.67 – 1.40 (m, 3H), 1.37 – 1.17 (m, 2H); ^{13}C NMR (100 MHz, $CDCl_3$) δ 159.6, 148.0, 137.4, 112.6, 107.6, 48.1, 45.5, 49.7, 29.5; IR, ν_{max} (ATR): 3336, 3002, 2913, 2846, 1591, 1559 cm^{-1}; HRMS (*m/z*): $[M + H]^+$ calcd for $C_{11}H_{18}N_3$, 192.1501; found, 192.1501.

(4-(Piperidin-1-yl)phenyl)methanamine (43) [20]

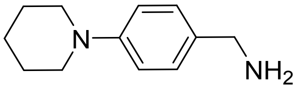

4-(piperidin-1-yl)benzonitrile **49** (0.20 g, 1.1 mmol) was dissolved in anhydrous THF (1.0 mL) and cooled in ice/ water. Red-Al (65 % in toluene, 1.0 mL, 3.2 mmol) was added dropwise. The reaction mixture was warmed up to room temperature and stirred for for 2 h. The reaction mixture was cooled in ice/ water and quenched by the slow addition of water (1.0 mL). The resulting mixture was filtered through Celite and washed with THF (3 x 10 mL). The filtrate was concentrated in vacuo to afford (4-(piperidin-1-yl)phenyl)methanamine

43 as yellow liquid (220 mg), which was used in subsequent steps without further purification. ^{1}H NMR (400 MHz, $CDCl_3$) δ 7.23 – 7.18 (m, 2H), 6.96 – 6.91 (m, 2H), 3.79 (s, 2H), 3.20 – 3.11 (m, 4H), 1.78 – 1.67 (m, 4H), 1.65 – 1.41 (m, 4H); HRMS (*m/z*): $[M + H]^+$ calcd for $C_{12}H_{19}N_2$, 191.1548; found, 191.1556.

***tert*-Butyl 4-(3-oxo-3-((4-(piperidin-1-yl)benzyl)amino)propyl)piperidine-1-carboxylate (44)**

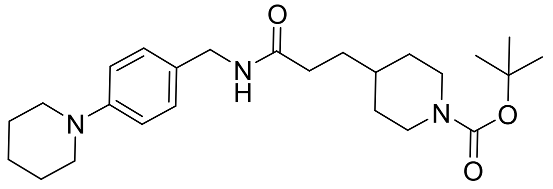

Prepared according to **general method A** using 4-(piperidin-1-yl)phenyl)methanamine **43** (36.9 mg, 0.19 mmol), 3-(1-(*tert*-Butoxycarbonyl)piperidin-4-yl)propanoic acid (**25**) (50 mg, 0.19 mmol), diisopropylethylamine (169 µL, 0.97 mmol), anhydrous DCM (2.5 mL) and COMU (92 mg, 0.21 mmol). Purification by flash column chromatography (0 – 100 % EtOAc in pet. ether 40/ 60) afforded the *amide* **44** as a colourless oil (49.7 mg, 60 %). TLC (100 % EtOAc), R_f = 0.34; ^{1}H NMR (400 MHz, $CDCl_3$) δ 7.20 – 7.14 (m, 2H), 6.94 – 6.89 (m, 2H), 5.57 (s, 1H), 4.40 – 4.31 (m, 2H), 4.11 (s, 2H), 3.22 – 3.11 (m, 4H), 2.77 – 2.58 (m, 2H), 2.29 – 2.17 (m, 2H), 1.76 – 1.59 (m, 10H), 1.47 (s, 10H), 1.19 – 1.03 (m, 2H); ^{13}C NMR (125 MHz, $CDCl_3$) δ 172.4, 154.8, 151.7, 128.9, 128.4, 116.5, 79.3, 50.5, 44.2, 43.3, 35.6, 33.8, 32.1, 28.5, 25.7, 24.2; HRMS (*m/z*): $[M + H]^+$ calcd for $C_{25}H_{40}N_3O_3$, 430.3070; found, 430.3068.

***N*-(4-(Piperidin-1-yl)benzyl)-3-(piperidin-4-yl)propanamide (45)**

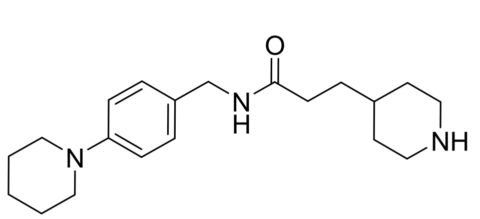

Prepared according to **general method B** using compound **44** (48 mg, 0.11 mmol), DCM (3.3 mL) and TFA (1.7 mL). *Piperidine* **45** was obtained as off-white solid (29.4 mg, 80 %). ^{1}H NMR (400 MHz, $CDCl_3$) δ 7.23 – 7.12 (m, 2H), 6.98 – 6.84 (m, 2H), 5.62 (s, 1H), 4.40 –

4.28 (m, 2H), 3.20 – 3.11 (m, 4H), 3.12 – 3.02 (m, 2H), 2.63 – 2.53 (m, 2H), 2.27 – 2.17 (m, 2H), 1.81 – 1.52 (m, 11H), 1.48 – 1.33 (m, 1H), 1.19 – 1.03 (m, 2H); ^{13}C NMR (125 MHz, $CDCl_3$) δ 172.7, 151.7, 129.1, 128.5, 116.5, 50.6, 46.6, 43.2, 35.9, 33.9, 33.3, 32.9, 25.7, 24.2; IR, ν_{max} (ATR): 3311, 2922, 2850, 2812, 1642, 1616 cm^{-1}; HRMS (*m/z*): $[M + H]^+$ calcd for $C_{20}H_{32}N_3O$, 330.2545; found, 330.2559.

(3-(Piperidin-1-yl)phenyl)methanamine (46) [20]

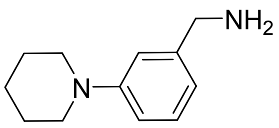

3-(piperidin-1-yl)benzonitrile **50** (0.30 g, 1.6 mmol) was dissolved in anhydrous THF (1.5 mL) and cooled in ice/ water. Red-Al (65 % in toluene, 1.5 mL, 4.9 mmol) was added dropwise. The reaction mixture was warmed up and stirred at room temperature for 2 h. The reaction mixture was cooled in ice/ water and quenched by the slow addition of water (1.0 ml). The resulting mixture was filtered through Celite and washed with THF (3 x 10 mL). The filtrate was concentrated *in vacuo* to afford crude (3-(Piperidin-1-yl)phenyl)methanamine **46** as dark yellow oil, which was used in subsequent steps without further purification; ^{1}H NMR (400 MHz, $CDCl_3$) δ 7.23 (dd, J = 7.6, 7.6 Hz, 1H), 6.92 (dd, J = 2.0, 1.3 Hz, 1H), 6.79 (dd, J = 7.6, 2.0 Hz, 1H), 6.79 (dd, J = 7.6, 1.3 Hz, 1H), 3.84 (s, 2H), 3.24 – 3.14 (m, 4H), 1.79 – 1.67 (m, 6H), 1.66 – 1.51 (m, 2H); HRMS (*m/z*): $[M + H]^+$ calcd for $C_{12}H_{19}N_2$, 191.1548; found, 191.1539.

***tert*-Butyl 4-(3-oxo-3-((3-(piperidin-1-yl)benzyl)amino)propyl)piperidine-1-carboxylate (47)**

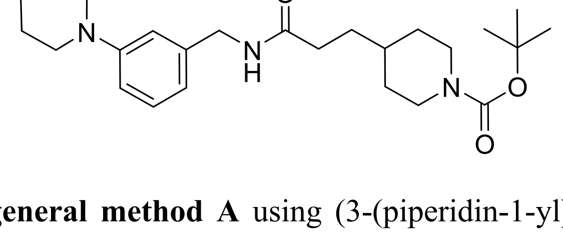

Prepared according to **general method A** using (3-(piperidin-1-yl)phenyl)methanamine **46** (33 mg, 0.17 mmol), 3-(1-(*tert*-Butoxycarbonyl)piperidin-4-yl)propanoic acid (**25**) (30 mg, 0.12 mmol), diisopropylethylamine (100 µL, 0.57 mmol), anhydrous DCM (2.0 mL) and

COMU (55 mg, 0.13 mmol). Purification by flash column chromatography (0 – 67 % EtOAc in pet. ether 40/ 60) afforded the *amide* **47** as colourless oil (46.8 mg, 94 %). TLC (EtOAc/ pet. ether 40/ 60, 2: 1, v/ v), R_f = 0.19; ^{1}H NMR (400 MHz, $CDCl_3$) δ 7.26 – 7.20 (m, 1H), 6.90 – 6.85 (m, 2H), 6.76 – 6.71 (m, 1H), 5.64 (s, 1H), 4.44 – 4.35 (m, 2H), 4.09 (s, 2H), 3.23 – 3.12 (m, 4H), 2.77 – 2.60 (m, 2H), 2.32 – 2.17 (m, 2H), 1.80 – 1.53 (m, 10H), 1.47 (s, 10H), 1.19 – 1.02 (m, 2H); ^{13}C NMR (125 MHz, $CDCl_3$) δ 172.5, 154.9, 152.5, 139.0, 129.4, 118.6, 116.0, 115.0, 79.3, 50.9, 50.5, 44.2, 35.6, 33.7, 32.3, 31.9, 28.5, 25.7, 24.2; IR, ν_{max} (ATR): 3313 (br), 2933, 2856, 1655 cm^{-1}; HRMS (*m/z*): $[M + H]^+$ calcd for $C_{25}H_{40}N_3O_3$, 430.3070; found, 430.3090.

N-(3-(Piperidin-1-yl)benzyl)-3-(piperidin-4-yl)propanamide (48)

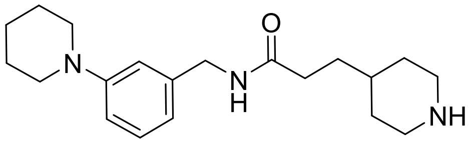

Prepared according to **general method B** using compound **47** (43 mg, 0.10 mmol), DCM (3.3 mL) and TFA (1.7 mL). *Piperidine* **48** was isolated as colourless oil (21.3 mg, 65 %). ^{1}H NMR (400 MHz, $CDCl_3$) δ 7.25 – 7.17 (m, 1H), 6.90 – 6.82 (m, 2H), 6.73 (d, *J* = 7.4 Hz, 1H), 5.75 (s, 1H), 4.39 (d, *J* = 5.5 Hz, 2H), 3.21 – 3.12 (m, 4H), 3.12 – 3.01 (m, 2H), 2.65 – 2.51 (m, 2H), 2.28 – 2.18 (m, 2H), 2.03 (s, 1H), 1.77 – 1.53 (m, 10H), 1.48 – 1.33 (m, 1H), 1.20 – 1.03 (m, 2H). ^{13}C NMR (125 MHz, $CDCl_3$) δ 172.8, 152.6,139.1, 129.4, 118.5, 116.0, 115.5, 50.5, 46.6, 44.1, 35.9, 33.8, 33.2, 32.9, 25.8, 24.3; IR, ν_{max} (ATR): 3271 (br), 2927, 2854, 1640, 1600, 1554 cm^{-1}; HRMS (*m/z*): $[M + H]^+$ calcd for $C_{20}H_{32}N_3O$, 330.2545; found, 330.2556.

4-(Piperidin-1-yl)benzonitrile (49) [20]

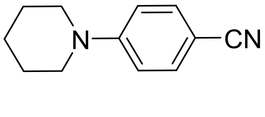

4-Fluorobenzonitrile (0.50 g, 4.1 mmol) and piperidine (0.82 mL, 8.3 mmol) were dissolved in DMSO (3.5 mL). Potassium carbonate (0.86 g, 6.2 mmol) was added and the reaction mixture was heated up to 90 °C for 8 h. After cooling to room temperature, the reaction mixture was diluted with EtOAc (30 mL) and washed with water (2 x 25 mL) and saturated

brine (25 mL). The organic phase was dried by passing through a hydrophobic frit and evaporated *in vacuo* to afford 4-(piperidin-1-yl)benzonitrile **49** as an off-white solid (0.78 g, > 99%). TLC (EtOAc/ pet. ether 40/ 60, 1: 9, v/ v), R_f = 0.29; ^{1}H NMR (400 MHz, $CDCl_3$) δ 7.51 – 7.46 (m, 2H), 6.89 – 6.83 (m, 2H), 3.40 – 3.31 (m, 4H), 1.74 – 1.63 (m, 6H); LCMS (ESI) $[M + H]^+$ *m/z*: 187.2, retention time = 2.38 min.

3-(Piperidin-1-yl)benzonitrile (50) [20]

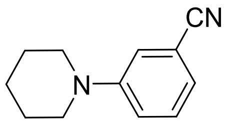

3-Fluorobenzonitrile (0.50 mL, 4.7 mmol) and piperidine (0.92 mL, 9.3 mmol) were dissolved in DMSO (3.5 mL). Potassium carbonate (0.97 g, 7.0 mmol) was added and the reaction mixture was heated up to 90 °C for 40 h. After cooling to room temperature, the reaction mixture was diluted with EtOAc (30 mL) and washed with water (2 x 25 mL). The organic layer was dried by passing through a hydrophobic frit and concentrated *in vacuo*. The residue was purified by flash column chromatography (0 – 10 % EtOAc in pet. ether 40/ 60) to afford 3-(piperidin-1-yl)benzonitrile **50** as a colourless liquid (493 mg, 57 %). TLC (EtOAc/ pet. ether 40/ 60, 1: 9, v/ v), R_f = 0.15; ^{1}H NMR (400 MHz, $CDCl_3$) δ 7.33 – 7.25 (m, 1H), 7.14 – 7.09 (m, 2H), 7.06 – 7.00 (m, 1H), 3.24 – 3.15 (m, 4H), 1.74 – 1.66 (m, 4H), 1.65 – 1.58 (m, 2H); ^{13}C NMR (125 MHz, $CDCl_3$) δ 151.7, 129.6, 121.6, 120.0, 119.4, 118.4, 112.7, 49.5, 25.3, 23.9; HRMS (*m/z*): $[M + H]^+$ calcd for $C_{12}H_{15}N_2$, 187.1235; found, 187.1237; LCMS (ESI) $[M + H]^+$ *m/z*: 187.2, retention time = 2.34 min.

3-Cyclopentyl-N-(3-(piperidin-1-yl)benzyl)propanamide (51)

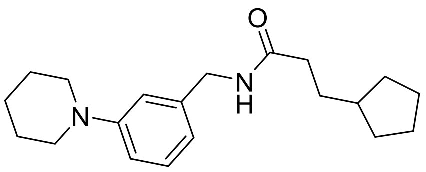

Prepared according to **general method A** using (3-(piperidin-1-yl)phenyl)methanamine **43** (50 mg, 0.26 mmol), 3-cyclopentylpropionic acid (56.3 µL, 0.39 mmol), diisopropylethylamine (229 µL, 1.3 mmol), anhydrous DCM (2.0 mL) and COMU (124 mg, 0.29 mmol). Purification by flash column chromatography (0 – 50 % EtOAc in pet. ether 40/

60) afforded the *amide* **51** as yellow oil (52.3 mg, 63 %). TLC (EtOAc/ pet. ether 40/ 60, 1: 1, v/ v), R_f = 0.29; ^{1}H NMR (400 MHz, $CDCl_3$) δ 7.26 – 7.19 (m, 1H), 6.90 – 6.83 (m, 2H), 6.77 – 6.70 (m, 1H), 5.68 (s, 1H), 4.46 – 4.36 (m, 2H), 3.23 – 3.11 (m, 4H), 2.32 – 2.17 (m, 2H), 1.85 – 1.45 (m, 15H), 1.20 – 1.02 (m, 2H); ^{13}C NMR (125 MHz, $CDCl_3$) δ 173.0, 152.6, 139.1, 129.4, 118.5, 116.0, 115.5, 50.5, 44.1, 39.8, 36.2, 32.5, 32.0, 25.8, 25.1, 24.3; IR, ν_{max} (ATR): 3266, 3085 (w), 2935, 2855, 2801, 1638, 1599 cm^{-1}; HRMS (*m/z*): $[M + H]^+$ calcd for $C_{20}H_{31}N_2O$, 315.2436; found, 315.2429.

3-Cyclopentyl-N-(4-(piperidin-1-yl)benzyl)propanamide (52)

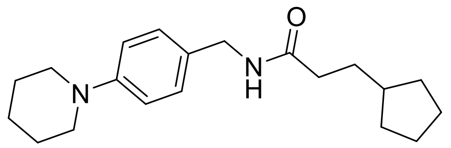

Prepared according to **general method A** using (4-(piperidin-1-yl)phenyl)methanamine **46** (50 mg, 0.26 mmol), 3-cyclopentylpropionic acid (37.6 µL, 0.26 mmol), diisopropylethylamine (229 µL, 1.3 mmol), anhydrous DCM (2.5 mL) and COMU (124 mg, 0.29 mmol). Purification by flash column chromatography (0 – 100 % EtOAc in pet. ether 40/ 60) afforded the *amide* **52** as white crystalline solid (61 mg, 74 %). TLC (100 % EtOAc), R_f = 0.53; ^{1}H NMR (400 MHz, $CDCl_3$) δ 7.21 – 7.13 (m, 2H), 6.95 – 6.86 (m, 2H), 5.68 (s, 1H), 4.39 – 4.30 (m, 2H), 3.21 – 3.10 (m, 4H), 2.28 – 2.15 (m, 2H), 1.87 – 1.45 (m, 15H), 1.19 – 1.04 (m, 2H); ^{13}C NMR (125 MHz, $CDCl_3$) δ 172.9, 151.6, 128.8, 116.5, 50.6, 43.2, 39.7, 36.1, 32.4, 32.0, 25.7, 25.1, 24.2; HRMS (*m/z*): $[M + H]^+$ calcd for $C_{20}H_{31}N_2O$, 315.2436; found, 315.2433; LCMS (ESI) $[M + H]^+$ *m/z*: 315.4, retention time = 1.64 min.

Selected Spectra

^{1}H NMR

3-Cyclopentyl-1-(3-(4-((methylamino)methyl)phenyl)tetrahydropyrimidin-1(2*H*)-yl)propan-1-one (3)

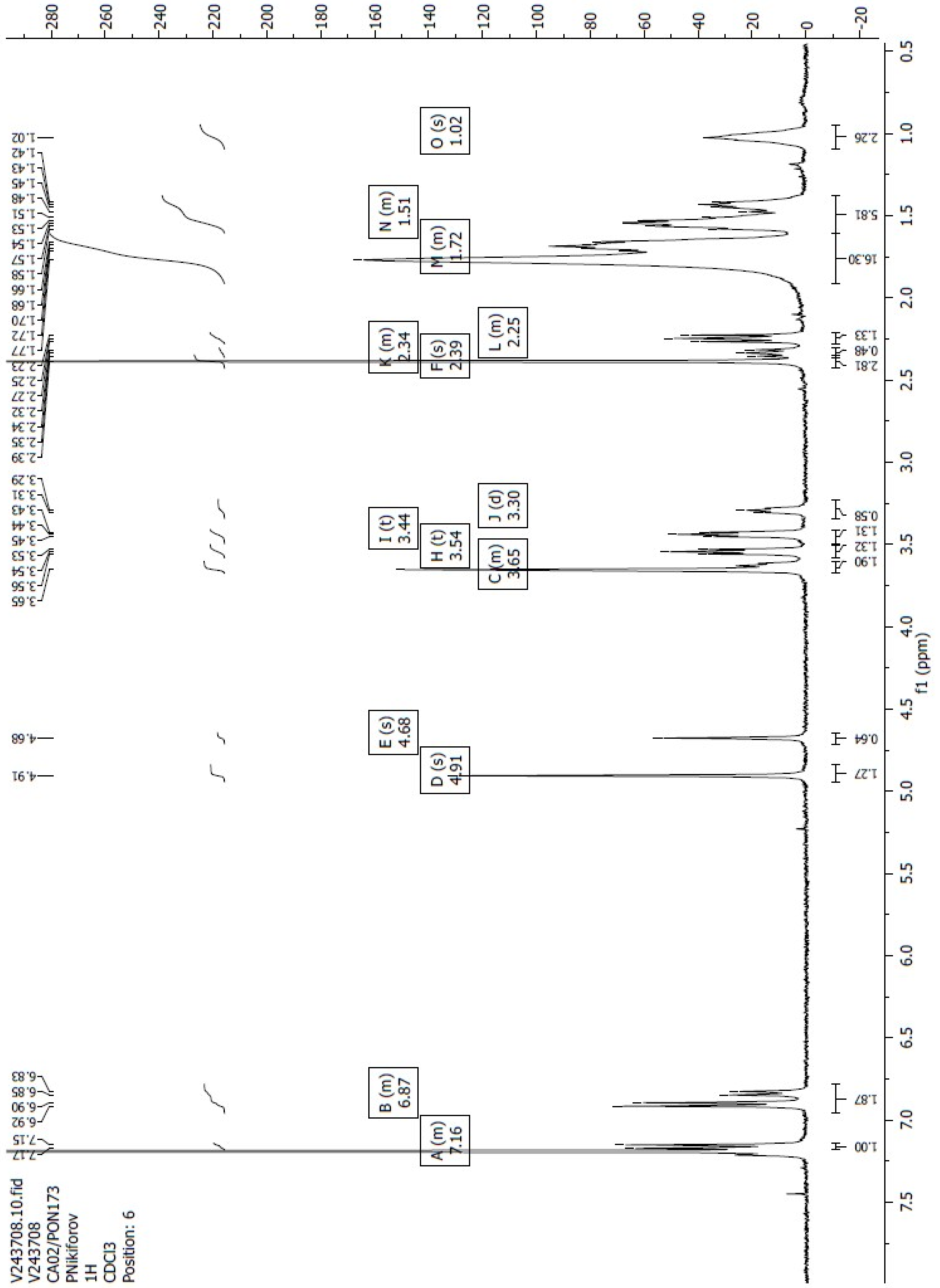

3-Cyclopentyl-N-(3-(piperidin-1-yl)phenyl)propanamide (4)

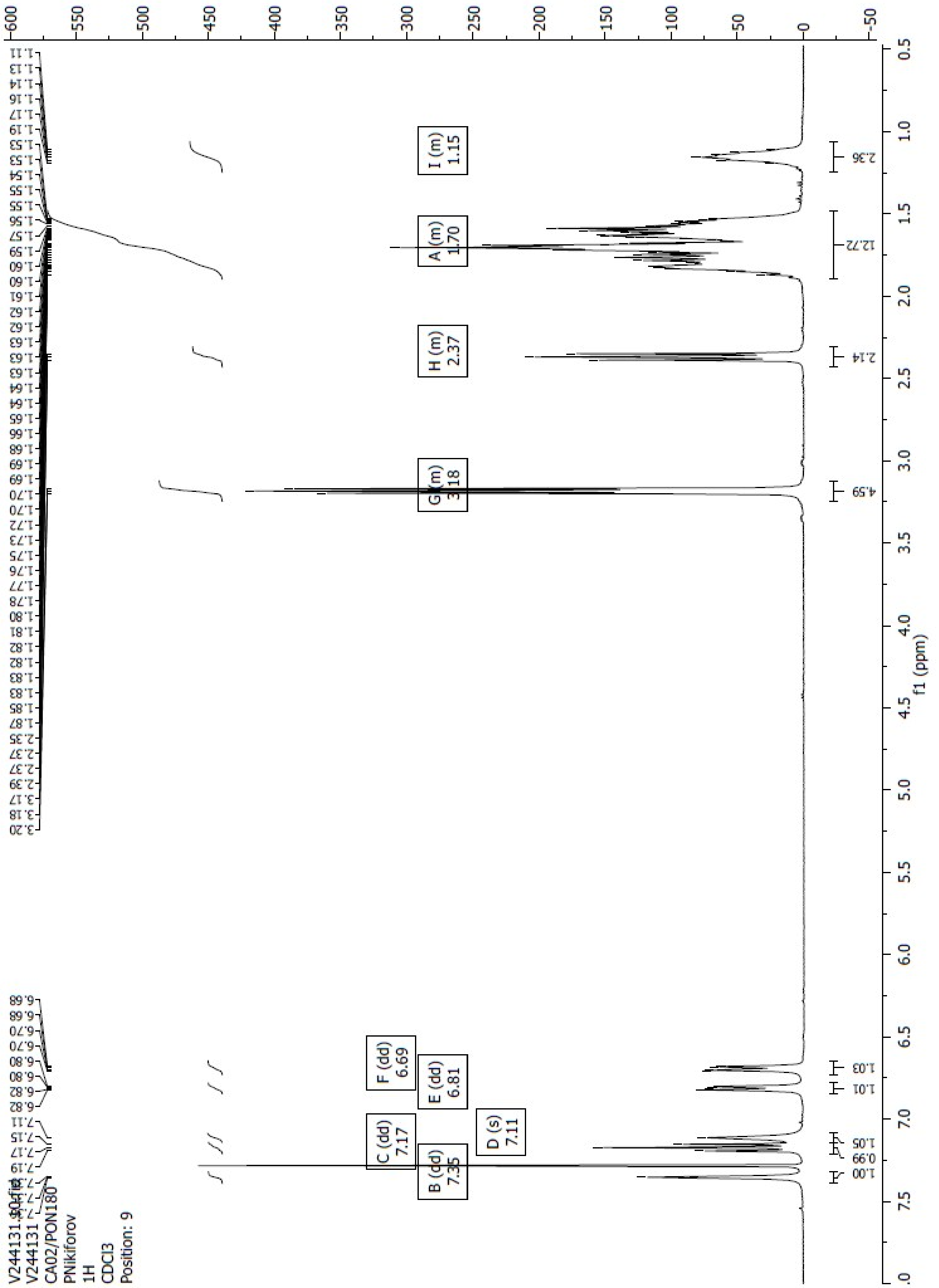

3-(1-(4-((Methylamino)methyl)phenyl)piperidin-4-yl)-1-(pyrrolidin-1-yl)propan-1-one (5)

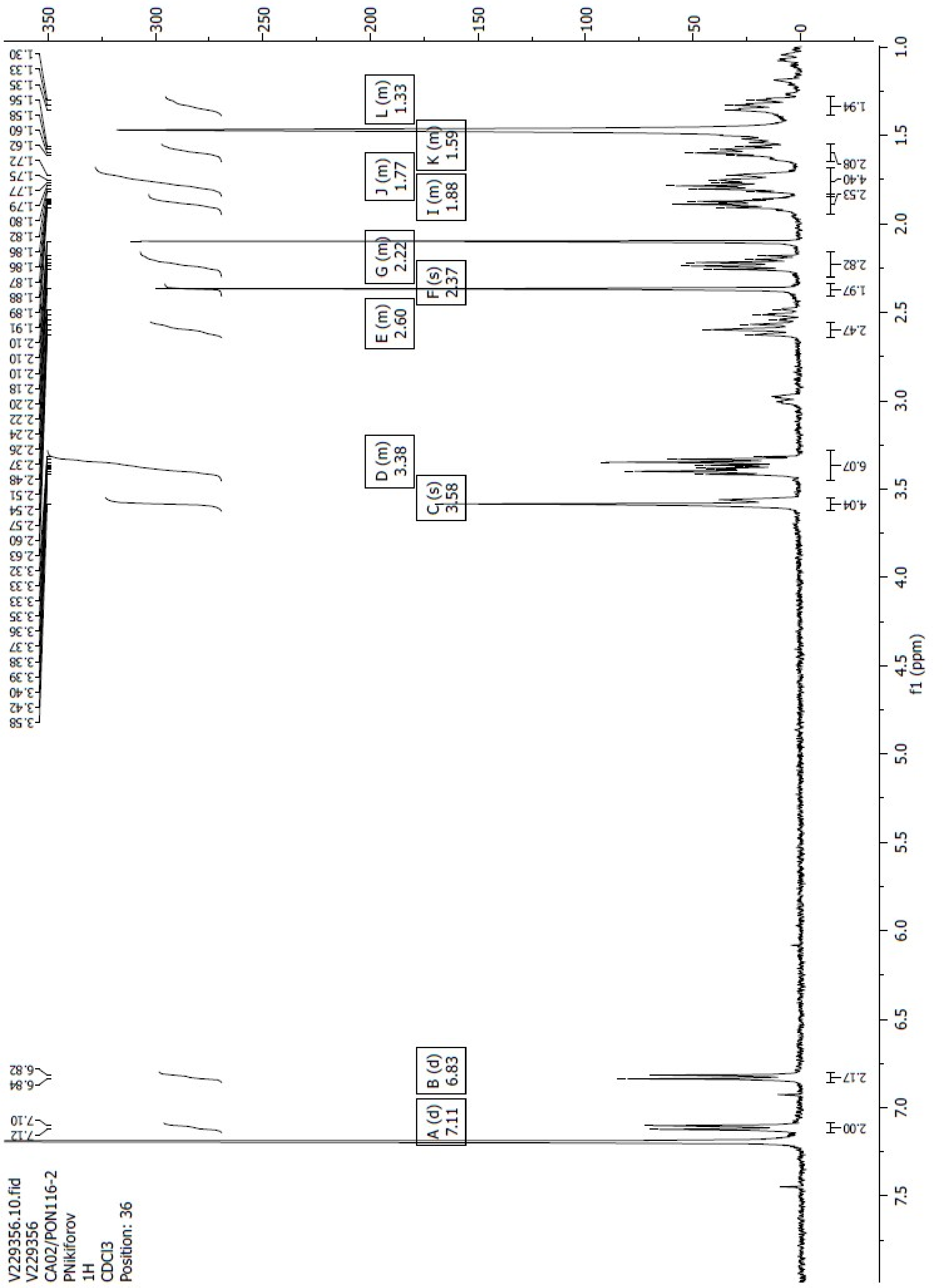

4-(4-(3-Oxo-3-(pyrrolidin-1-yl)propyl)piperidin-1-yl)benzonitrile (15)

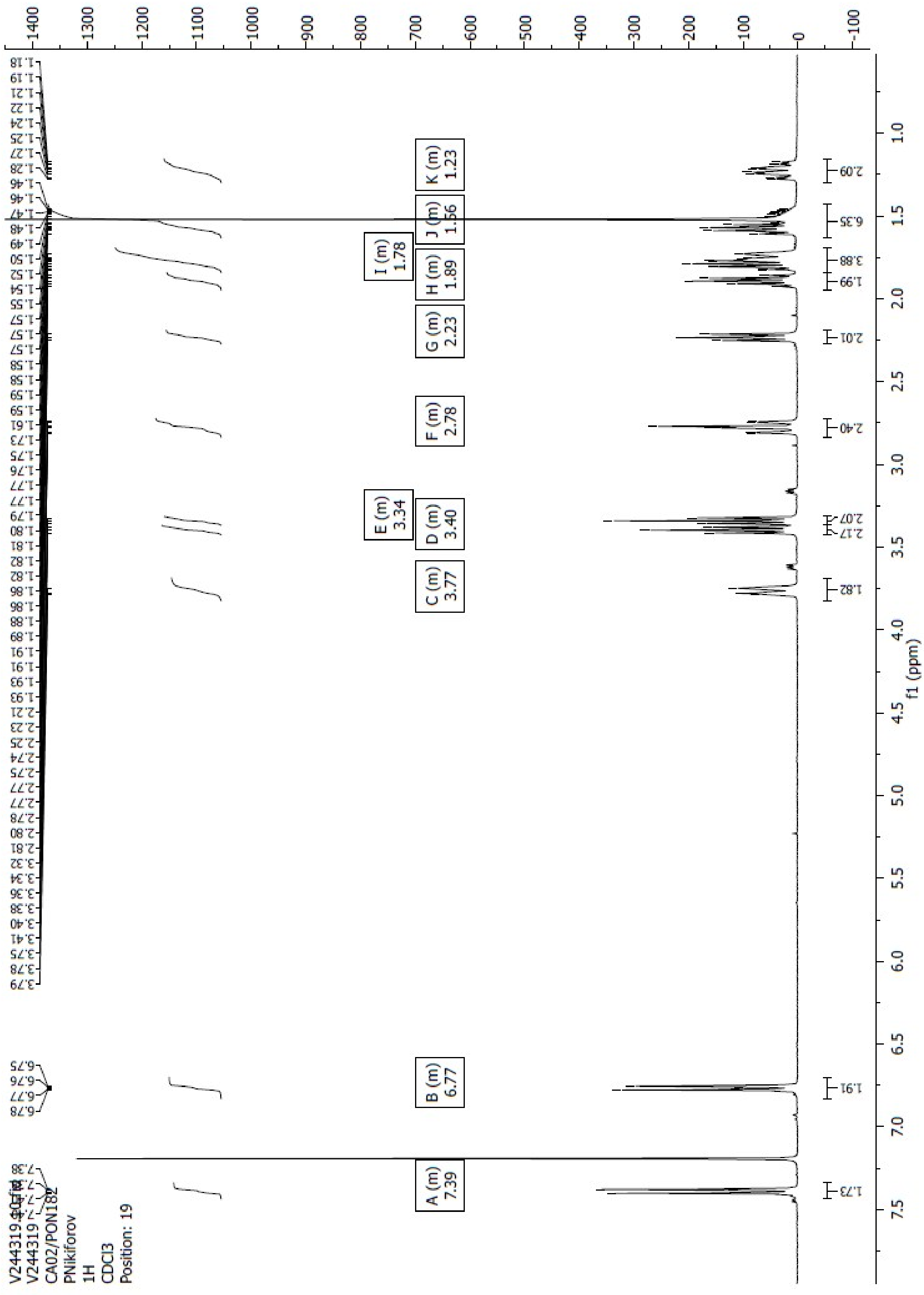

Ethyl 4-(4-(3-oxo-3-(pyrrolidin-1-yl)propyl)piperidin-1-yl)benzoate (16)

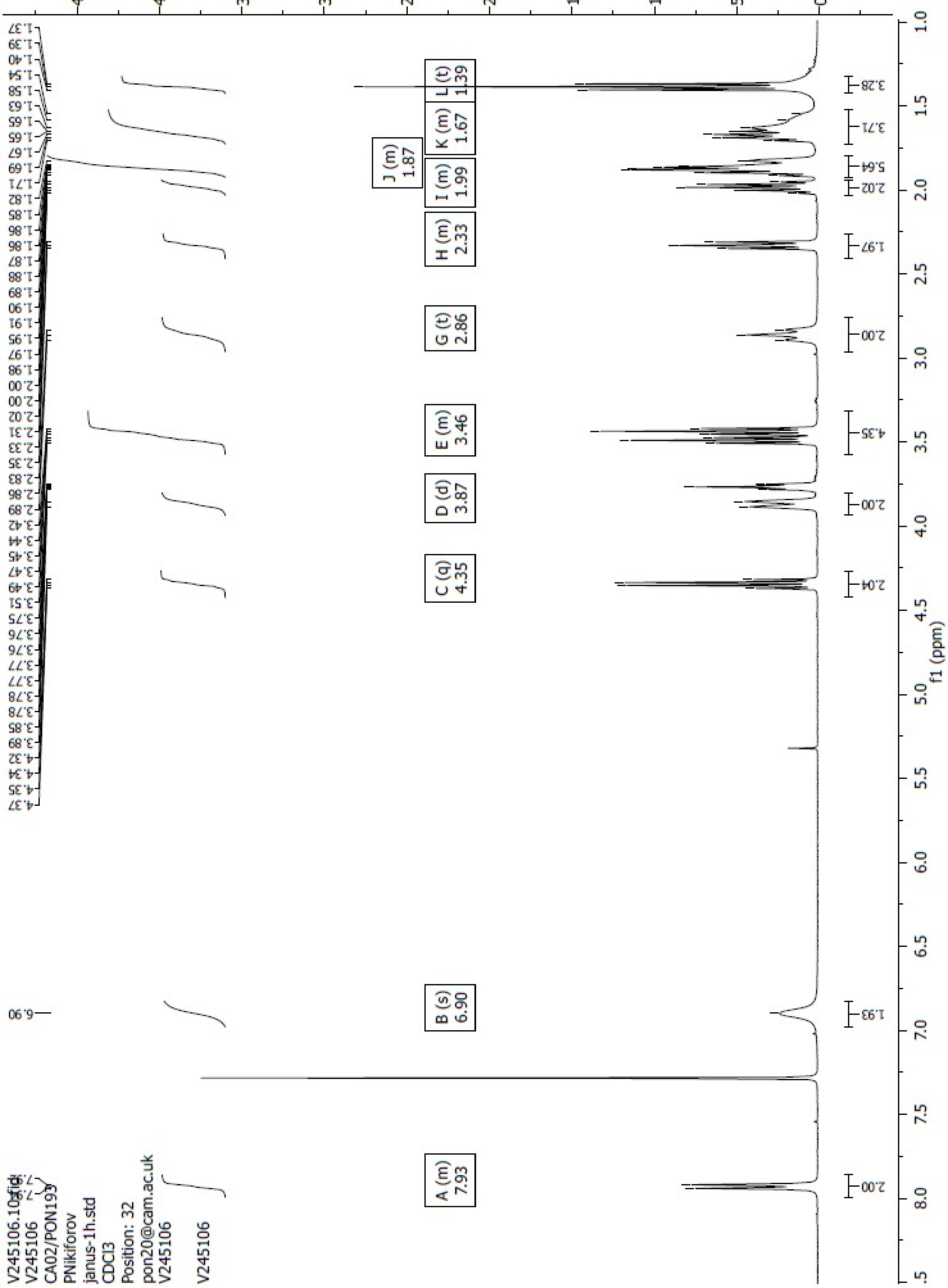

***N*-((1-(4-Cyanophenyl)piperidin-4-yl)methyl)pyrrolidine-1-carboxamide (17)**

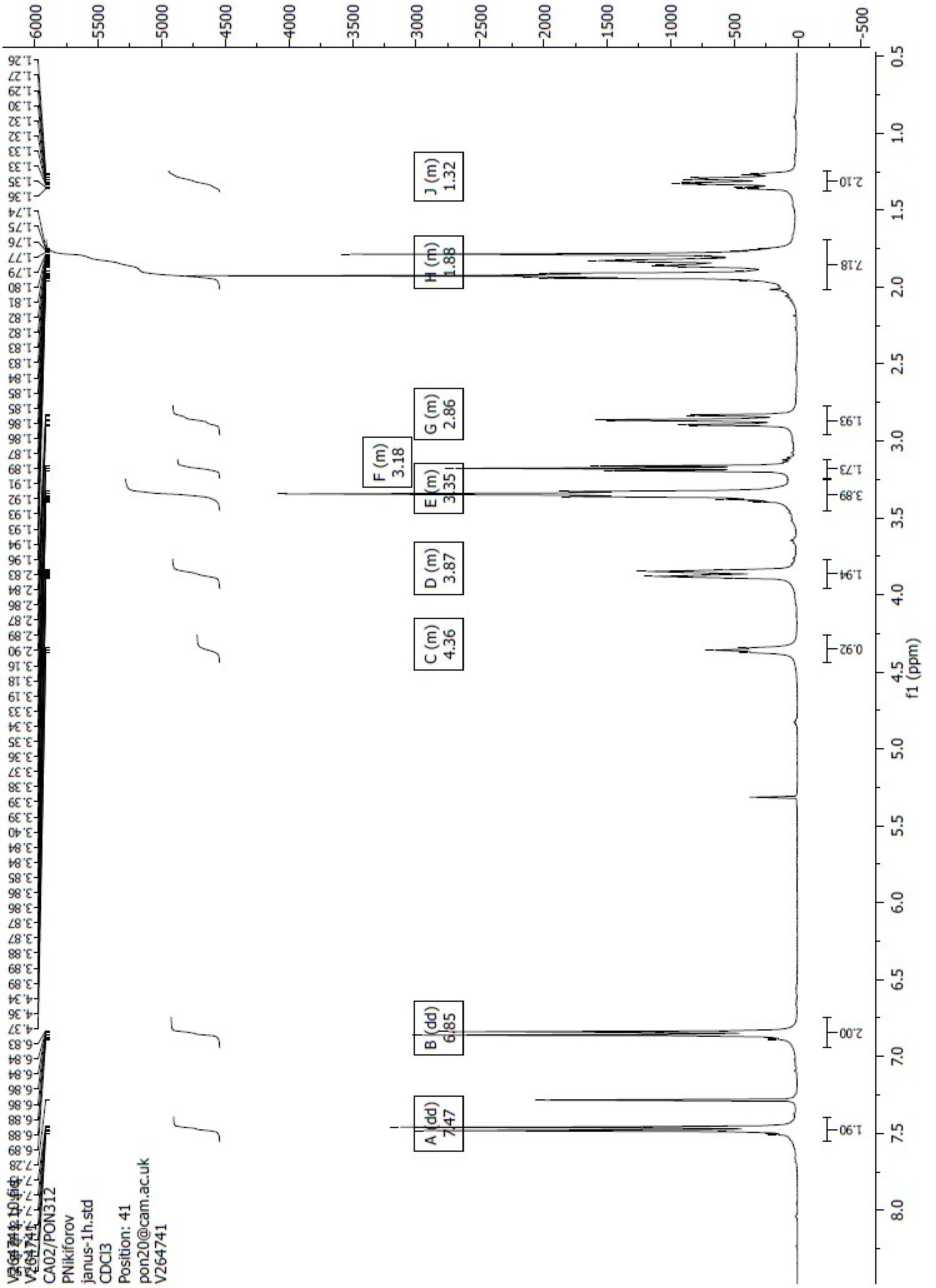

Ethyl 4-(4-((pyrrolidine-1-carboxamido)methyl)piperidin-1-yl)benzoate (18)

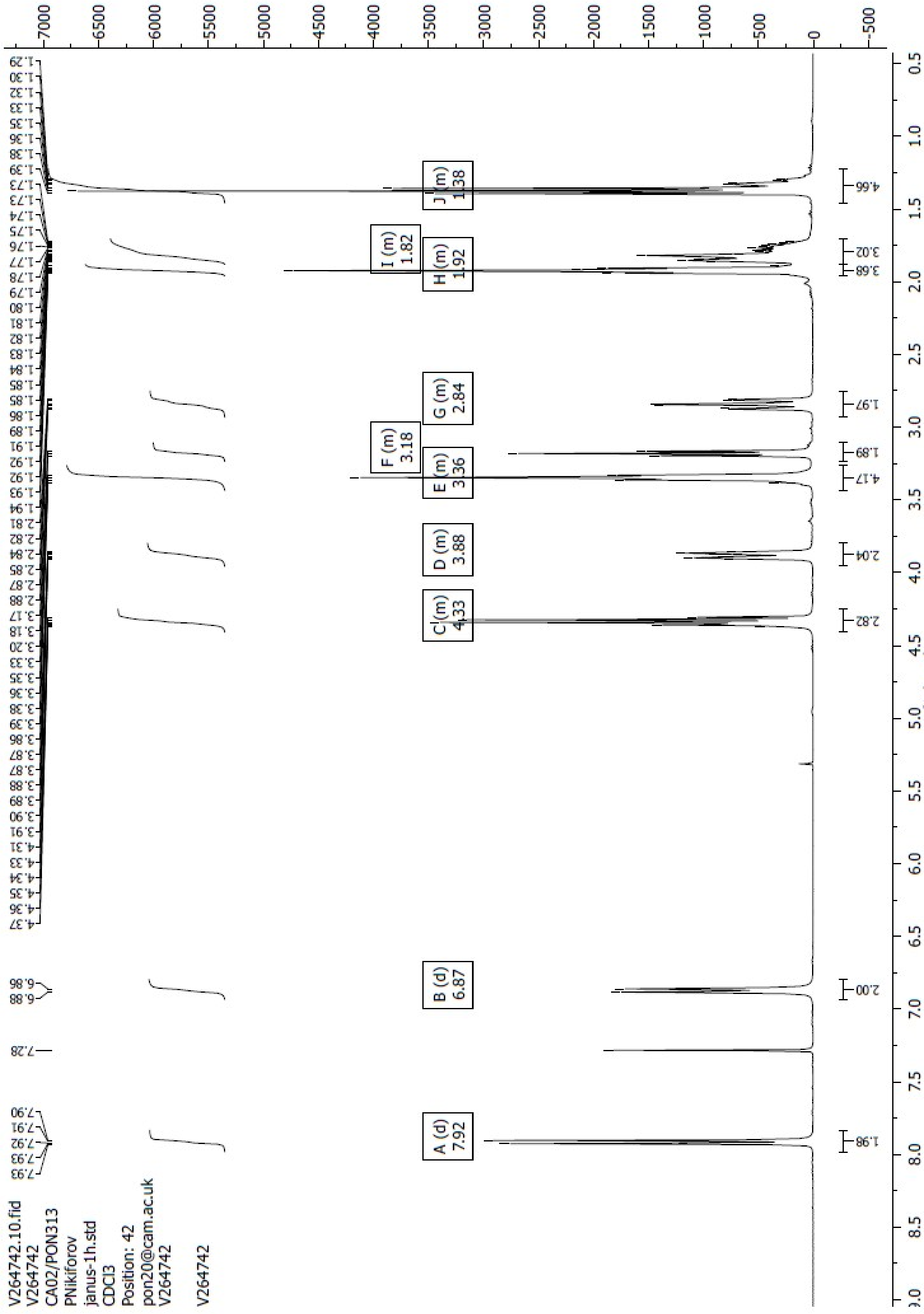

***N*-((1-(4-Cyanophenyl)piperidin-4-yl)methyl)cyclopentanecarboxamide (19)**

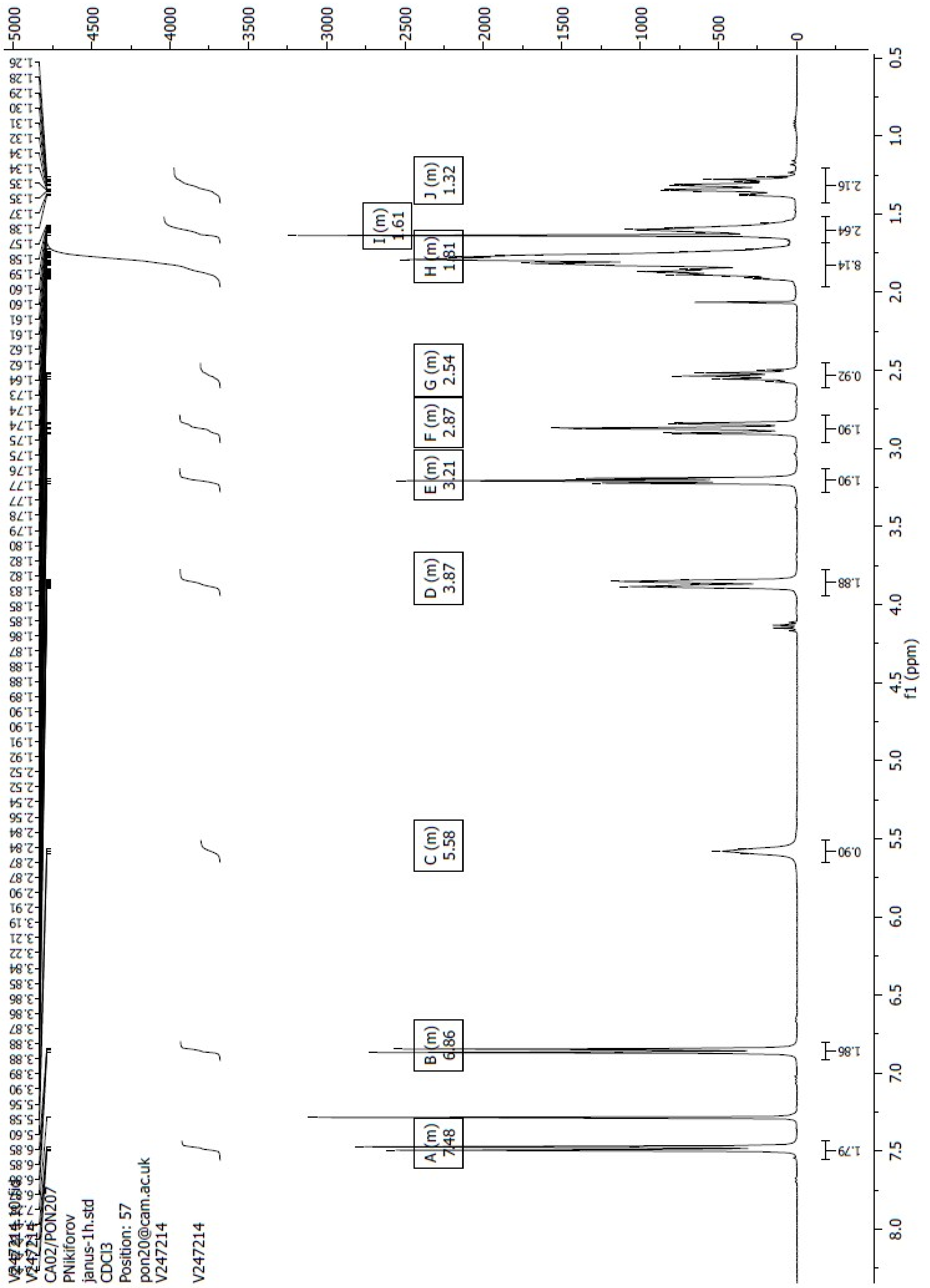

Ethyl 4-(4-(cyclopentanecarboxamidomethyl)piperidin-1-yl)benzoate (20)

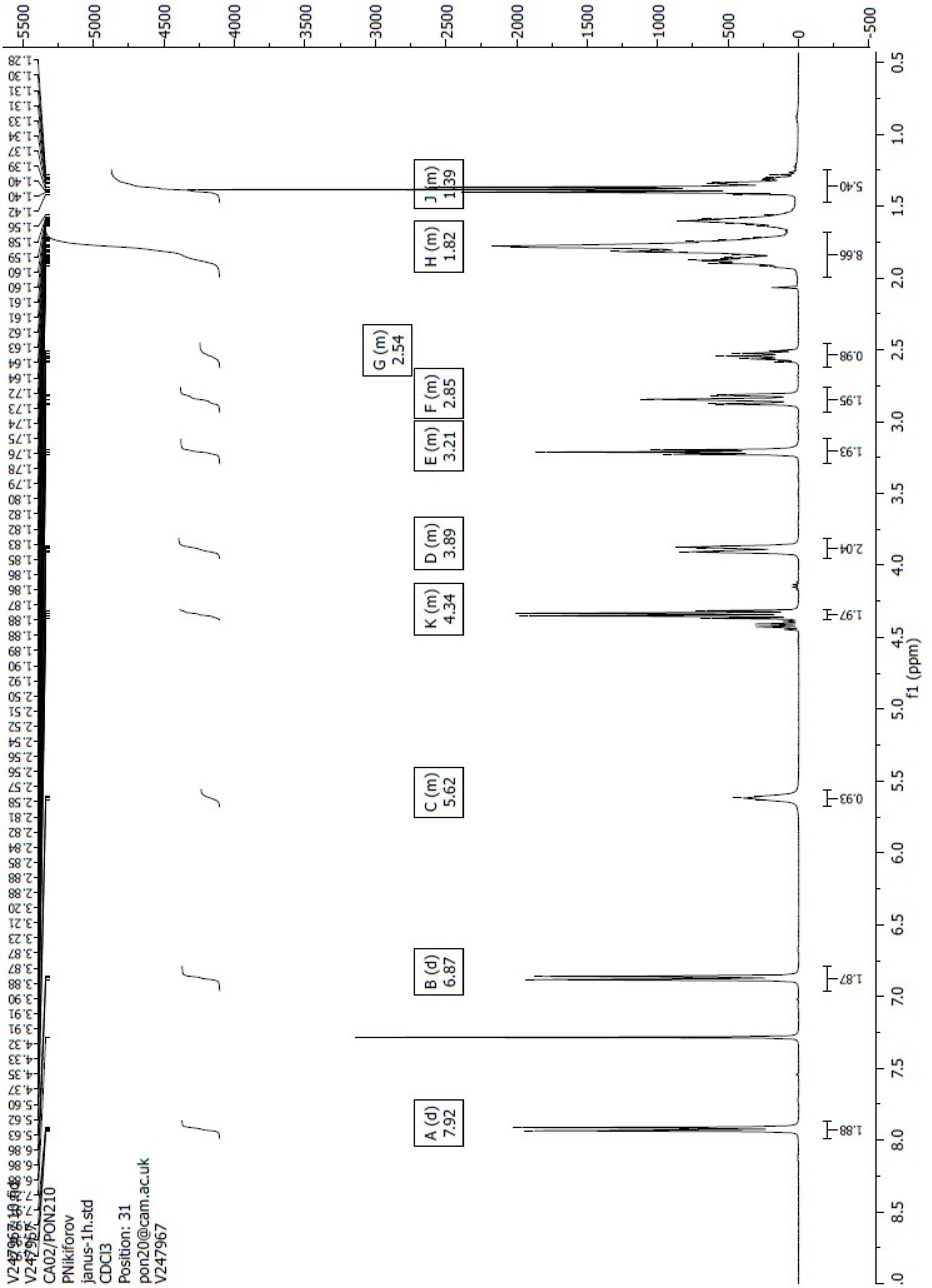

N-((1-(Pyridin-2-yl)piperidin-4-yl)methyl)pyrrolidine-1-carboxamide (21)

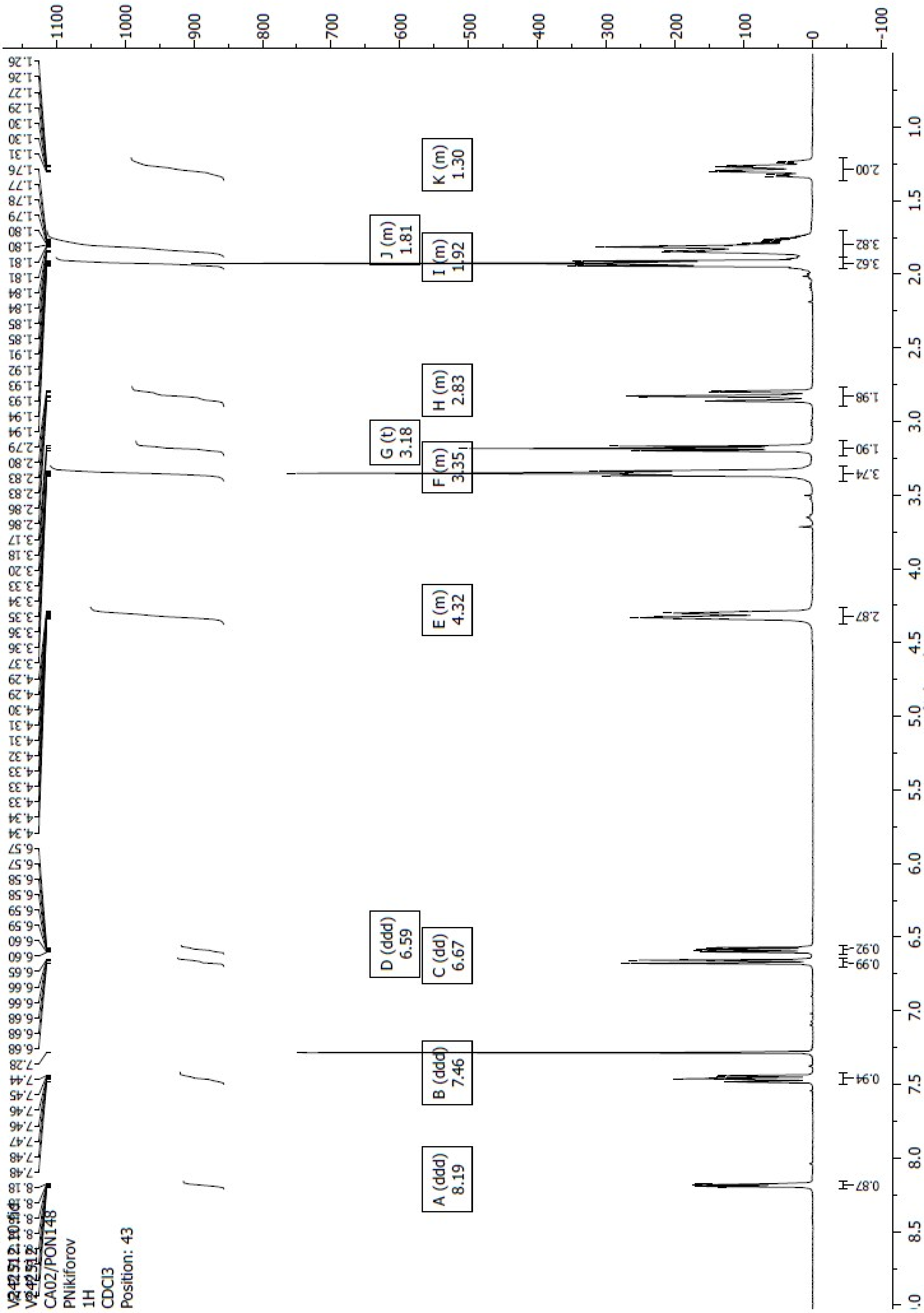

_N_-((1-(Pyrimidin-2-yl)piperidin-4-yl)methyl)pyrrolidine-1-carboxamide (22)

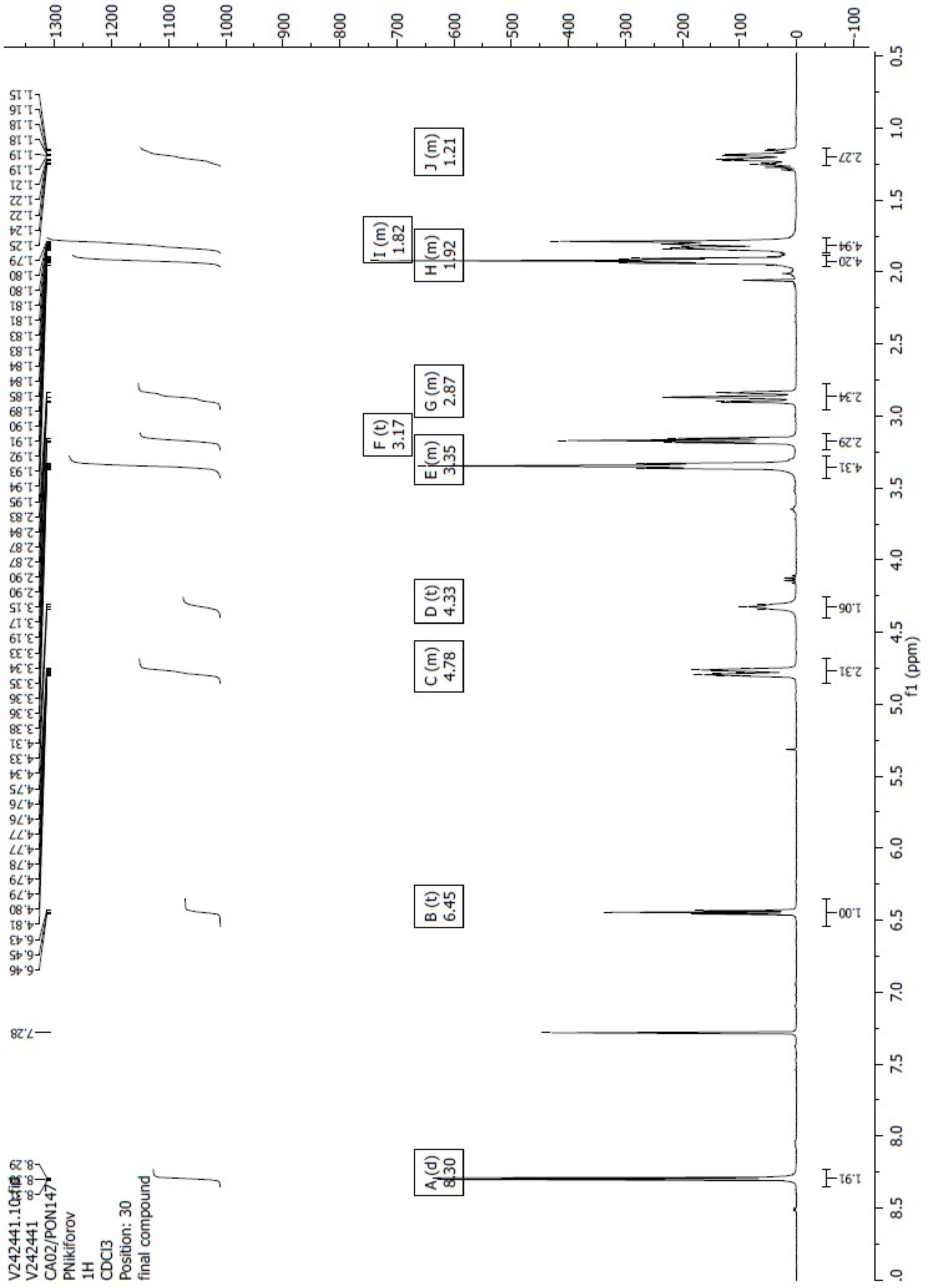

3-(1-(4-Cyanophenyl)piperidin-4-yl)-N-(3-(piperidin-1-yl)phenyl)propanamide (23)

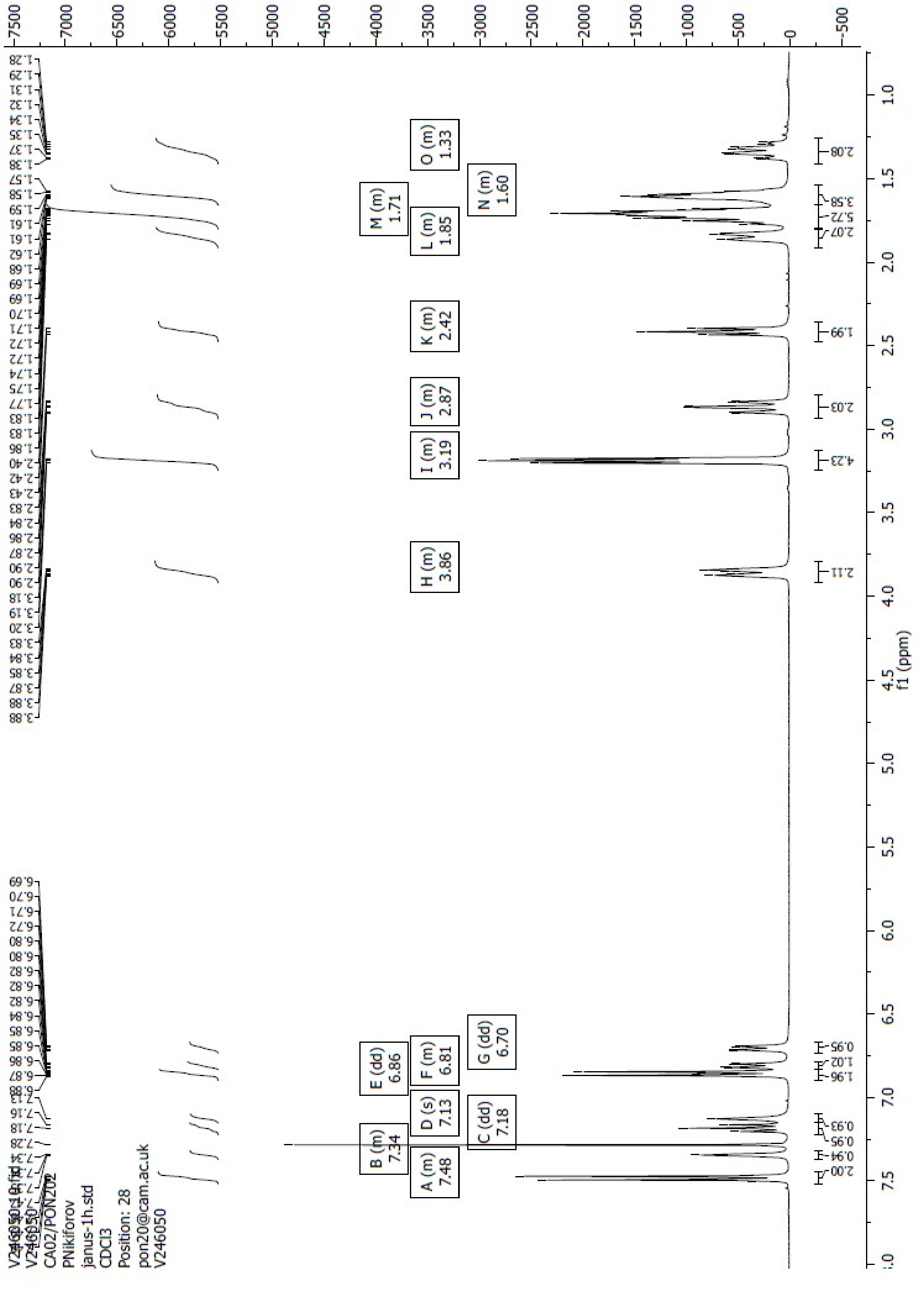

3-(1-(4-Cyanophenyl)piperidin-4-yl)-N-(4-(piperidin-1-yl)benzyl)propanamide (28)

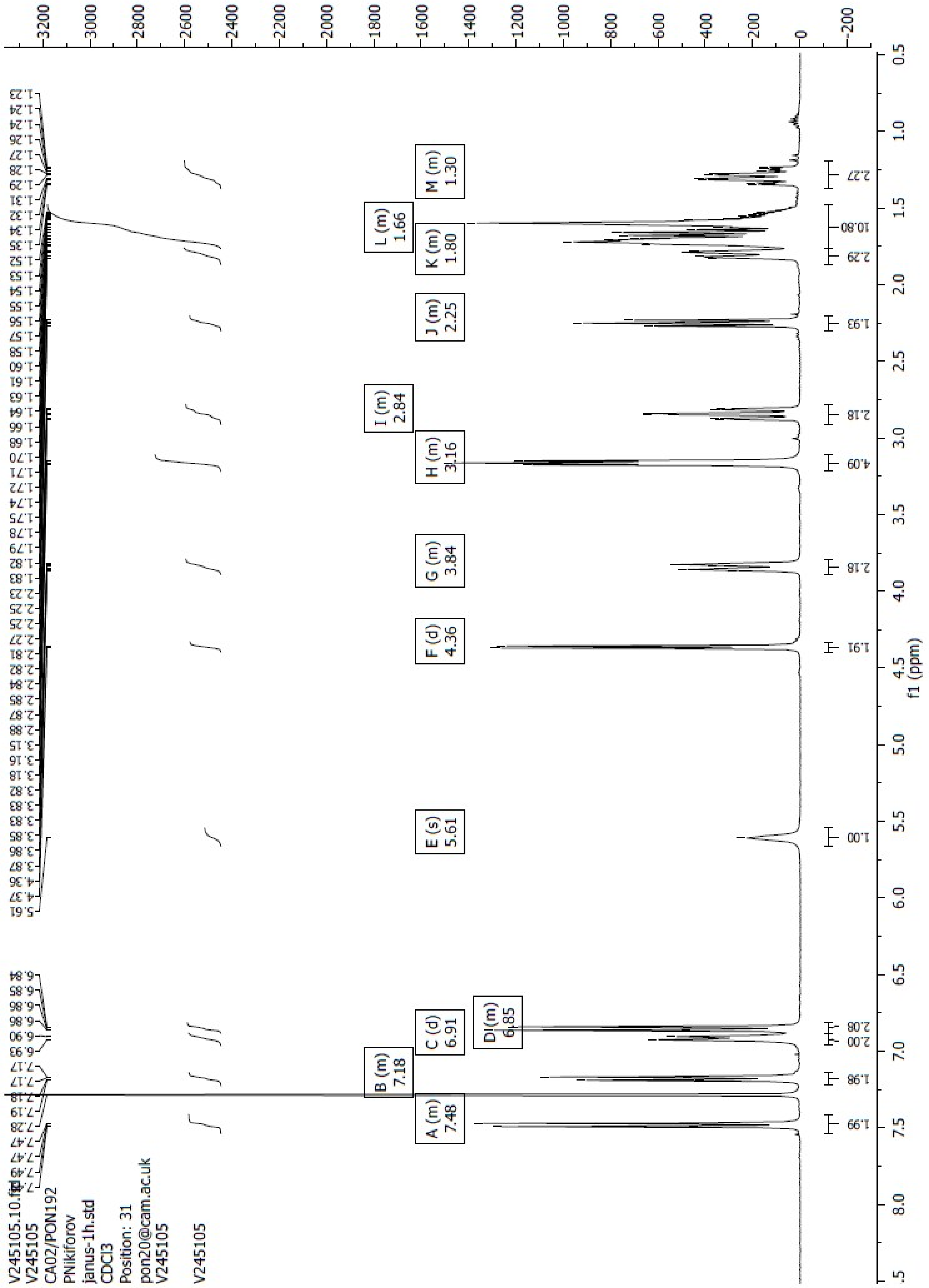

3-(1-(4-Cyanophenyl)piperidin-4-yl)-N-(3-(piperidin-1-yl)benzyl)propanamide (29)

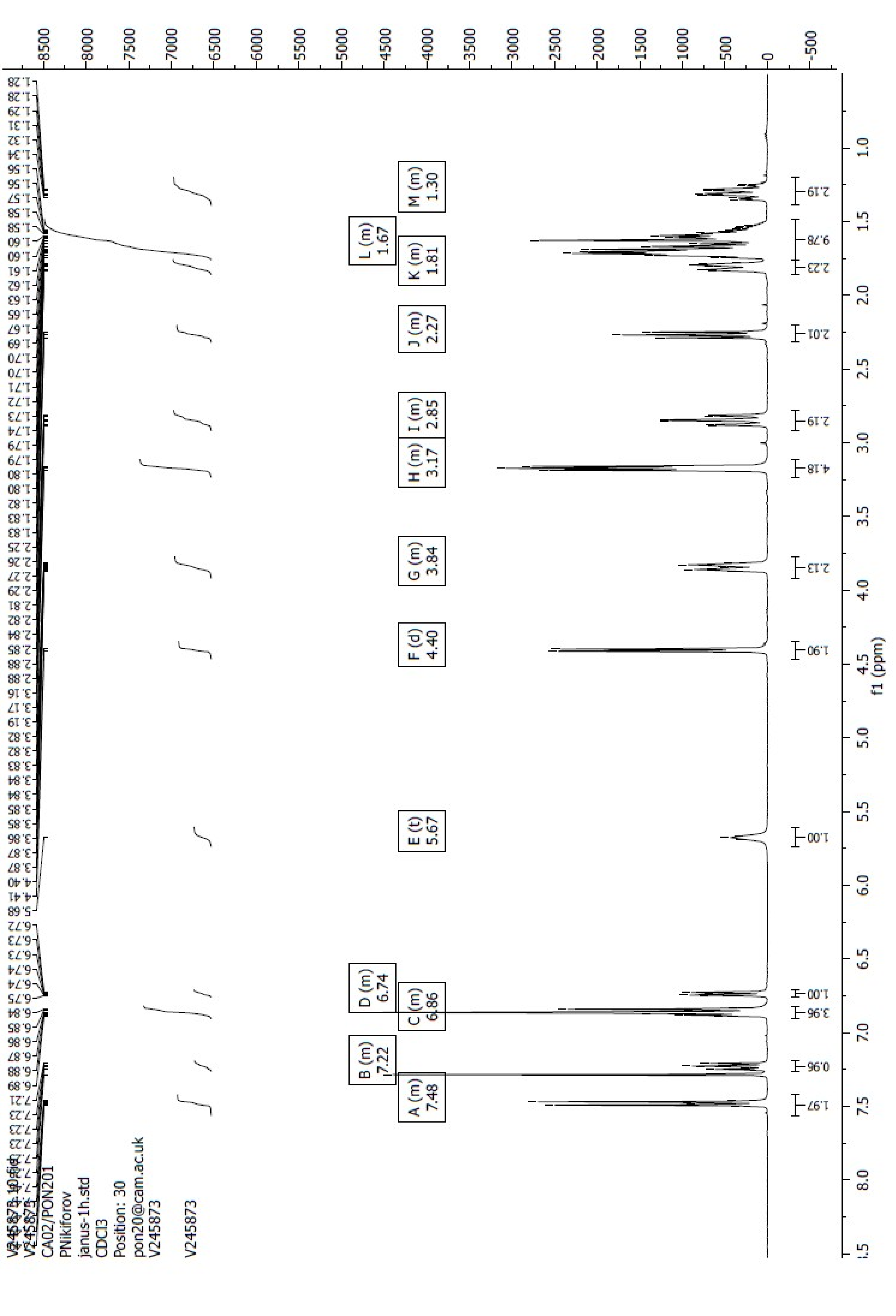

^{13}C NMR

3-Cyclopentyl-1-(3-(4-((methylamino)methyl)phenyl)tetrahydropyrimidin-1(2*H*)-yl)propan-1-one (3)

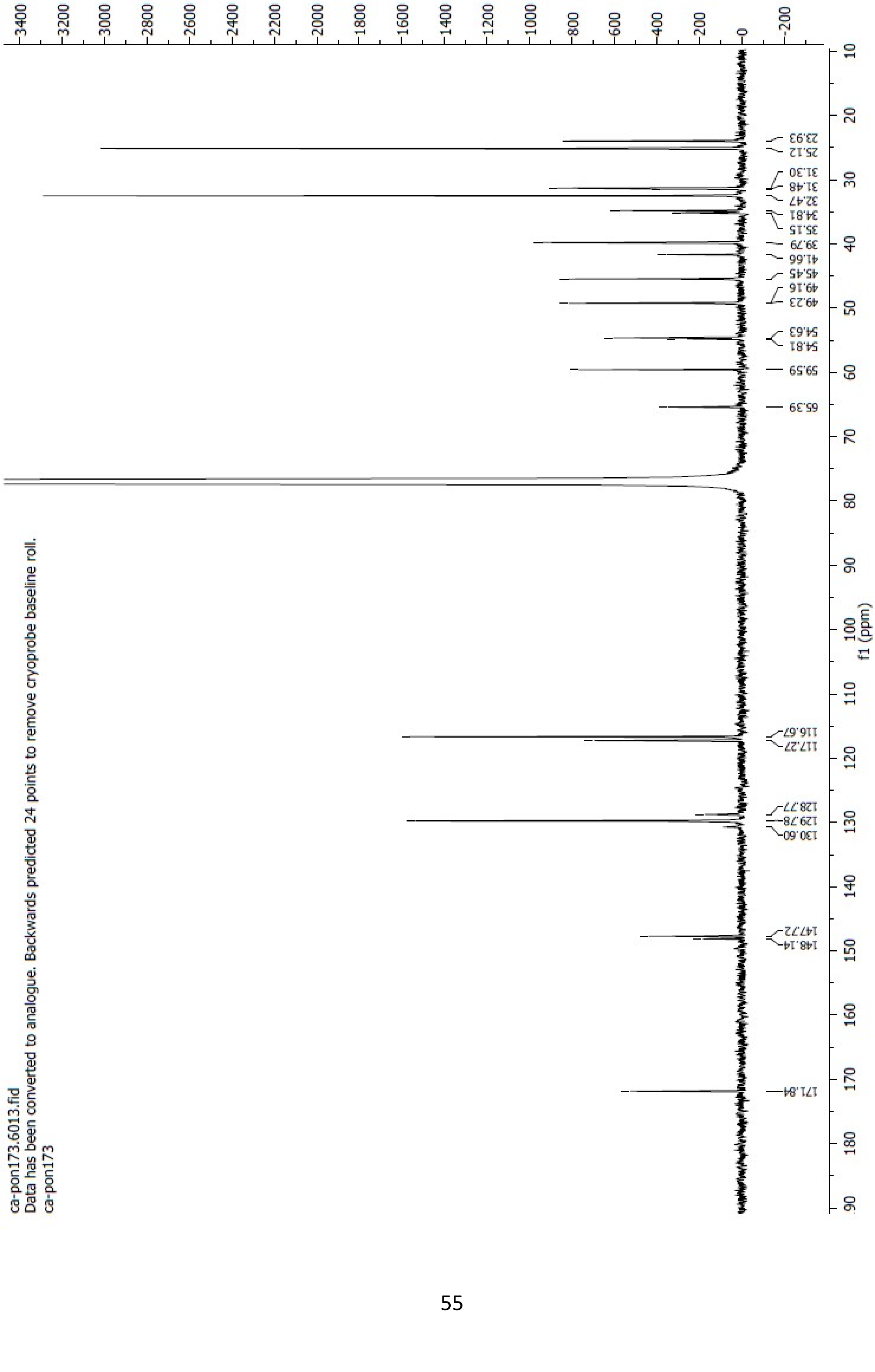

3-Cyclopentyl-N-(3-(piperidin-1-yl)phenyl)propanamide (4)

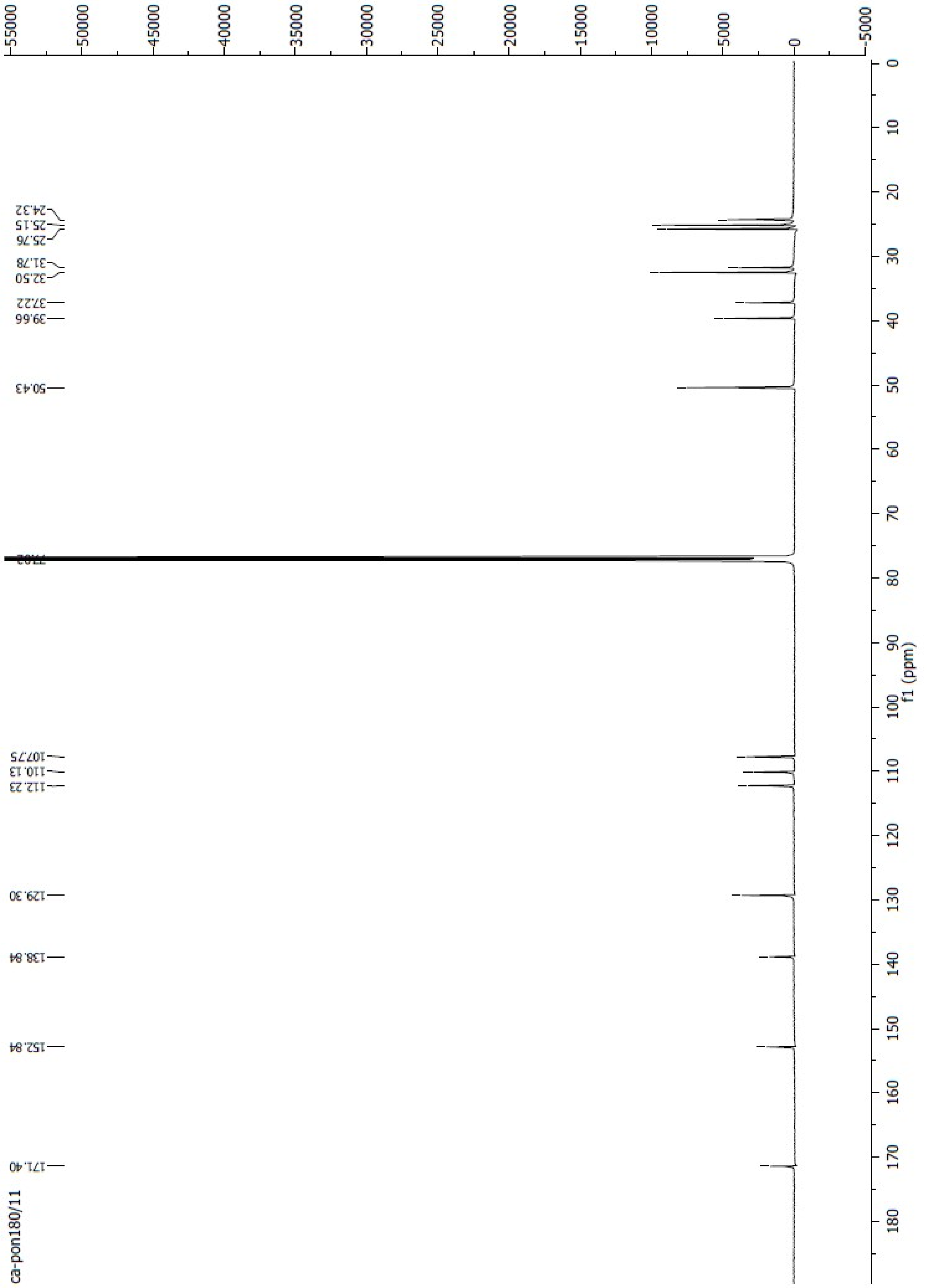

4-(4-(3-Oxo-3-(pyrrolidin-1-yl)propyl)piperidin-1-yl)benzonitrile (15)

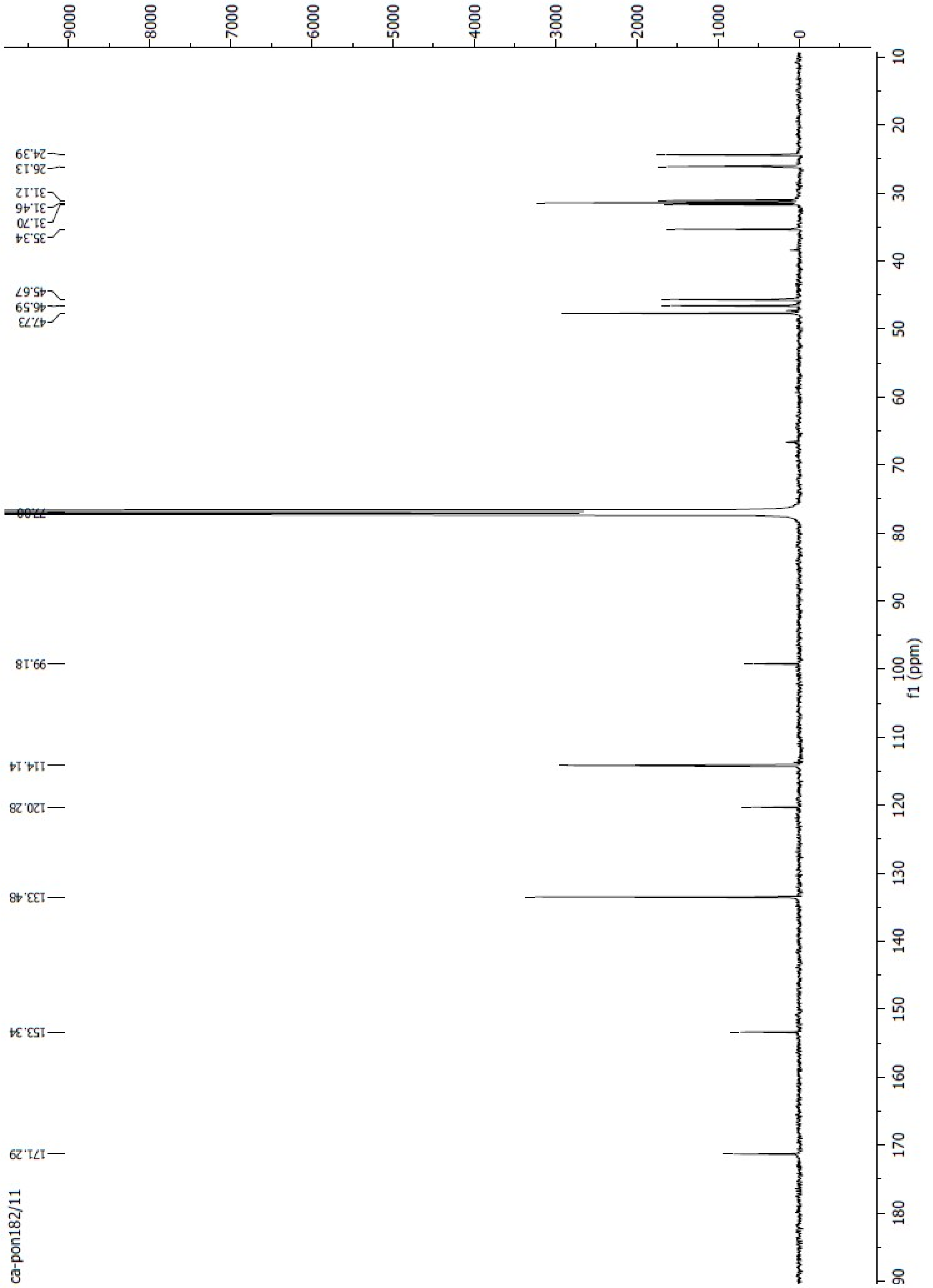

Ethyl 4-(4-(3-oxo-3-(pyrrolidin-1-yl)propyl)piperidin-1-yl)benzoate (16)

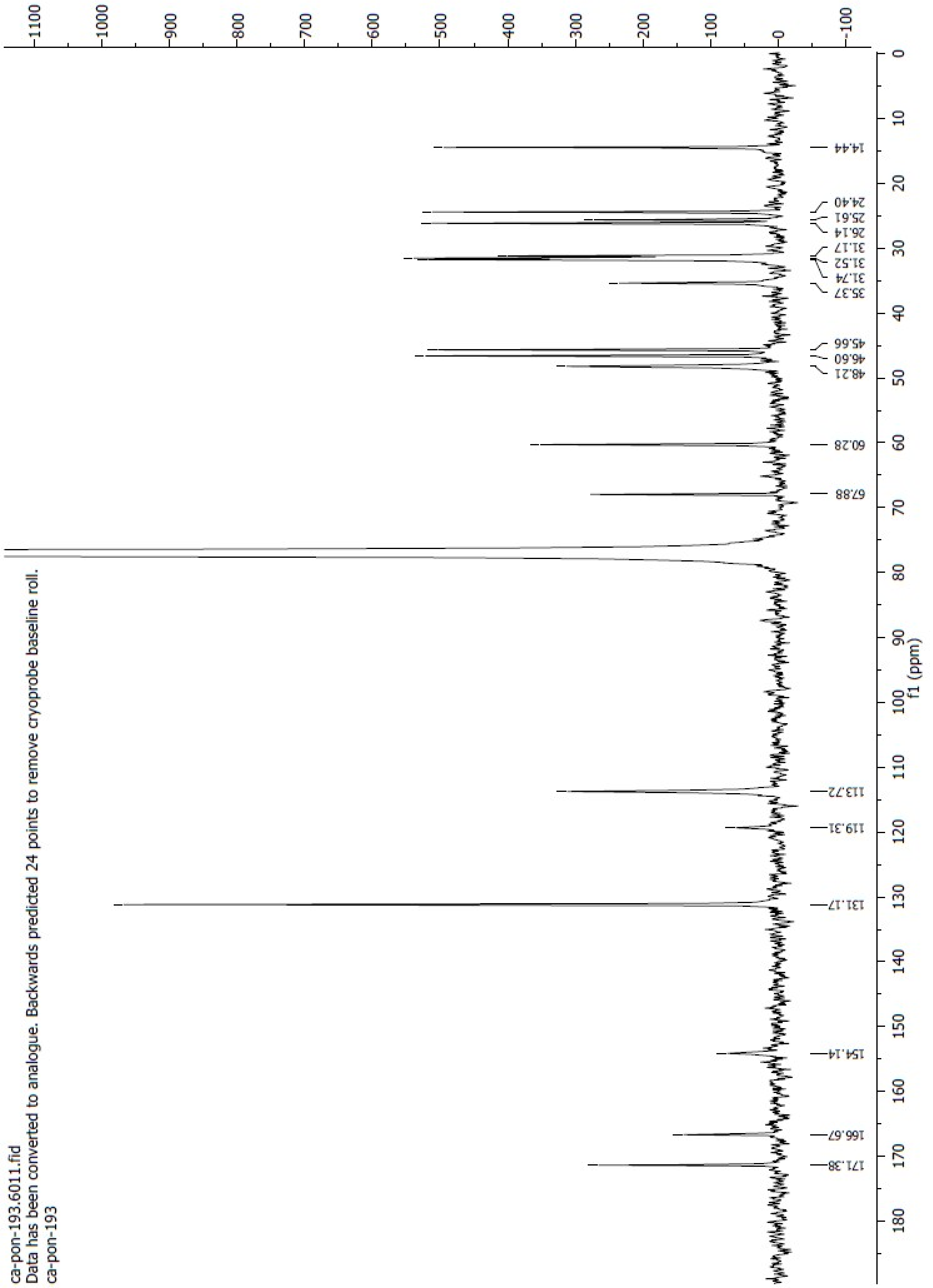

N-((1-(4-Cyanophenyl)piperidin-4-yl)methyl)pyrrolidine-1-carboxamide (17)

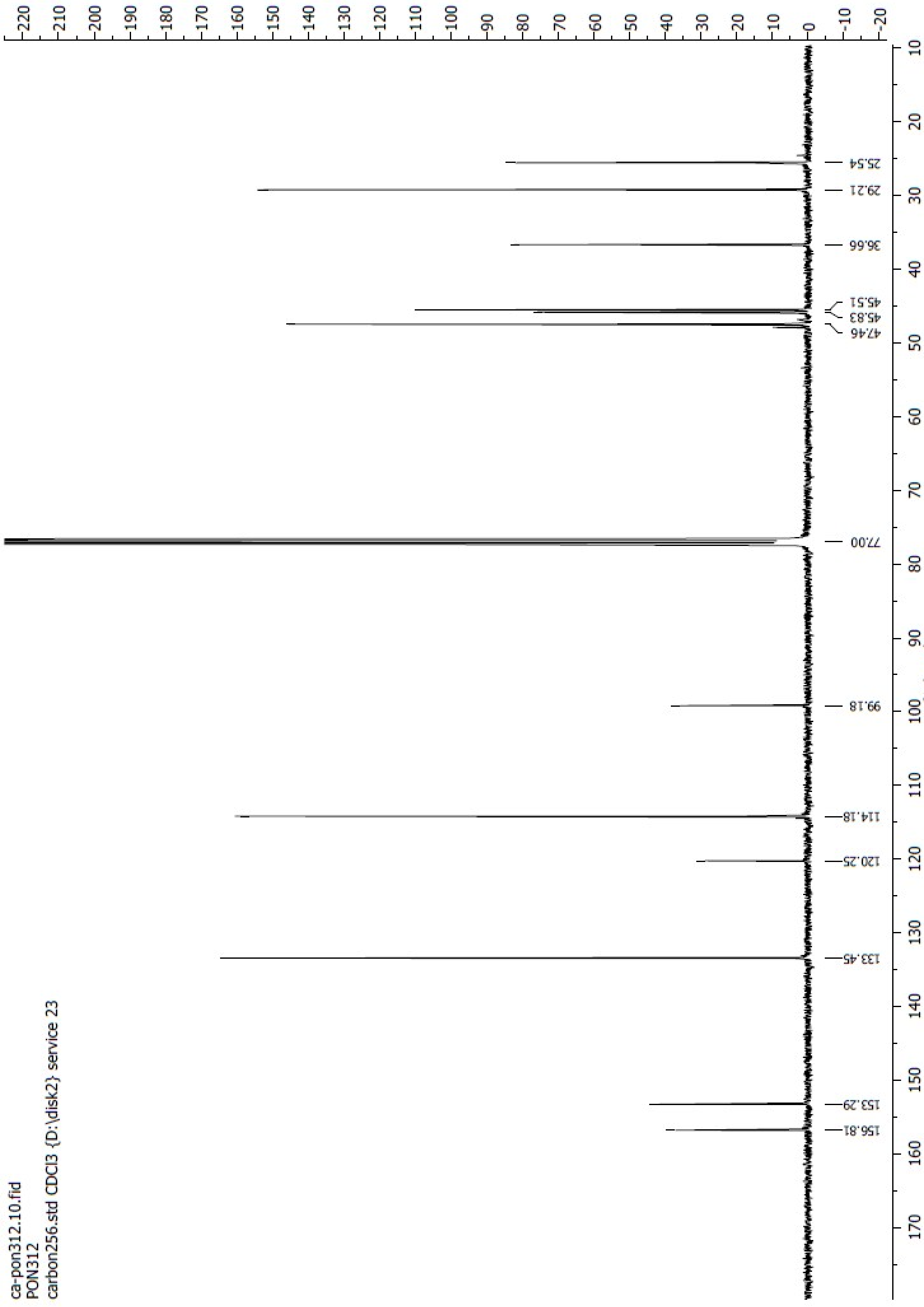

Ethyl 4-(4-((pyrrolidine-1-carboxamido)methyl)piperidin-1-yl)benzoate (18)

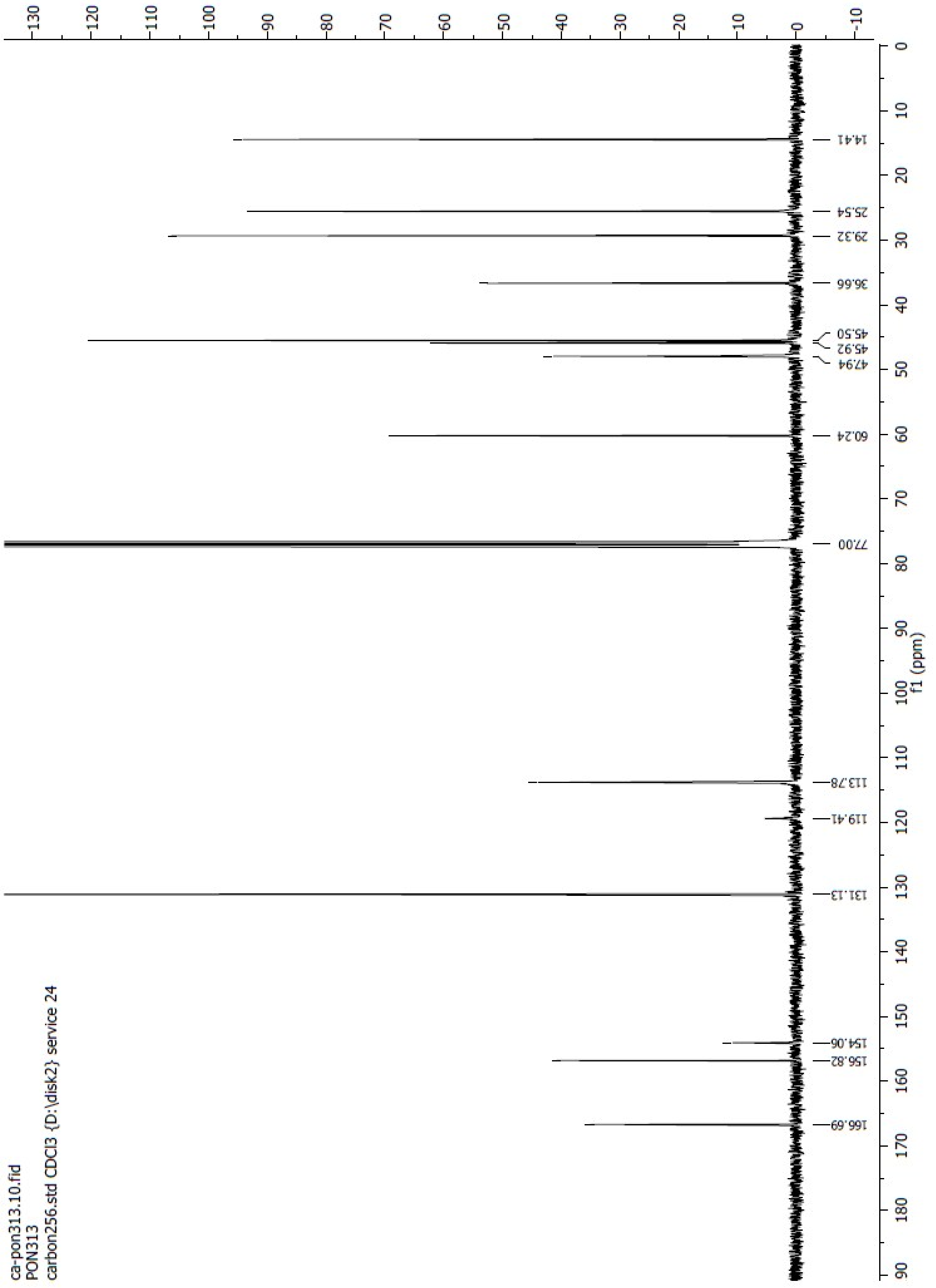

***N*-((1-(4-Cyanophenyl)piperidin-4-yl)methyl)cyclopentanecarboxamide (19)**

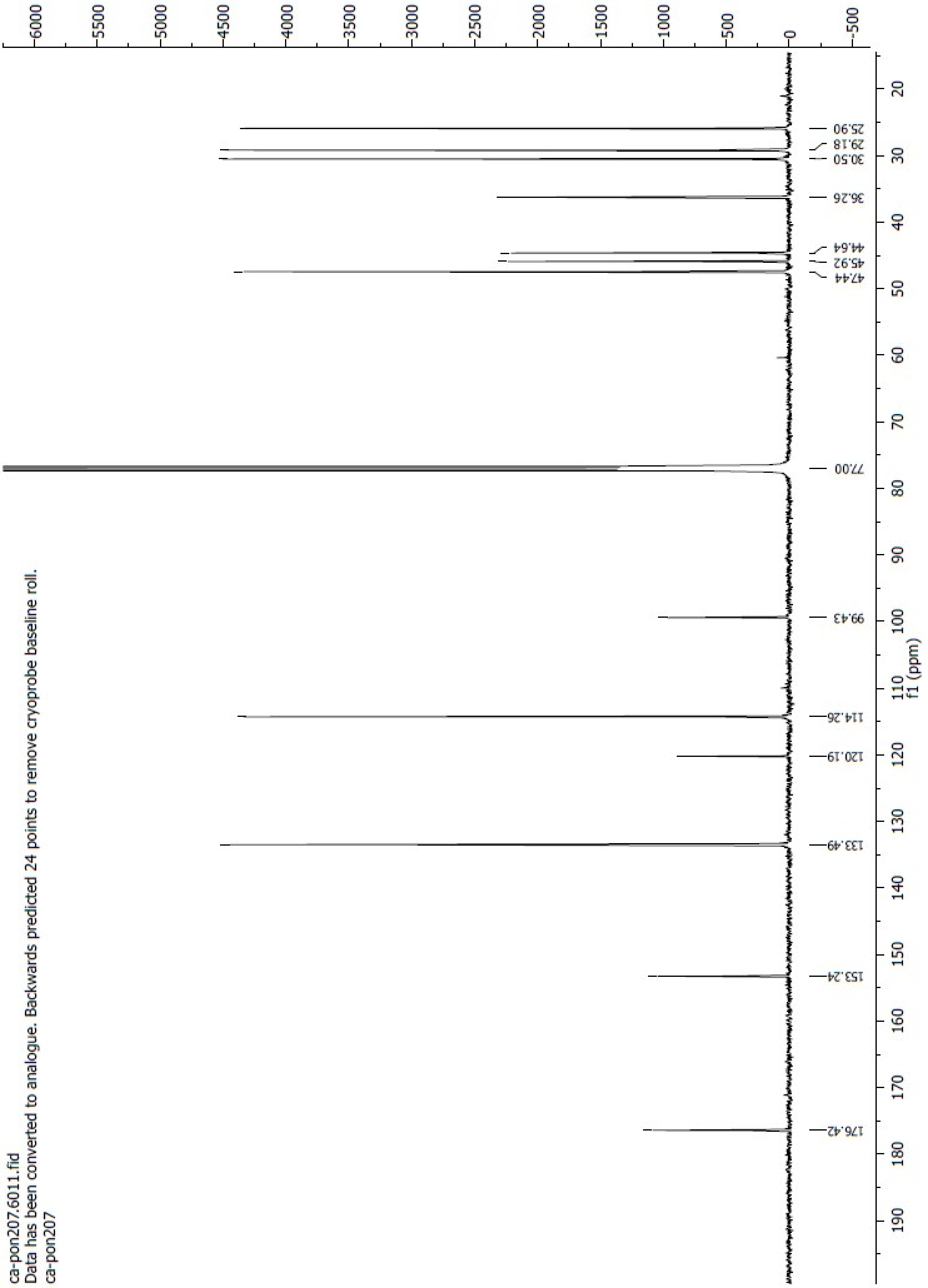

Ethyl 4-(4-(cyclopentanecarboxamidomethyl)piperidin-1-yl)benzoate (20)

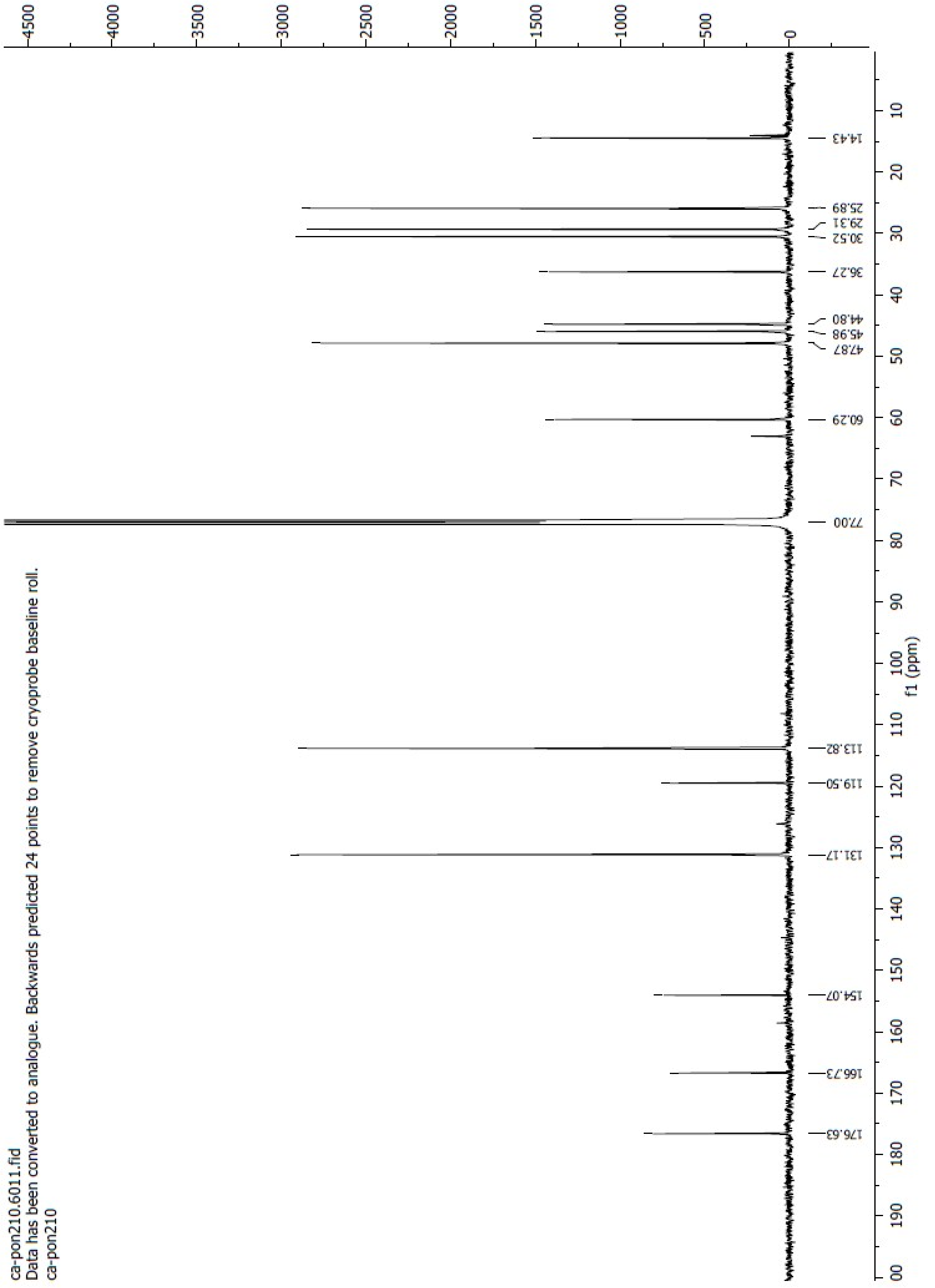

N-((1-(Pyridin-2-yl)piperidin-4-yl)methyl)pyrrolidine-1-carboxamide (21)

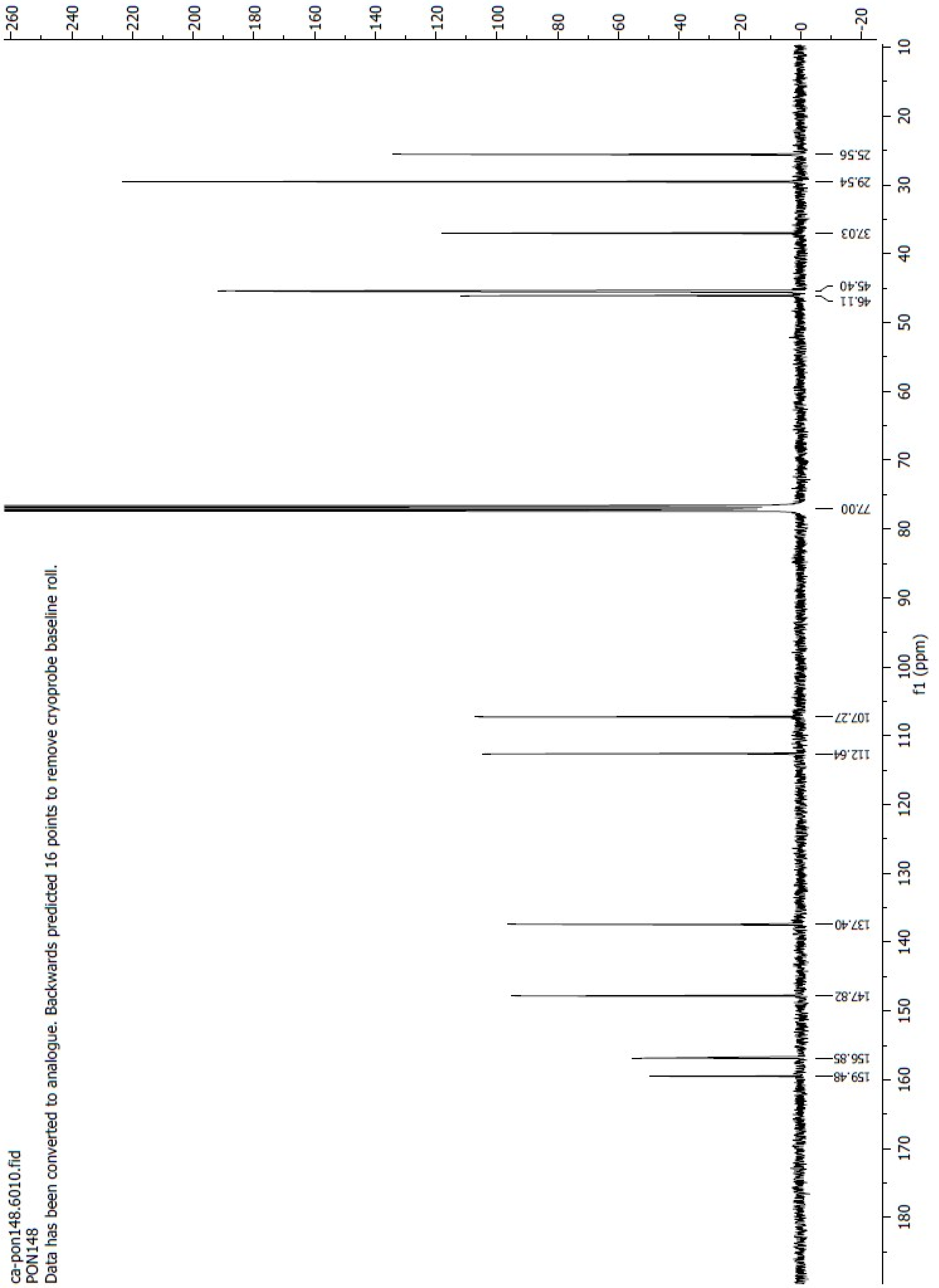

N-((1-(Pyrimidin-2-yl)piperidin-4-yl)methyl)pyrrolidine-1-carboxamide (22)

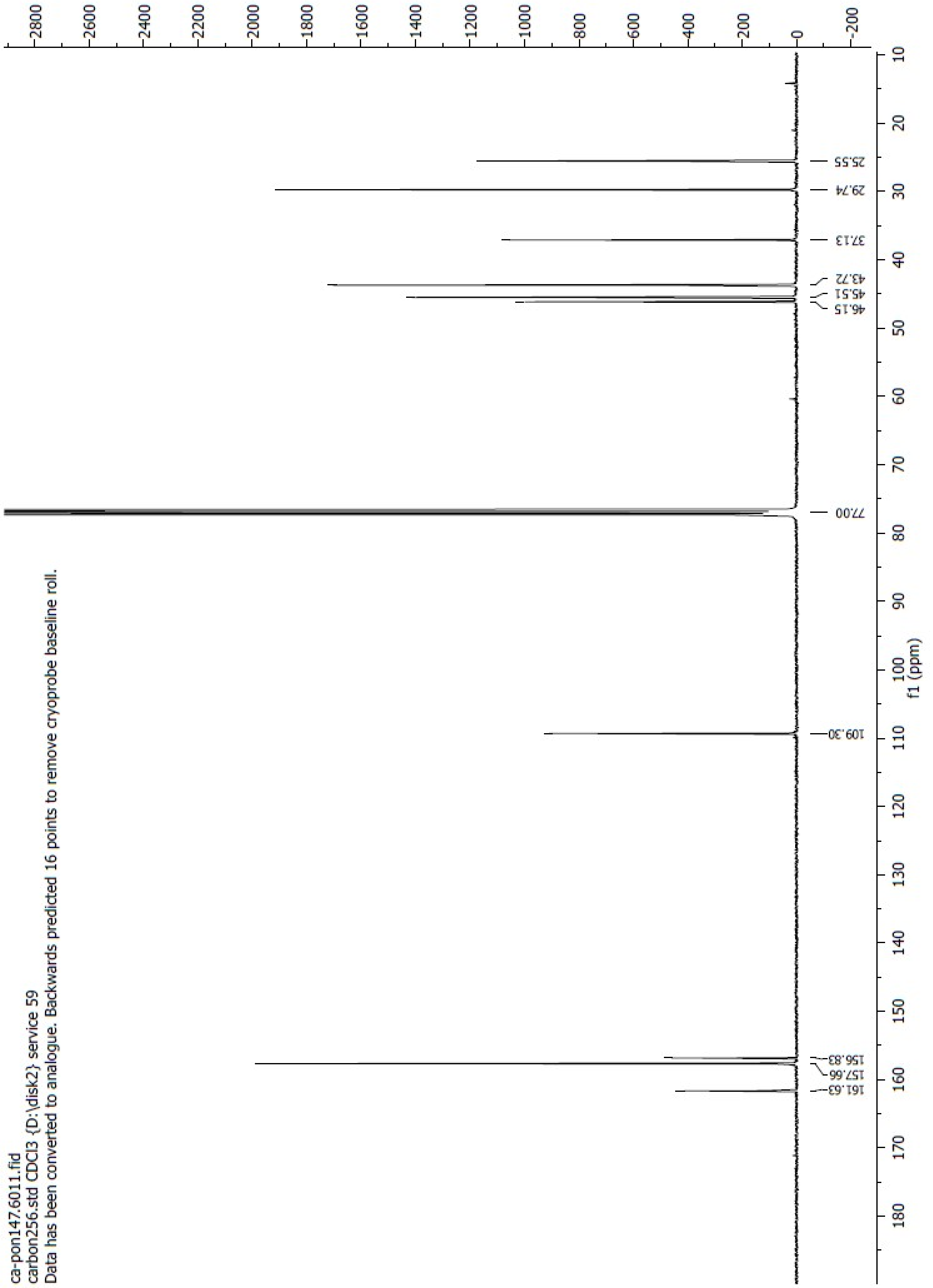

3-(1-(4-Cyanophenyl)piperidin-4-yl)-N-(3-(piperidin-1-yl)phenyl)propanamide (23)

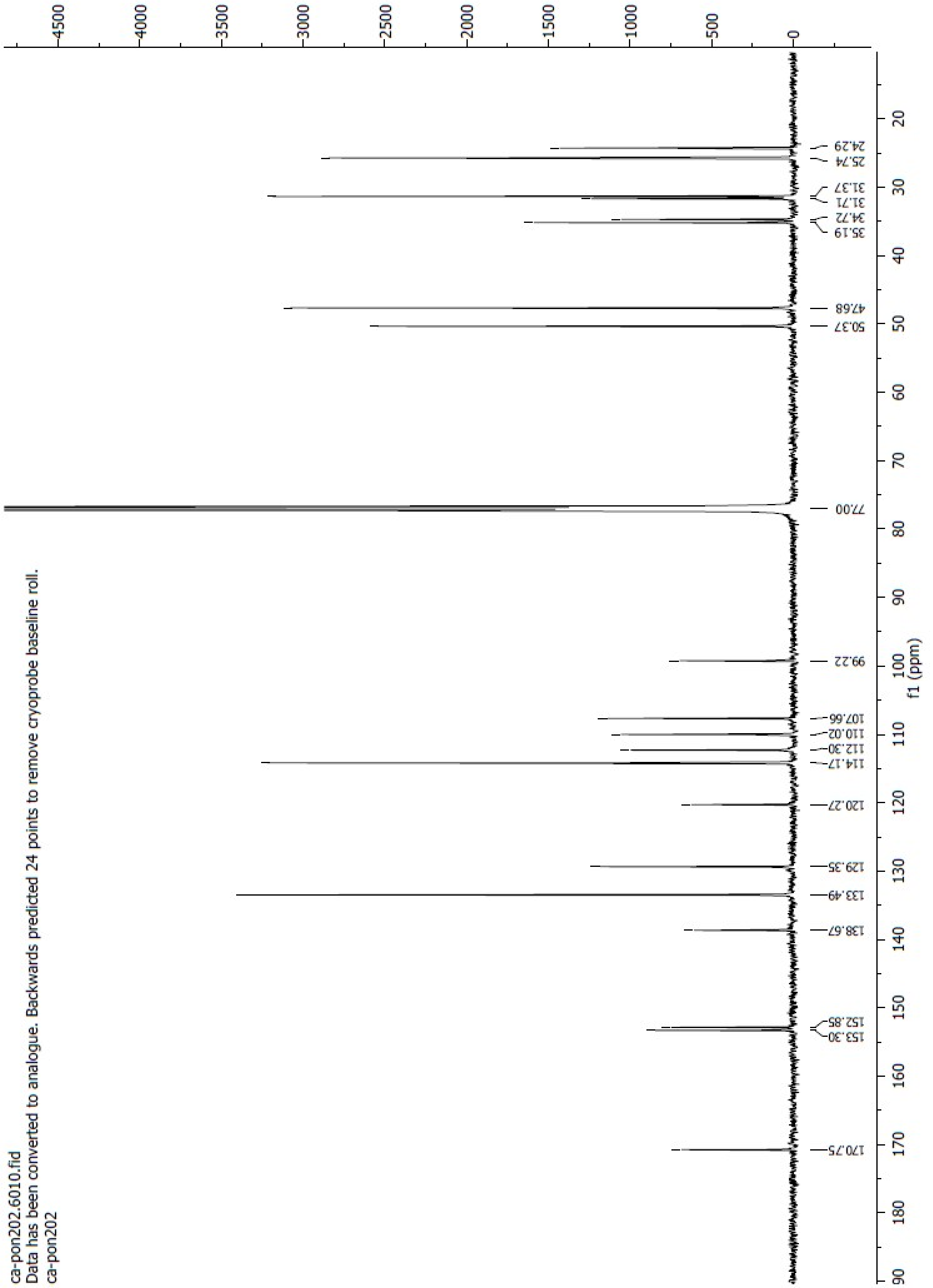

3-(1-(4-Cyanophenyl)piperidin-4-yl)-N-(4-(piperidin-1-yl)benzyl)propanamide (28)

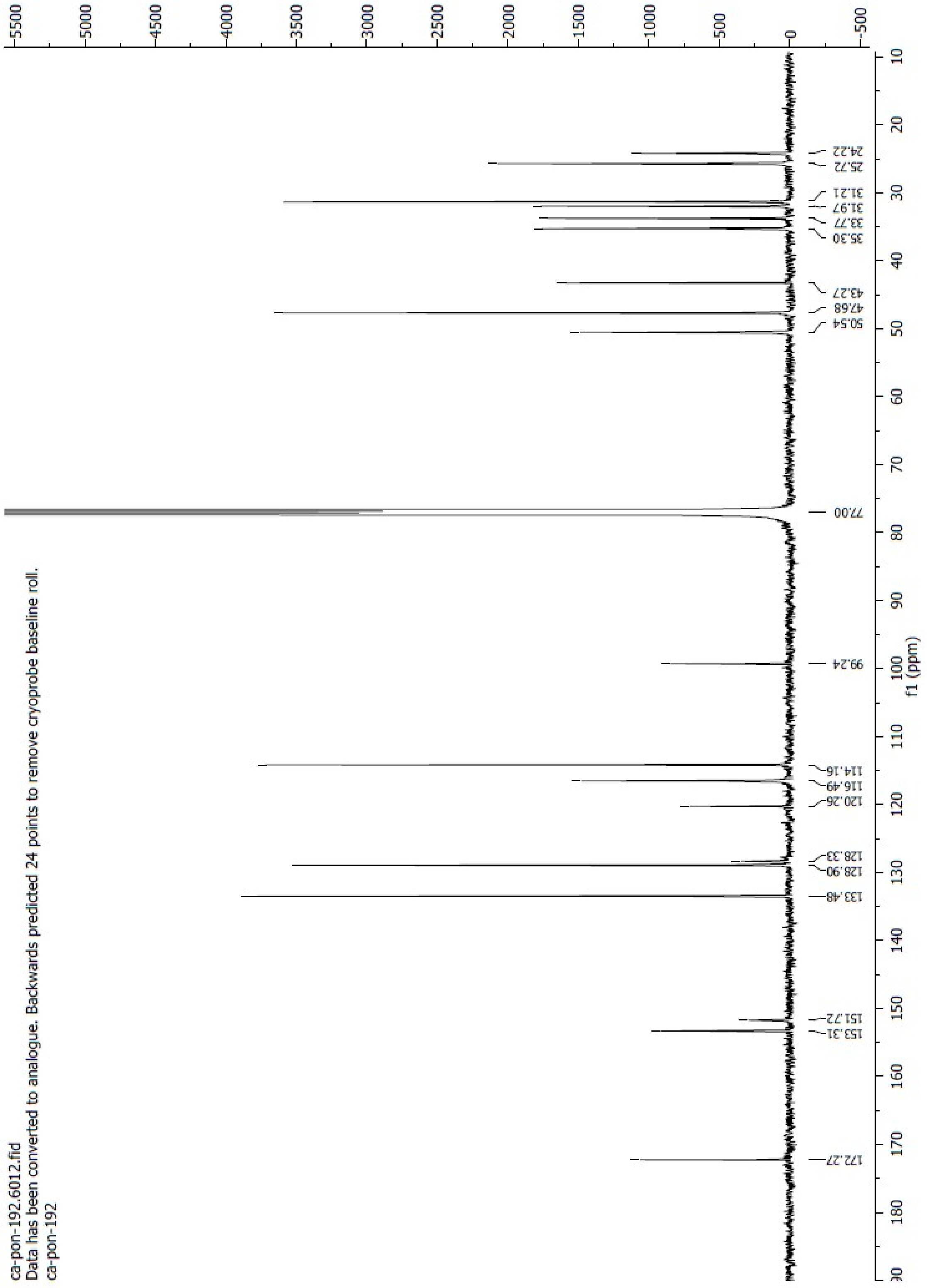

3-(1-(4-Cyanophenyl)piperidin-4-yl)-N-(3-(piperidin-1-yl)benzyl)propanamide (29)

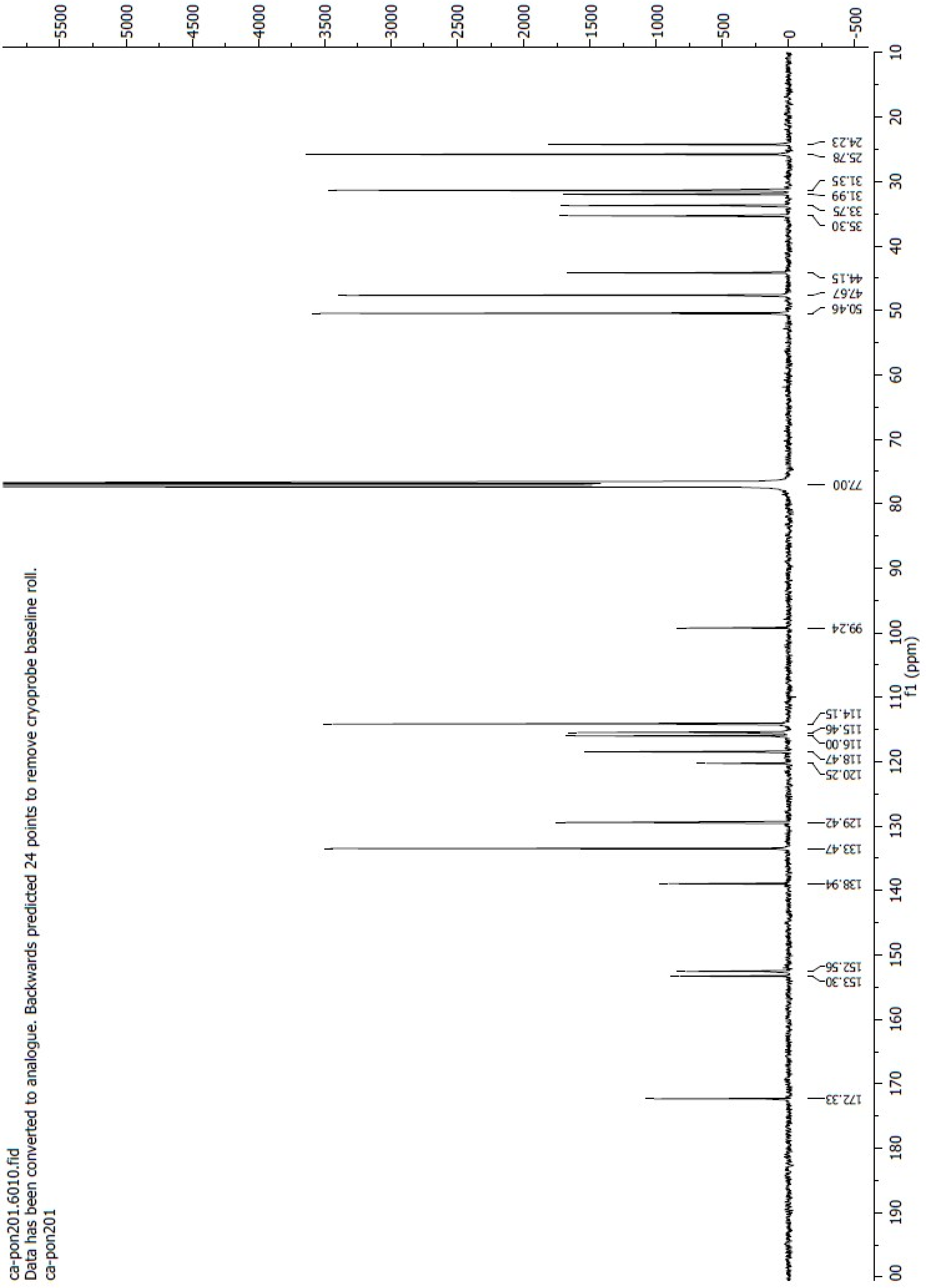

Example LCMS spectrum

Ethyl 4-(4-(3-oxo-3-(pyrrolidin-1-yl)propyl)piperidin-1-yl)benzoate (16)

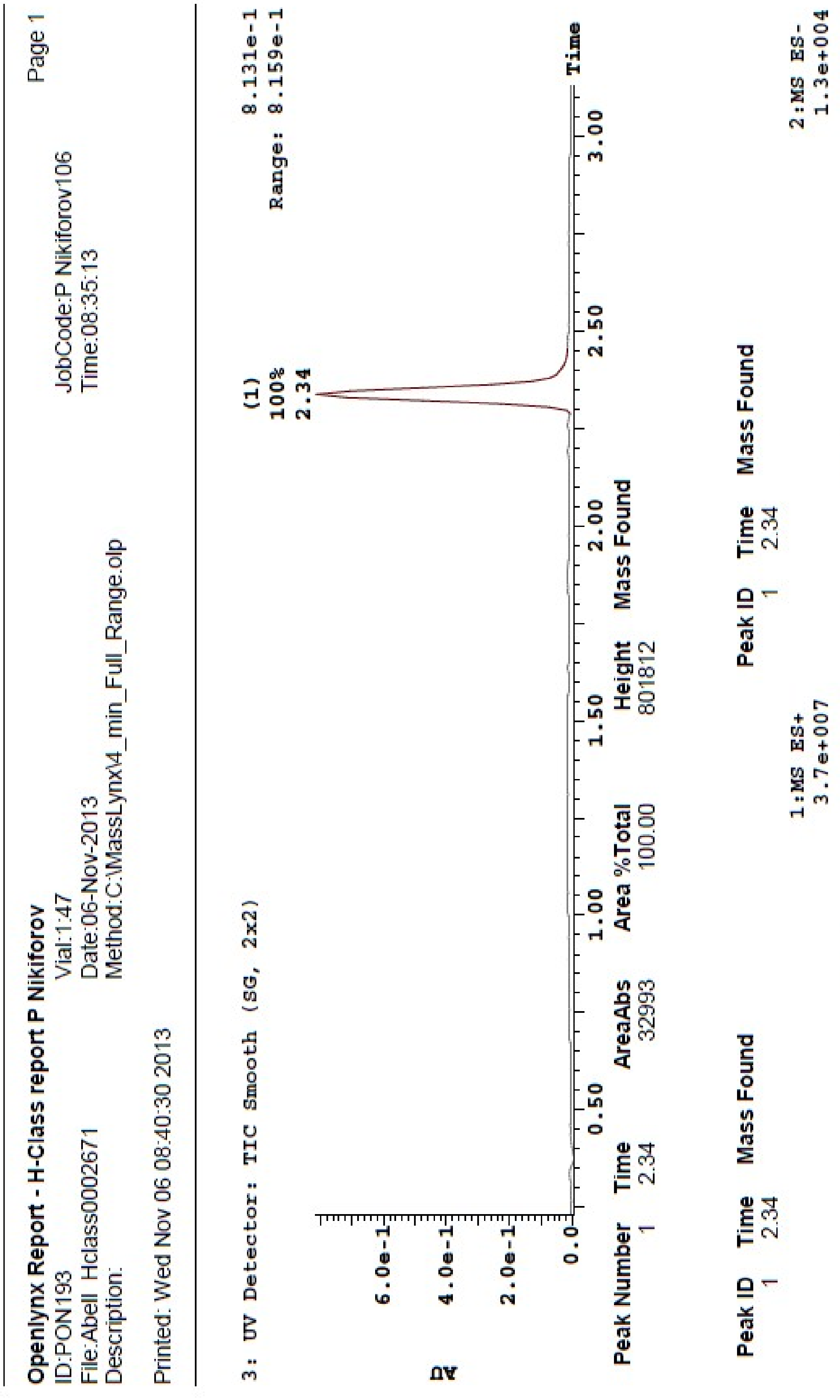

References

1 J. Peränen, M. Rikkonen, M. Hyvönen, L. Kääriäinen, T7 vectors with modified T7lac promoter for expression of proteins in *Escherichia coli*, *Analytical Biochemistry*, 1996, **236**, 371-373.

2 S. Surade, N. Ty, N. Hengrung, B. Lechartier, S. T. Cole, C. Abell and T. L. Blundell, A structure-guided fragment-based approach for the discovery of allosteric inhibitors targeting the lipophilic binding site of transcription factor EthR, *Biochem. J.*, 2014, **458**, 387-394.

3 C. Vonrhein, C. Flensburg, P. Keller, A. Sharff, O. Smart, W. Paciorek, T. Womack, G. Bricogne, Data processing and analysis with the autoPROC toolbox, *Acta Crystallographica*, Section D: Biological Crystallography, 2011, **67**, 293-302.

4 W. Kabsch, Software XDS for image rotation, recognition and crystal symmetry assignment, *Acta Crystallographica*, Section D: Biological Crystallography, 2010, **66**, 125-132.

5 A. G. W. Leslie, H. R. Powell, Processing diffraction data with Mosfilm. In Evolving Methods for Macromolecular Crystallography (Read, R. J. and Sussman, J. L., editors), Springer, Berlin, 2007, 41-51.

6 P. R. Evans, An introduction to data reduction: Space-group determination, scaling and intensity statistics, *Acta Crystallographica*, Section D: Biological Crystallography, 2011, **67**, 282-292.

7 A. J. McCoy, R. W. Grosse-Kunstleve, P. D. Adams, M. D. Winn, L. C. Storoni, and R. J. Read, Phaser crystallographic software, *Journal of Applied Crystallography*, 2007, **40**, 658-674.

8 G. N. Murshudov, A. A. Vagin, E. J. Dodson, Refinement of macromolecular structures by the maximum-likelihood method, *Acta Crystallographica*, Section D: Biological Crystallography, 1997, **53**, 240-255.

9 M. D. Winn, C. C. Ballard, K. D. Cowtan, E. J. Dodson, P. Emsley, P. R. Evans, R. M. Keegan, E. B. Krissinel, A. G. W. Leslie, A. McCoy *et al.* Overview of the CCP4 suite and current developments, *Acta Crystallographica*, Section D: Biological Crystallography, 2011, **67**, 235–242.

10 P. D. Adams, P. V. Afonine, G. Bunkóczi, V. B. Chen, N. Echols, J. J. Headd, L.-W. Hung, S. Jain, G. J. Kapral, R. W. Grosse Kunstleve, A. J. McCoy, N. W.

Moriarty, R. D. Oeffner, R. J. Read, D. C. Richardson, J. S. Richardson, T. C. Terwilliger, P. H. Zwart, The Phenix software for automated determination of macromolecular structures, *Methods* (Amsterdam, Netherlands), 2011, **55**, 94-106.

11 G. Bricogne, E. Blanc, M. Brandl, C. Flensburg, P. Keller, W. Paciorek, P. Roversi, A. Sharff, O. S. Smart, C. Vonrhein, T. O. Womack, BUSTER version X.Y.Z. Cambridge, United Kingdom: Global Phasing Ltd., 2011.

12 A. W. Schuttelkopf, D. M. F. van Aalten, PRODRG: a tool for high-throughput crystallography of protein-ligand complexes, *Acta Crystallographica*, Section D: Biological Crystallography, 2004, **60**, 1355-1363.

13 A. A. Vagin, G. N. Murshudov, B. V. Strokopytov, BLANC: the program suite for protein crystallography, *Journal of Applied Crystallography*, 1998, **31**, 98-102.

14 P. Emsley, B. Lohkamp, W. G. Scott, K. Cowtan, Features and development of Coot, *Acta Crystallographica*, Section D: Biological Crystallography, 2010, **66**, 486-501.

15 J. Engohang-Ndong, D. Baillat, M. Aumercier, F. Bellefontaine, G. S. Besra, C. Locht, A. R. Baulard, EthR, a repressor of the TetR/CamR family implicated in ethionamide resistance in mycobacteria, octamerizes cooperatively on its operator, *Mol. Microbiol.*, 2004, **51**, 175-188.

16 N. Willand, B. Dirié, X. Carette, P. Bifani, A. Singhal, M. Desroses, F. Leroux, E. Willery, V. Mathys, R. Déprez-Poulain, G. Delcroix, F. Frénois, M. Aumercier, C. Locht, V. Villeret, B. Déprez, A. R. Baulard, Synthetic EthR inhibitors boost antituberculous activity of ethionamide, *Nat. Med.*, 2009, **15**, 537-544.

17 A. El-Faham, R. S. Funosas, R. Prohens and F. Albericio, COMU: a safer and more effective replacement for benzotriazole-based uronium coupling reagents, *Chem. Eur. J.*, 2009, **15**, 9404-9416.

18 N. Delsuc, J.-M. Leger, S. Massip and I. Huc, Proteomorphous objects from abiotic backbones, *Angew. Chem. Int. Ed.*, 2007, **46**, 214-217.

19 S. K. Anandan, Z.-Y. Xiao, D. V. Patel, J. S. Ward, *US Pat. Appl. Publ.*, US 20050234033, 2005.

20 R. P. Tangallapally, R. Yendapally, R. E. Lee, A. J. M. Lenaerts, and R. E. Lee, Synthesis and evaluation of cyclic secondary amine substituted phenyl and benzyl nitrofuranyl amides as novel antituberculosis agents, *J. Med. Chem.*, 2005, **48**, 8261-8269.

21 D. Amans, V. Bellosta, C. Dacquet, A. Ktorza, N. Hennuyer, B. Staels, D.-H. Caignard and J. Cossy, Synthesis and evaluation of new polyenic compounds as potential PPARs modulators, *Org. Biomol. Chem.*, 2012, **10**, 6169-6185.

22 J. Cohen, M. Bos, S. Cesco-Cancian, B. Harris, J. Hortenstine, M. Justus, C. Maryanoff, J. Mills, S. Muller, A. Roessler, L. Scott, K. Sorgi, F. Villani, Jr., Robin R. H. Webster and C. Weh, A Practical synthesis of the platelet fibrinogen antagonist, Elarofiban, *Organic Process Research & Development*, 2003, **7**, 866-872.

23 J. A. Grzyb, M. Shen, C. Yoshina-Ishii, W. Chi, R. S. Brown and R. A. Batey, Carbamoylimidazolium and thiocarbamoylimidazolium salts: novel reagents for the synthesis of ureas, thioureas, carbamates, thiocarbamates and amides, *Tetrahedron*, 2005, **61**, 7153-7175.

24 A. M. Birch, P. A. Bradley, J. C. Gill, F. Kerrigan and P. L. Needham, *N*-Substituted (2,3-dihydro-1,4-benzodioxin-2-yl)methylamine derivatives as D_2 antagonists/5-HT_{1A} partial agonists with potential as atypical antipsychotic agents, *J. Med. Chem.*, 1999, **42**, 3342-3355.
